# Mainstreaming Flora Conservation Strategies into the Mitigation Hierarchy to Strengthen Environmental Impact Assessment

**DOI:** 10.1007/s00267-022-01756-y

**Published:** 2022-12-02

**Authors:** Taís Nogueira Fernandes, Fernando Marino Gomes dos Santos, Flávio Dayrell Gontijo, João Alves da Silva Filho, Alexandre Franco Castilho, Luis Enrique Sánchez

**Affiliations:** 1 Department of Mining and Petroleum Engineering, Escola Politécnica, University of São Paulo, Av. Prof. Mello Moraes, 2373, 05508-900 São Paulo, SP Brazil; 2 VALE S/A. Environmental Licensing Management. Mina de Águas Claras. Prédio 1, térreo, Av. Doutor Marco Paulo Simon Jardim, 34.006-200 Nova Lima, MG Brazil; 3 Amplo Engenharia e Gestão de Projetos Ltda, Rua Camões, 28, 30240-270 Belo Horizonte, MG Brazil

**Keywords:** Amazon, Carajás, Endemic flora, Impact avoidance, Mining, Rocky outcrops

## Abstract

The application of the mitigation hierarchy (MH) to mining projects is challenging in situations of locational overlap between endemic flora and mineral deposits. We review flora surveys conducted in connection with the environmental impact assessment of several iron ore mining projects in an area of high degree of endemism in Eastern Amazon to discuss the practical implications of anticipating conservation strategies. Desktop studies and secondary data review were conducted to guide field searches to determine the distribution of endemic flora, resulting in 45 out of 46 endemic plant species having their known distribution extended to new areas. A framework for positioning flora conservation strategies in the MH is presented. Specific habitat requirements and scarce knowledge about endangered and endemic flora species are a conservation obstacle, since essential information to define species conservation strategies may be lacking. We show that anticipating conservation strategies can minimize time-lag uncertainties related to restoration success and biodiversity offsets. The more effort is placed in the preventative steps of the MH, the smaller the time-lag between impact (biodiversity losses) and conservation outcomes (biodiversity gains), decreasing uncertainties and reducing risks to biodiversity.

## Introduction

Preventing harmful impacts is a central purpose of environmental and social impact assessment of development projects. When assessing impacts on biodiversity, the concept of mitigation hierarchy (MH) underpins its practice. The first step in this sequence of preferred actions to mitigate harmful impacts is avoidance, usually requiring changes in project design (Bull et al. 2022), followed by impact minimization through project siting or scheduling, aiming at reducing a project’s footprint or driving it away from important biodiversity features (CSBI 2015). When impacts are unavoidable, or minimization still results in significant residual impacts, remediation and offset measures are then required. Overall, the MH consists of two groups of actions: preventative (avoidance and minimization) and corrective (remediation and offsetting). When applied to biodiversity, the MH aims at achieving no net loss or net positive impact on biodiversity values (IFC 2012). Arguably, no net loss could result from project design that avoids impacts, or could require the coordinated application of the full set of preventative and corrective measures.

Many concerns have been voiced about the actual conservation outcomes obtained by applying the MH. Shortcomings include insufficient attention given to avoidance and minimization, the most effective steps (Phalan et al. 2017), whose application requires the environmental impact assessment (EIA) to be integrated into project design (Sánchez and Franks 2022). When biodiversity is included too late in the project design process and EIAs are carried out after site selection, there is limited potential to design appropriate prevention and minimization measures. Developers are thus criticized for focusing on remediation and offsets even before striving to avoid and minimize impacts, dismissing that only residual impacts should be compensated for (Clare et al. 2011), and that there are unacceptable (thus non-offsetable impacts), such as species extinction (BBOP 2012a). Moreover, corrective measures meet with uncertainties, as remediation and offsets trade immediate and certain losses for long-term and uncertain gains (Bull et al. 2013, Maron et al. 2016). Stressing corrective measures instead of prevention in applying the MH can result in a time lag between degradation and positive effects of those measures (Moilanen & Kotiaho, 2018).

Impact avoidance is particularly challenging for some types of developments such as mines due to the frequent overlap between mineral deposits and important biodiversity features. The expansion of mining over key biodiversity areas entails both direct and indirect impacts, such as forest loss and fragmentation (Siqueira-Gay et al. 2020), that are of particular concern in relatively intact areas (González-González et al. 2021; Siqueira-Gay and Sánchez 2020).

Effective impact avoidance requires robust knowledge about the distribution of biodiversity features that may be affected by a project. Field surveys are usually necessary to collect primary biodiversity data (Gullison et al. 2015) and provide a reliable baseline to assess impacts in support of decisions that could accommodate multiple objectives of environmental conservation and project development.

Topography, climate and weathering, key factors that influence soil formation, are also determinants of unique biodiversity (Velazco et al. 2017), resulting in species with very specific habitat requirements (specialists) that exhibit restricted distribution and low densities (Schemske et al. 1994; Franklin 2010), usually classified as endemic and/or endangered. This is the case of ferruginous crusts overlying iron ore formations that shelter rupestrian vegetation of high endemism that emerged after long periods of evolution with very specific habitat requirements (Jacobi et al. 2008, Skirycz et al. 2014). Limited knowledge about the reproduction and management techniques of the endemic plants of these environments (Jacobi et al. 2007, 2008, Viana et al. 2016, Mota et al. 2018) increases the uncertainties of restoration and offsetting biodiversity values of rupestrian vegetation, making impact avoidance and minimization particularly important.

Here, we firstly review surveys of endemic plants distribution conducted in an area of high degree of flora endemism in connection with the EIA of several iron ore mining projects. Then, we discuss how such information can be used to build a framework for developing tailored conservation strategies for rare, endemic and threatened plants, and discuss the practical implications of anticipating conservation strategies, especially for areas sheltering important biodiversity attributes.

## Methods

### Study Area

**Fig. 1:**
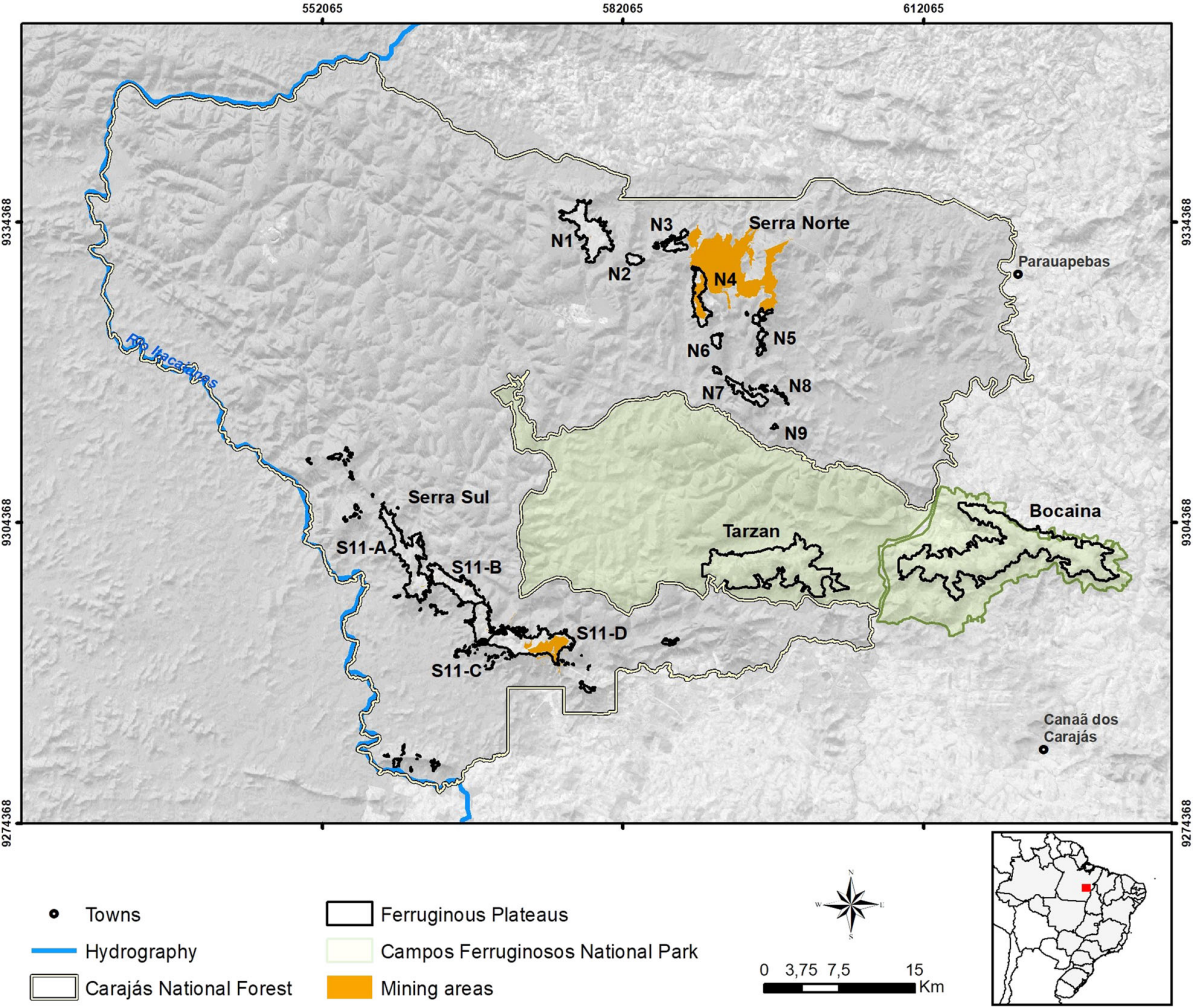
Ferruginous plateaus at Carajás National Forest and Campos Ferruginosos National Park boundaries

All analyses were conducted in an area in Eastern Amazon surrounding and inside the Carajás National Forest (CNF) and Campos Ferruginosos National Park (CFNP) iron ore rock plateaus (Fig. 1), where actual and potential mining areas are located. The study area includes a buffer (Gullison et al. 2015; Dibo et al. 2018) around the iron mineral deposits underlying the mountaintop plateaus.

### Flora Endemic Species

Among the species indicated as endemic of Carajás region (Rodriguésia 2016, 2017, 2018a, 2018b), those whose known occurrence overlaps areas intended for future mining were selected. Forty-six (46) regional endemic flora species of rupestrian outcrops recorded in the Carajás plateaus were selected as targets of new field search efforts (Table 1).

**Table 1:** Endemic species of rupestrian outcrops recorded in the Carajás plateaus selected to field search efforts

Family	Species	Habit
Apocynaceae	*Marsdenia bergii* Morillo	Liana
Araceae	*Philodendron carajasense* E. Gonçalves & Arruda	Herb
Asteraceae	*Cavalcantia glomerata* (G.M.Barroso & R.M.King)	Herb
Asteraceae	*Cavalcantia percymosa* R.M.King & H.Rob.	Herb
Asteraceae	*Lepidaploa paraensis* (H.Hob.) H.Hob.	Shrub
Asteraceae	*Monogereion carajensis* R.M.King & G.M.Barroso	Herb
Asteraceae	*Parapiqueria cavalcantei* R.M.King & H.Rob.	Herb
Blechnaceae	*Blechnum areolatum* V. Dittrich & Salino	Herb
Blechnaceae	*Blechnum longipilosum* V. Dittrich & Salino	Herb
Convolvulaceae	*Ipomoea cavalcantei* D. Austin	Liana
Convolvulaceae	*Ipomoea marabaensis* D. Austin & Secco	Liana
Convolvulaceae	*Ipomoea maurandioides* Meisn	Liana
Cyperaceae	*Bulbostylis cangae* C.S.Nunes & A.Gil	Herb
Cyperaceae	*Eleocharis pedrovianae* C.S. Nunes, A.S.B. Gil & R. Trevisan	Aquatic herb
Cyperaceae	*Hypolytrum paraense* M.Alves & W.W.Thomas	Herb
Eriocaulaceae	*Eriocaulon carajense* Moldenke	Herb
Erythroxylaceae	*Erythroxylum carajasense* (Plowman) J.L.Costa	Shrub
Erythroxyllaceae	*Erythroxylum nelson-rosae* Plowman	Shrub
Fabaceae	*Centrosema carajasense* Cavalc.	Liana
Fabaceae	*Mimosa acutistipula* var. *ferrea* Barneby	Shrub
Fabaceae	*Mimosa skinneri* var. *carajarum* Barneby	Shrub
Gesneriaceae	*Sinningia minina* A.O. Araújo & Chautems	Herb
Isoetaceae	*Isoetes cangae* J.B.S.Pereira, Salino & Stützel	Aquatic herb
Isoetaceae	*Isoetes serracarajensis* J.B.S.Pereira, Salino & Stützel	Aquatic herb
Lentibulariaceae	*Utricularia physoceras* P.Taylor	Herb
Lythraceae	*Cuphea carajasensis* Lourteig	Herb
Melastomataceae	*Brasilianthus carajensis* Almeda & Michelangeli	Herb
Melastomataceae	*Mouriri cearensis* Huber *subsp carajasica* Morley	Shrub
Orobanchaceae	*Buchnera carajasensis* Scatigna & N.Mota	Herb
Picramniaceae	*Picramnia ferrea* Pirani & W.W. Thomas	Shrub
Poaceae	*Axonopus carajasensis* M.N. Bastos	Herb
Poaceae	*Paspalum cangarum* C.O.Moura, P.L.Viana & R.C.Oliveira	Herb
Poaceae	*Paspalum carajasense* S.Denham	Herb
Poaceae	*Sporobolus multiramosus* Boechat & Longhi-Wagner	Herb
Rubiaceae	*Borreria carajasensis* E.L. Cabral & L.M. Miguel	Herb
Rubiaceae	*Borreria elaiosulcata* E.L. Cabral & L.M. Miguel	Herb
Rubiaceae	*Borreria heteranthera* E.L. Cabral & L.M. Miguel	Herb
Rubiaceae	*Borreria paraensis* (Bacigalupo & E.L.Cabral) Delprete	Herb
Rubiaceae	*Borreria semiamplexicaule* (E.L. Cabral) Delprete	Herb
Rubiaceae	*Carajasia cangae* R.M. Salas, E.L. Cabral & Dessein	Herb
Rubiaceae	*Mitracarpus carajasensis* E.L. Cabral, Sobrado & E.B. Souza	Herb
Rubiaceae	*Perama carajensis* J.H. Kirkbr.	Herb
Rutaceae	*Pilocarpus carajaensis Skorupa*	Shrub
Thymelaeaceae	*Daphnopsis filipedunculata* Nevling & Barringer	Shrub
Vitaceae	*Cissus apendiculata* Lombardi	Liana
Xyridaceae	*Xyris brachysepala* Kral	Herb

#### Collating existing knowledge

To systematize knowledge on species distribution, we reviewed literature on endemic Carajás species, vouchers deposited in the herbariums BHCB and MG (acronyms of *Index Herbariorum*), and EIA data (Amplo 2010; Golder 2008, 2010, 2011), as well as recent comprehensive datasets, in particular the “Flora of the cangas of the Serra dos Carajás” (Rodriguésia 2016, 2017, 2018a, 2018b). Only the records confirmed by botanical experts and herbariums were used.

### Searching Species New Records

Flora surveys were planned according to satellite vegetation mapping and species distribution microhabitat. Predictive modeling can minimize field surveys efforts by ranking potential target areas by their environmental similarity with the study area (Gogol-Prokurat 2011). Results of similarity studies guide searches and field surveys, as well as indicate areas to be preserved or restored as offsets. However, determining the potential spatial distribution of plants does not always need help of predictive modeling, such as in the study area. Because these endemic plant species are extremely fine-tuned to their habitat type, in a simple landscape analysis, we mapped areas in the region that present altitude and vegetation cover similar to those where species have already been found, to locate potential areas and direct field searches. When available, phenology and ecology data also were used, allowing field work planning to potentiate efforts in the flowering season and to specific habitats.

The field searches extended up to 200 km away from the CNF and CFNP, between December 2015 and March 2018, totaling 132 field days, distributed in 15 field trips lasting 9 days with teams formed by 8 professionals each. The data collection methodology adopted was focused on the microhabitats and target species presence-absence records, associated with phenological data annotations. All new records obtained in the field searches were added to initial records for new quantitative analysis.

During that process, herbariums receiving exsiccate collections were frequently visited, specialists were consulted to validate identifications for data collection and species identification or description.

### Extent of Occurrence (EOO) and Area of Occupancy (AOO)

The open-source tool for rapid red list assessments GeoCat (Bachman et al. 2011) was used to calculate AOO and EOO, with a 2 km² grid, following the IUCN (2012) standard (EOO calculated by applying a Minimum Convex Polygon - MCP). The species EOO and AOO were calculated for each species before and after the field searches. Since EOO includes discontinuities in the habitat and species areas occupation (Gaston and Fuller 2009), AOO measures were also performed. Following the IUCN (2012) B1 and B2 criteria calculation method, no inferred or modeled records were considered, only those confirmed.

## Results

Species distribution knowledge was updated, and 45 out of the 46 species had their known distribution extended to new areas as a result of field surveys (Online Resource 1 and 2). The distribution of just one species (*Isoetes cangae*) remained unchanged.

Prior to the search effort, 10 out of 46 species would meet the B1 criteria (IUCN 2012) as critically endangered (CR) (EOO < 100 km²) and the remaining 35 would be classified as Endangered (EOO < 5000 km²). After updating the distribution knowledge, only two species remained as CR (*Isoetes cangae* and *Carajasia cangae*), 24 as endangered, 15 as Vulnerable (EOO < 20,000 km²) and 5 no longer meet any of the criteria mentioned. Considering the B2 criteria, the updating of species that fit the classification as CR (AOO < 10 km²) changes from four to one species (*Isoetes cangae*), and the remaining 45 species, even with the expansion of distribution, remain as EN (AOO < 500 km²) (Online Resource 1). Our findings corroborate and update those of Giulietti et al. (2019).

*Isoetes cangae* is the only species whose occupancy area is completely overlapped by an operating mine. In the environmental licensing of the project, a set-aside avoidance area was created, as required by the environmental agencies, to circumvent the species occupancy area (IBAMA 2016; ICMBio 2018). Furthermore, agreements were signed with a research institution and universities to execute a specific *Isoetes cangae* conservation plan, including ecological and genetic studies, reproduction, flora rescue, germplasm bank deposit, multiplication and relocation (Caldeira et al. 2018, Caldeira et al. 2021; Campos et al. 2021a; Campos et al. 2021b; Cavalheiro-Filho et al. 2021; Dalapicolla et al. 2021; Nunes et al. 2018; Santos et al. 2019; Santos et al. 2020; Zandonadi et al. 2019; Zandonadi et al. 2021, and many other researches).

## Discussion: Implications for Environmental Impact Assessment Practice

A common weakness in EIA is the scarcity of floristic data (Ritter et al. 2017) and insufficient survey effort to find threatened flora species (Garrard et al. 2014). The extreme enlargements of species EOO and AOO found here are probably the result of species records limited to the projects areas, absence of surveys distributed over the year, and sampling designs that disregard systematic plant population surveys following IUCN red threatened criteria (Keith 2000).

The expansion of knowledge about species in the area helps to identify the non-offsetable attributes of biodiversity, which would consequently cause fatal failure in the project. This can happen especially in critical habitats, where evolutionary processes have resulted in restricted endemism, with species that only occur in the project area. Although relevant for all other biodiversity attributes, the expansion of knowledge and anticipation of solutions for biodiversity that are difficult to manage (endemic species flora reproduction, multiplication, etc.) is a *sine qua non* condition. The success (or failure) of anticipated conservation actions will often determine the future of the project, whether or not it can proceed. The complete success of the anticipation actions may allow the implementation of the project in the area, or indicate the need for engineering adjustments, restricting conservation areas to avoid the impact on non-offsetable attributes. Likewise, the failure of conservation actions can prevent the implementation of the entire project due to the net losses that would be caused by it (the condition of not being able to manage the species keeps them as non-offsetable feature). Although firmly grounded on a conservationist approach, the framework signals a possibility of conciliation (Pilgrim et al. 2012) in cases of overlapping areas of mineral deposits and non-offsetable biodiversity.

**Fig. 2:**
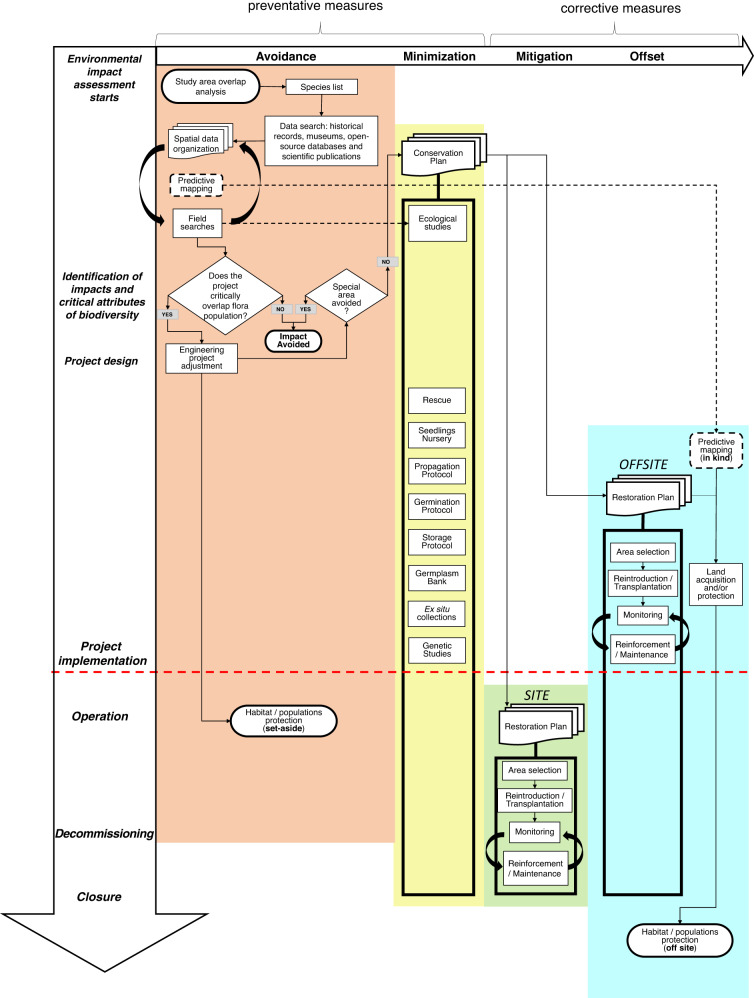
Flora conservation strategies mitigation hierarchy framework. Avoidance: desktop studies, planning for data collection, field work, environmental assessment, and engineering project design. Minimization: implementation of conservation plan for species threatened by the project. Restoration: the knowledge of conservation plan (minimization) is applied in restoration actions at both at the project and offset areas. Offset: new areas can be acquired and protected to preserve species outside the project area, including those identified by predictive modeling (in kind). If they need restoration, the sooner the actions start, the sooner there will be biodiversity gains

Based on practical guidance (BBOP 2012b; IFC 2012; CSBI 2015; Gullison et al. 2015) and literature (Thorne et al. 2014; Tallis et al. 2015; Phalan et al. 2017, among others), we propose that flora conservation strategies can be mainstreamed into the MH to guide EIA planning as presented in Fig. 2. All literature review, data organization, identification of knowledge gaps, planning primary data collection, field research and spatial analysis, with respective input into engineering projects and corresponding design rearrangements are activities that fit into the avoidance step. Data feedback and reanalysis must be considered. All possible risks to biodiversity must be identified and the respective conservation plans prepared and initiated in the minimization step. A conservation and a restoration plan, as well as an offset plan, if applicable, are essential components of a Biodiversity Action Plan to be implemented as the project proceeds.

In the proposed framework, searches for species are linked to the objectives of the Biodiversity Action Plan, which is expected to include all initiatives of the Conservation and Restoration plans. Although the Conservation Plan itself contains actions to minimize impacts, its preparation should start alongside field searches, because at this moment, information about the natural history and ecology of the species must be recorded as surveys and specimens` collection progresses. Other actions can include the development of nursery procedures, population genetic studies and the elaboration of the respective protocols, which will guide the process of species storage, germination, multiplication, and reproduction.

Restoration actions can be of two kinds: those applicable to the project site, usually initiated later in the project cycle, and those applicable offsite, aiming at offsetting unavoidable impacts. Even though restoration is a corrective measure, developing the Restoration Plan requires information and knowledge of the species in their natural habitat (field searches and data records) and the results of the Conservation Plan (for example, for planning seedling planting and developing protocols, seeking to increase the success of restoration). Learning and knowledge, if properly managed (Sánchez and Mitchell 2017) entails a virtuous circle, connecting all stages, with information from the monitoring and reinforcement of restoration areas, helping to test and improve the protocols of the Conservation Plan and monitoring the genetic health of populations in the restoration area.

If the plan includes offsite restoration actions, these should be initiated as soon as possible by searching the availability of degraded areas and engaging with stakeholders (Rosa et al. 2018), but here we do not consider that the developer is going straight to offsetting without considering the initial MH steps, but rather anticipating conservation actions for specific biodiversity features.

Efforts aimed at establishing the baseline for EIAs generally focus on the project area and immediate surroundings (Emberton et al. 2018) not considering a broader study area in its landscape context, thus impairing the identification of relevant areas and associated species. Adopting a landscape-level approach is essential for biodiversity conservation because it facilitates that biologically and ecologically important features remain the core conservation elements over time (Kiesecker et al. 2010).

Ideally, baseline data should be collected several years before mining commences to provide a robust data set—the duration depending on factors such as seasonality, magnitude of expected impact and the target species’ ecology and lifespan (Ritter et al. 2017). Knowledge about how to synchronize ecological studies with the project schedule is fundamental for an appropriate risk management of biodiversity and project, as delays in project delivery can have huge financial implications (CSBI 2015). Improving knowledge about species distribution, AOO and EOO through field surveys enables better consideration of alternatives in project design and contributes to the conservation efforts to be directed to those that have the potential to be affected by future project implementation. Except for mine pits, it is assumed that at early planning stage, the location of waste rock piles and tailings storage facilities, as well as of ancillary installations and any other project component is yet to be established and can be adjusted to escape critical biodiversity targets (Sánchez and Franks 2022).

**Fig. 3:**
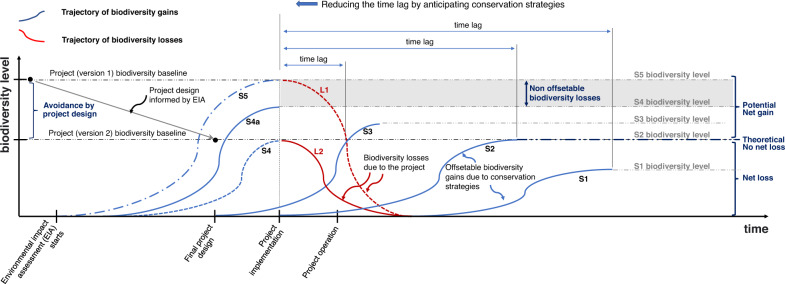
Anticipating conservation strategies. In this figure, P1 is the original project, designed to maximize economic return, P2 is the revised project, designed to (i) avoid non-offsetable impacts on biodiversity values, and (ii) minimize impacts on biodiversity values, S1 to S5 are conservation strategies, L1 and L2 are biodiversity losses resulting respectively from P1 and P2. Strategies S1 to S2 apply to P2, while strategy S5 applies to P1 only. S1 is a conservation strategy initiating after impacts, e.g., restoration of a mine site, meaning a long time-lag between losses and uncertain gain, that accrue over time. This strategy implies a long period of biodiversity deficit in which ecosystems do not play their full ecological role nor provide ecosystem services. If full restoration fails, the outcome is net loss. S2 is similar to S1, but is initiated earlier, simultaneously with impacting activities (i.e., after project approval), increasing the chance of achieving no net loss and reducing the time lag, but may uncertainties remain, as well as a period of biodiversity deficit. S3 is a conservation strategy initiating before impact, shortly after final project designing, adjusting the original engineering project. Early implementation of the strategy further reduces the time lag and is more likely to conduce to net gain. S4 is a conservation strategy capable of not only matching, but also of surpassing (S4a) future losses before impact takes place aiming at eliminating the time lag. For critical biodiversity, conservation strategy S5 is conceivably applicable to biodiversity values initially assessed as non-offsetable, such as in cases of occurrence of endemic species. The only way for a project to advance on areas containing non-offsetable attributes of biodiversity should be successfully anticipating the like-for-like compensation before its implementation.

For the effective application of the framework, early implementation, by anticipating all MH steps, contributes to reducing uncertainties about the success of corrective measures. The more the MH steps are anticipated, the smaller the time-lag between impact (biodiversity losses) and conservation outcomes (biodiversity gains), decreasing, or even nullifying, the offset uncertainties (Fig. 3).

The current approach to dealing with biodiversity impacts is to offset vegetation clearing by protecting and/or restoring equivalent habitats, and to rehabilitate mined. However, specific habitat requirements and scarce knowledge about endangered and endemic flora species are limitations to reach measurable conservation outcomes, since knowledge on reproduction, phenology, germination, habitat requirements and other issues is necessary to define conservation strategies (Morris and Doak 2002; Merow et al. 2014). Therefore, there is high uncertainty about the achievement of conservation outcomes, especially when the feasibility of restoration is unknown.

Therefore, it is necessary to consider the risk that restoration will not achieve its goals. Anticipating restoration offsets reduces the risks of an unfair exchange for biodiversity, as it can shorten the time between biodiversity losses and conservation outcomes, and anticipate strategies changes in the experiments, in case they have negative results. Increasing the chances of success by anticipating actions can also raise confidence of decision-makers and stakeholders in environmental licensing.

When avoidance is dismissed, or if it takes too long to be considered, subsequent actions to prevent or remediate impacts are likely to be more expensive, time-consuming, and less efficient, leading to a decrease in cost-benefit (CSBI 2013) and possible project delays. In those situations, biodiversity risks cannot be eliminated but can (and should) be managed (Hummel et al. 2009). The only way to manage non offsetable biodiversity attributes is the success of anticipating conservation strategies (Fig. 3).

Anticipating mitigation is a strategy to reduce uncertainties attributed to the time delay between the impact and conservation outcomes from mitigation. One such approach consists of acquiring and restoring areas before the impacts happen, through mitigation banks (McKenney and Kiesecker 2010, Gardner and von Hase 2012, Poudel et al. 2019), but in addition to requiring enabling legislation, we posit that they are hardly applicable to critical habitats. Rupestrian vegetation associated with iron ore outcrops are a very specific biodiversity feature that calls for tailored strategies to effect the goals of the MH, especially if a project may result in species extinction, a non-offsetable condition.

When restoration accumulates gains and habitat condition starts to recover, conservation actions go towards losses balance (Moilanen and Kotiaho 2020) but such a state may require a long time span to be reached (CSBI 2013; Moilanen and Kotiaho 2018). We venture to say that if reproducing and maintaining habitat and biodiversity of very specific ecological requirements were easy and fast, they would not be threatened. As the knowledge about species germination, reproduction, and maintenance techniques advances, the better the solutions and practices for environmental restoration.

Since the iron ore outcrops of CNF (Fig. 1) have been the subject of studies and mining projects since the 1980s, lessons learned should be applied to the region, but can also offer insights and recommendations for EIA wherever development projects encroaches over areas of endemic flora. We postulate here the anticipation of all MH steps (Fig. 3) described in the framework (Fig. 2) to reconcile biodiversity conservancy and project development.

## Conclusion

As effective application of the MH requires robust biodiversity information, we showed that, in highly biodiverse areas, efforts aimed at establishing the baseline for EIAs must consider a broad study area, in its landscape context, as well as appropriate time frames for conducting field surveys. The search design used in this research made possible that 45 out of the 46 endemic plant species of Carajás had their known distribution extended to new areas.

We postulate that anticipating conservation strategies can minimize time-lag offset uncertainties and, for species with very specific habitat requirements, conservation strategies should not only start long before project implementation, but their outcomes should match (or surpass) predicted future losses before project impactful actions start.

We show how a rigorous process to collecting baseline data can expand knowledge and help design appropriate conservation strategies for endemic flora at an earlier project stage. The framework can be adapted for application to other critical groups or assemblies.

## Supplementary Information

Supplementary Information

Supplementary Information
