# Supplementary material for: Mainstreaming Flora Conservation Strategies into the Mitigation Hierarchy to Strengthen Environmental Impact Assessment
Source: Environ Manage. 2022 Dec 2;71(2):483–93. doi: 10.1007/s00267-022-01756-y (PMC9892156; doi:10.1007/s00267-022-01756-y)
Supplement: Supplementary file 2 — Supplementary Information [file 267_2022_1756_MOESM2_ESM.pdf]

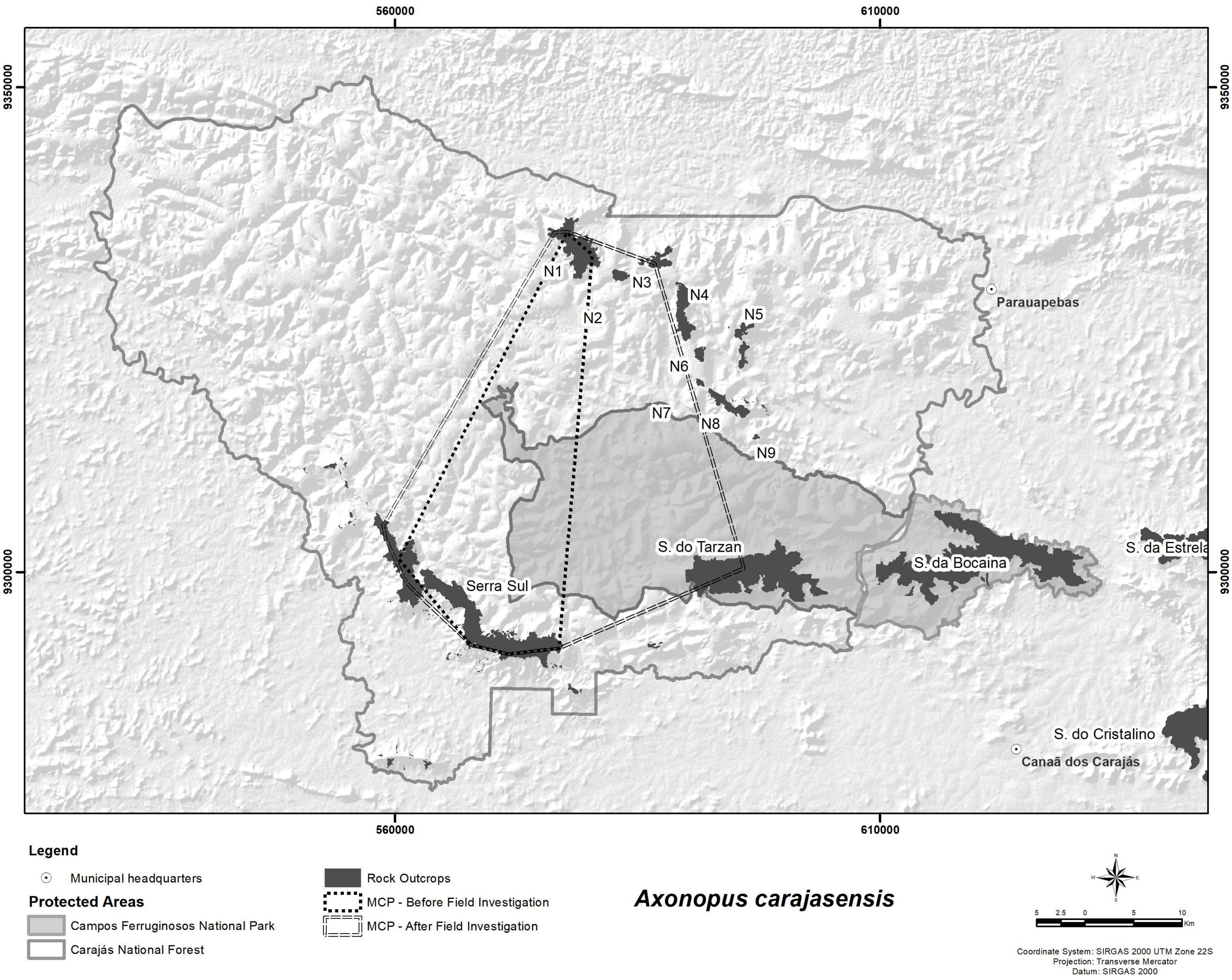
560000
610000
9350000
9300000
N1
N2
N3
N4
N5
N6
N7
N8
N9
Parauapebas
Serra Sul
S. do Tarzan
S. da Bocaina
S. da Estrela
S. do Cristalino
Canaã dos Carajás
Legend
Municipal headquarters
Protected Areas
Campos Ferruginosos National Park
Carajás National Forest
Rock Outcrops
MCP - Before Field Investigation
MCP - After Field Investigation
Axonopus carajasensis
5 2.5 0 5 10
Km
Coordinate System: SIRGAS 2000 UTM Zone 22S
Projection: Transverse Mercator
Datum: SIRGAS 2000

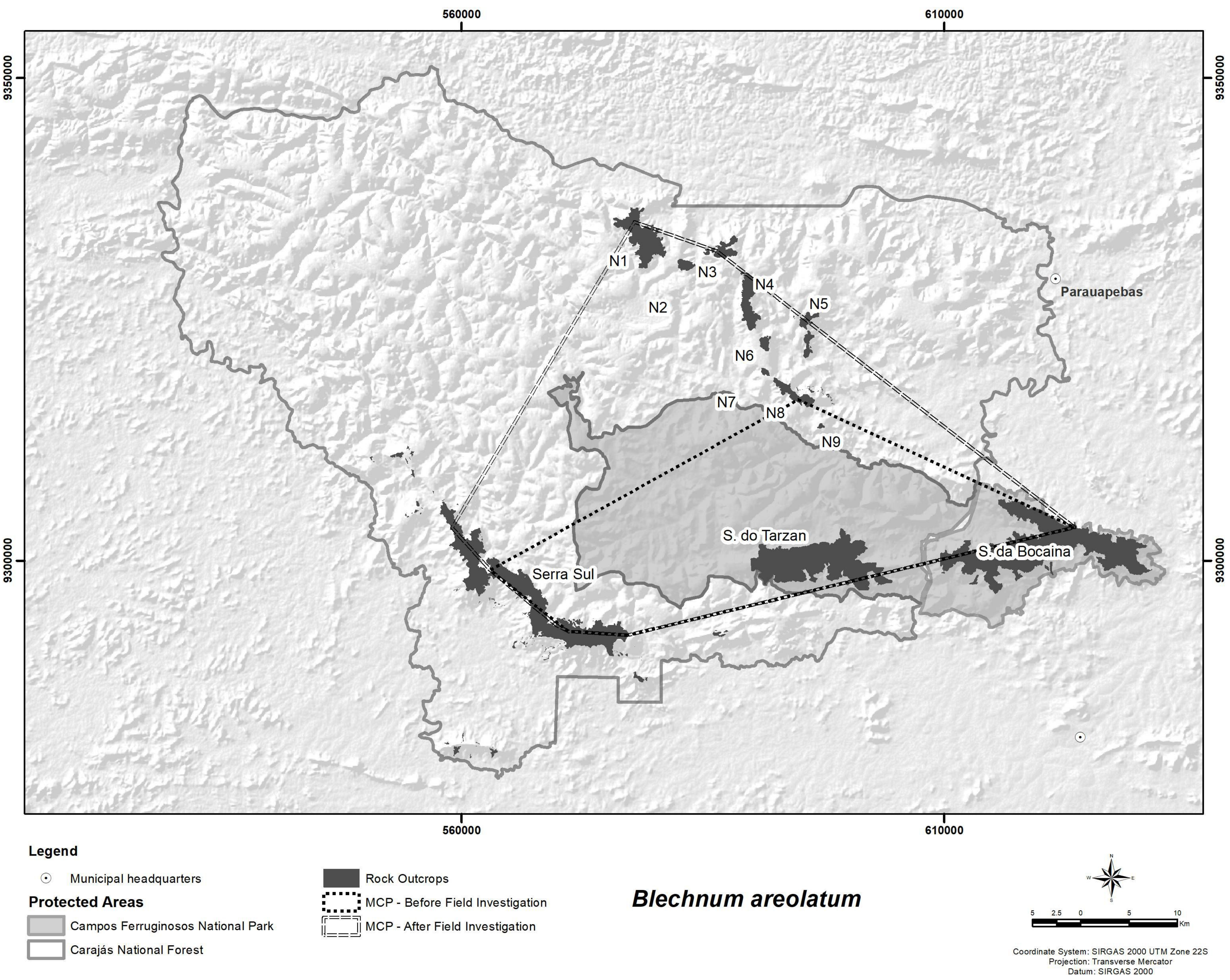

560000
610000
9350000
9300000
N1
N2
N3
N4
N5
N6
N7
N8
N9
Parauapebas
S. do Tarzan
S. da Bocaina
Serra Sul
Legend
Municipal headquarters
Protected Areas
Campos Ferruginosos National Park
Carajás National Forest
Rock Outcrops
MCP - Before Field Investigation
MCP - After Field Investigation
Blechnum areolatum
5 2.5 0 5 10
Km
Coordinate System: SIRGAS 2000 UTM Zone 22S
Projection: Transverse Mercator
Datum: SIRGAS 2000

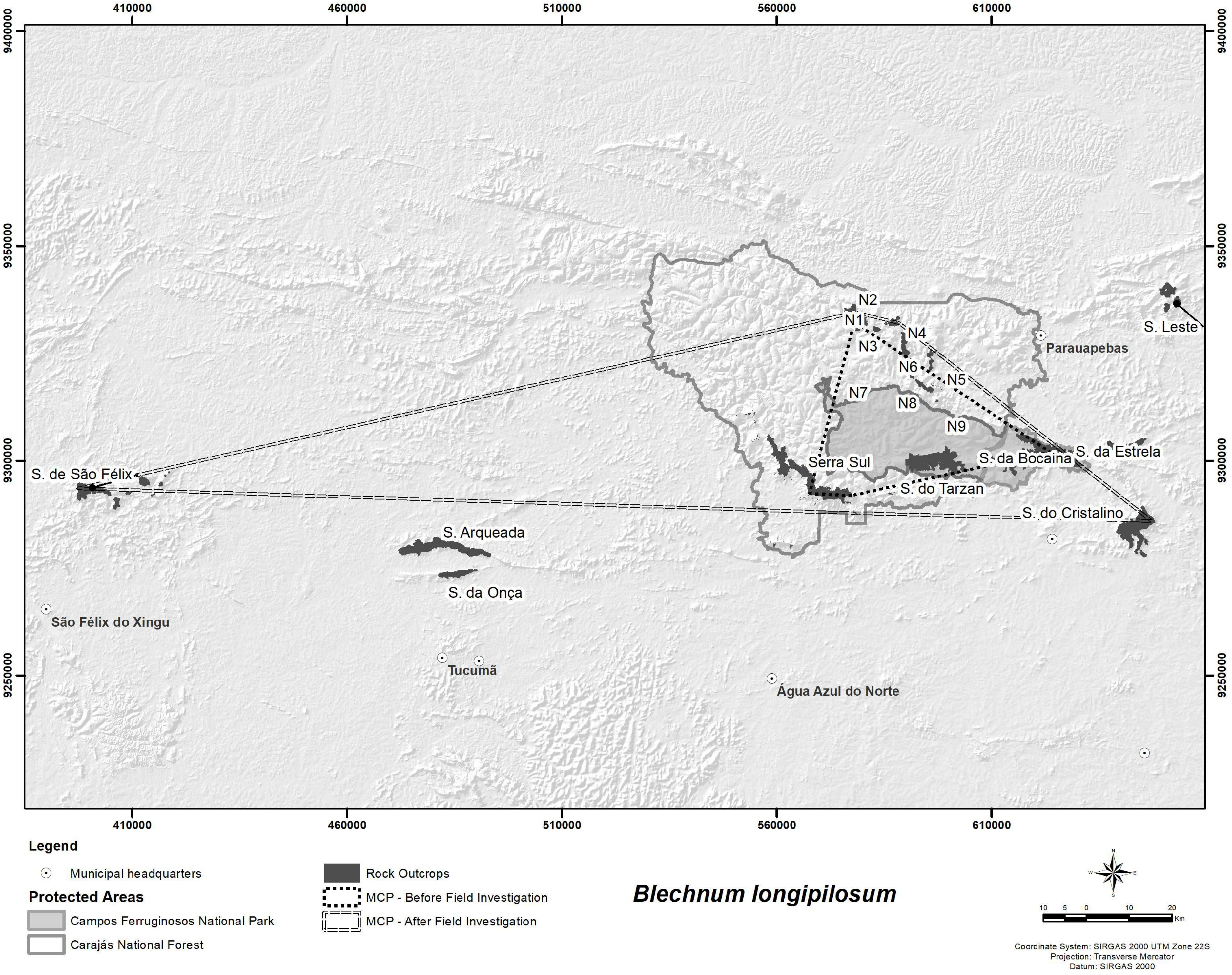

410000
460000
510000
560000
610000
9400000
9350000
9300000
9250000
N2
N1
N4
N3
N6
N5
N7
N8
N9
S. Leste
Parauapebas
S. da Estrela
S. da Bocaina
Serra Sul
S. do Tarzan
S. do Cristalino
S. de São Félix
S. Arqueada
S. da Onça
São Félix do Xingu
Tucumã
Água Azul do Norte
Legend
Municipal headquarters
Protected Areas
Campos Ferruginosos National Park
Carajás National Forest
Rock Outcrops
MCP - Before Field Investigation
MCP - After Field Investigation
Blechnum longipilosum
N
W
E
S
10 5 0 10 20
Km
Coordinate System: SIRGAS 2000 UTM Zone 22S
Projection: Transverse Mercator
Datum: SIRGAS 2000

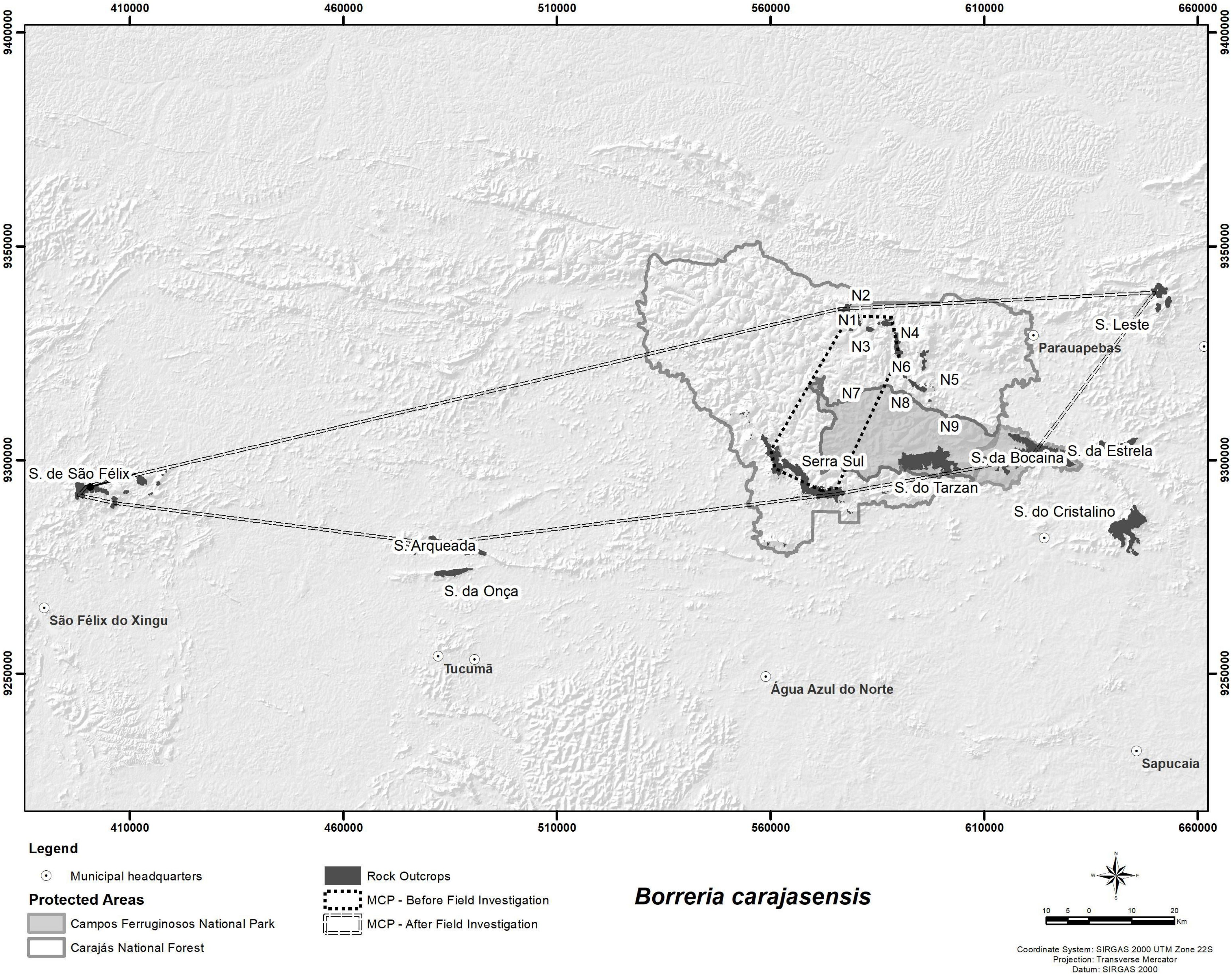
410000
460000
510000
560000
610000
660000
9400000
9350000
9300000
9250000
N2
N1
N4
N3
N6
N5
N7
N8
N9
S. Leste
Parauapebas
Serra Sul
S. da Bocaina
S. da Estrela
S. do Tarzan
S. de São Félix
S. Arqueada
S. da Onça
S. do Cristalino
São Félix do Xingu
Tucumã
Água Azul do Norte
Sapucaia
Legend
Municipal headquarters
Protected Areas
Campos Ferruginosos National Park
Carajás National Forest
Rock Outcrops
MCP - Before Field Investigation
MCP - After Field Investigation
Borreria carajasensis
10 5 0 10 20
Km
Coordinate System: SIRGAS 2000 UTM Zone 22S
Projection: Transverse Mercator
Datum: SIRGAS 2000

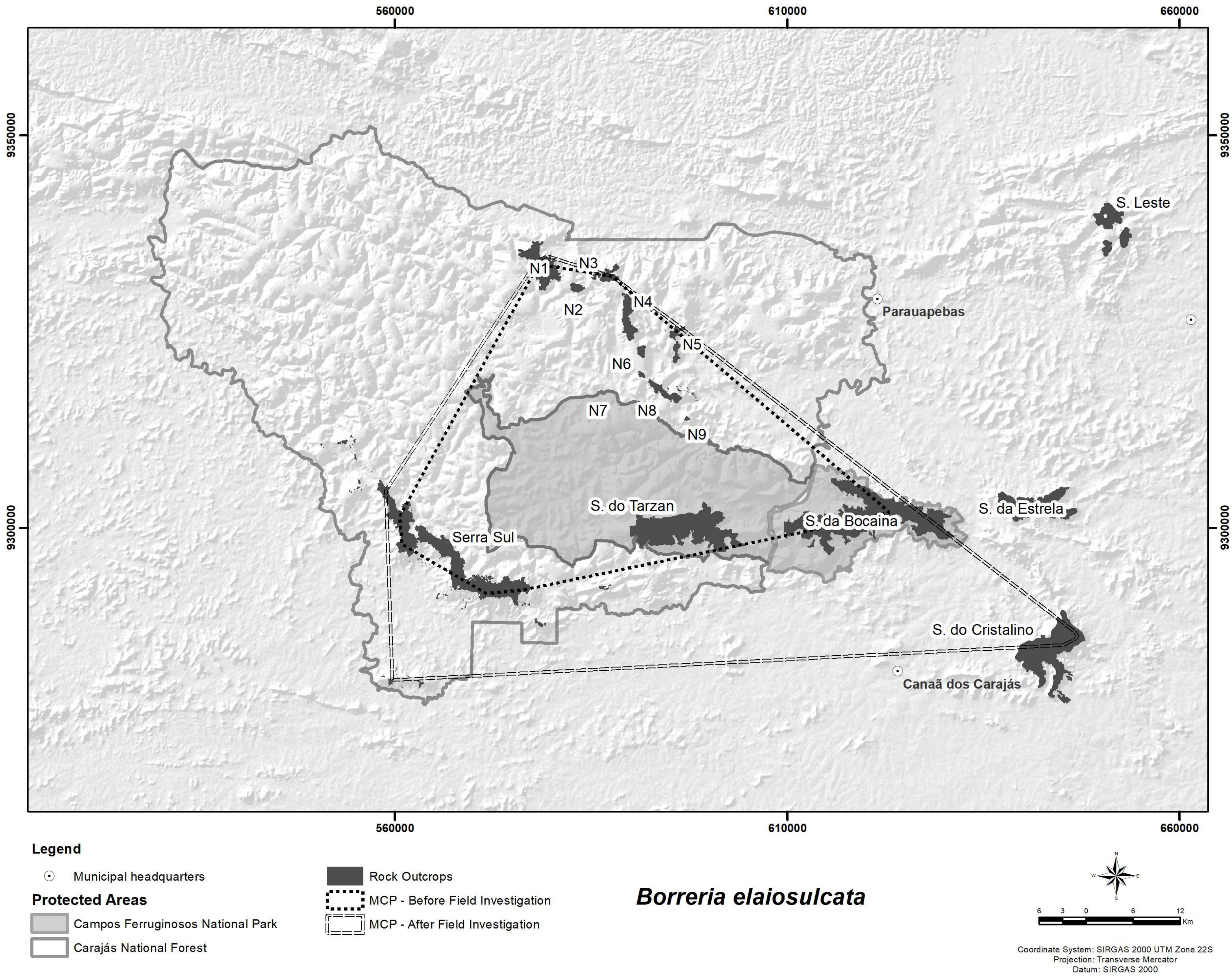
560000
610000
660000
9350000
9300000
S. Leste
N1
N3
N2
N4
N5
N6
N7
N8
N9
Parauapebas
S. do Tarzan
S. da Estrela
S. da Bocaina
Serra Sul
S. do Cristalino
Canaã dos Carajás
Legend
Municipal headquarters
Protected Areas
Campos Ferruginosos National Park
Carajás National Forest
Rock Outcrops
MCP - Before Field Investigation
MCP - After Field Investigation
Borreria elaiosulcata
Km
Coordinate System: SIRGAS 2000 UTM Zone 22S
Projection: Transverse Mercator
Datum: SIRGAS 2000

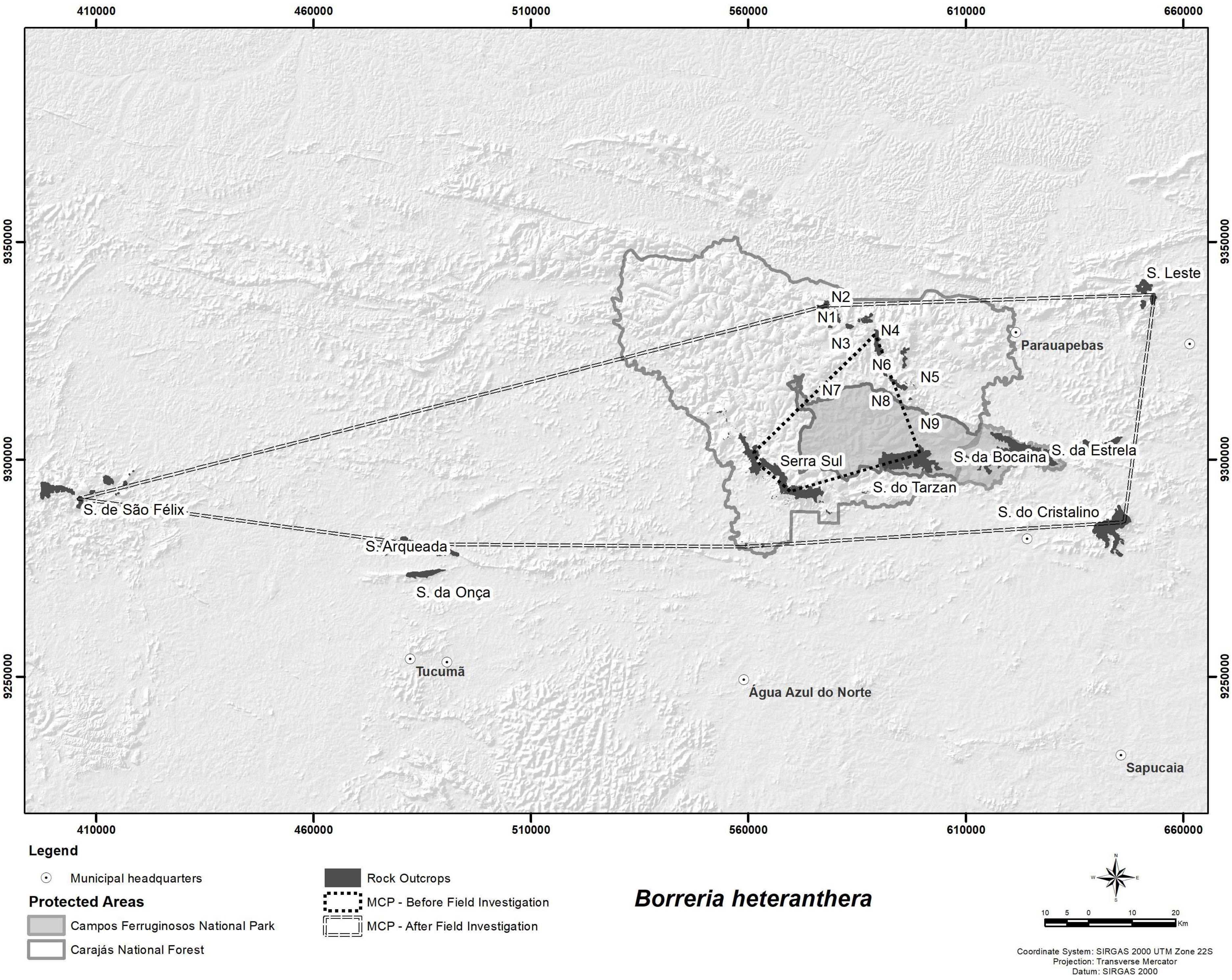
410000
460000
510000
560000
610000
660000
9350000
9300000
9250000
S. Leste
N2
N1
N4
N3
Parauapebas
N6
N5
N7
N8
N9
Serra Sul
S. da Bocaina
S. da Estrela
S. do Tarzan
S. de São Félix
S. do Cristalino
S. Arqueada
S. da Onça
Tucumã
Água Azul do Norte
Sapucaia
Legend
Municipal headquarters
Protected Areas
Campos Ferruginosos National Park
Carajás National Forest
Rock Outcrops
MCP - Before Field Investigation
MCP - After Field Investigation
Borreria heteranthera
N
W
E
S
10 5 0 10 20
Km
Coordinate System: SIRGAS 2000 UTM Zone 22S
Projection: Transverse Mercator
Datum: SIRGAS 2000

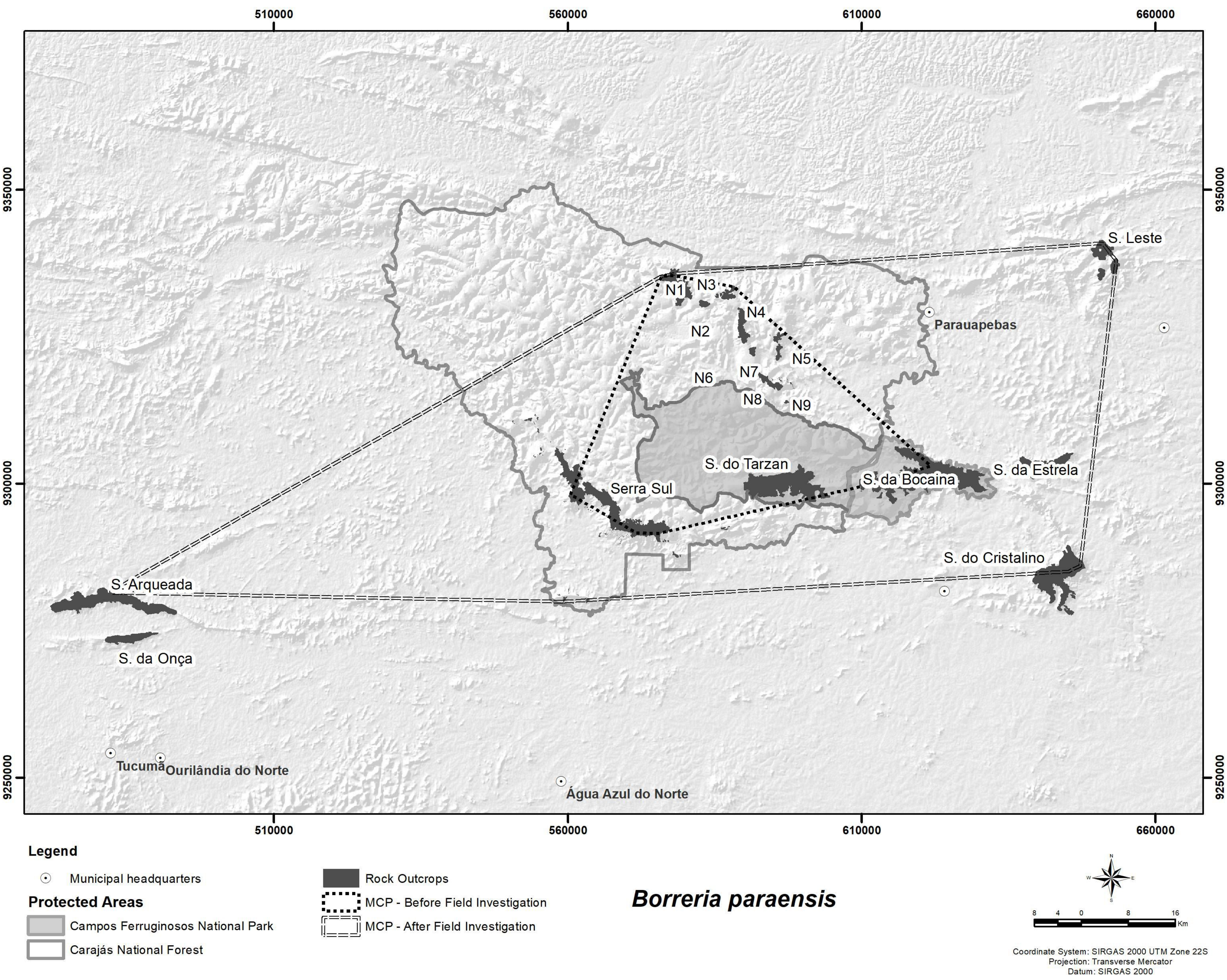
510000
560000
610000
660000
9350000
9300000
9250000
S. Leste
N1
N3
N4
N2
N5
N6
N7
N8
N9
Parauapebas
S. do Tarzan
S. da Bocaina
S. da Estrela
Serra Sul
S. do Cristalino
S. Arqueada
S. da Onça
Tucumã
Ourilândia do Norte
Água Azul do Norte
Legend
Municipal headquarters
Rock Outcrops
MCP - Before Field Investigation
MCP - After Field Investigation
Protected Areas
Campos Ferruginosos National Park
Carajás National Forest
Borreria paraensis
8 4 0 8 16
Km
Coordinate System: SIRGAS 2000 UTM Zone 22S
Projection: Transverse Mercator
Datum: SIRGAS 2000

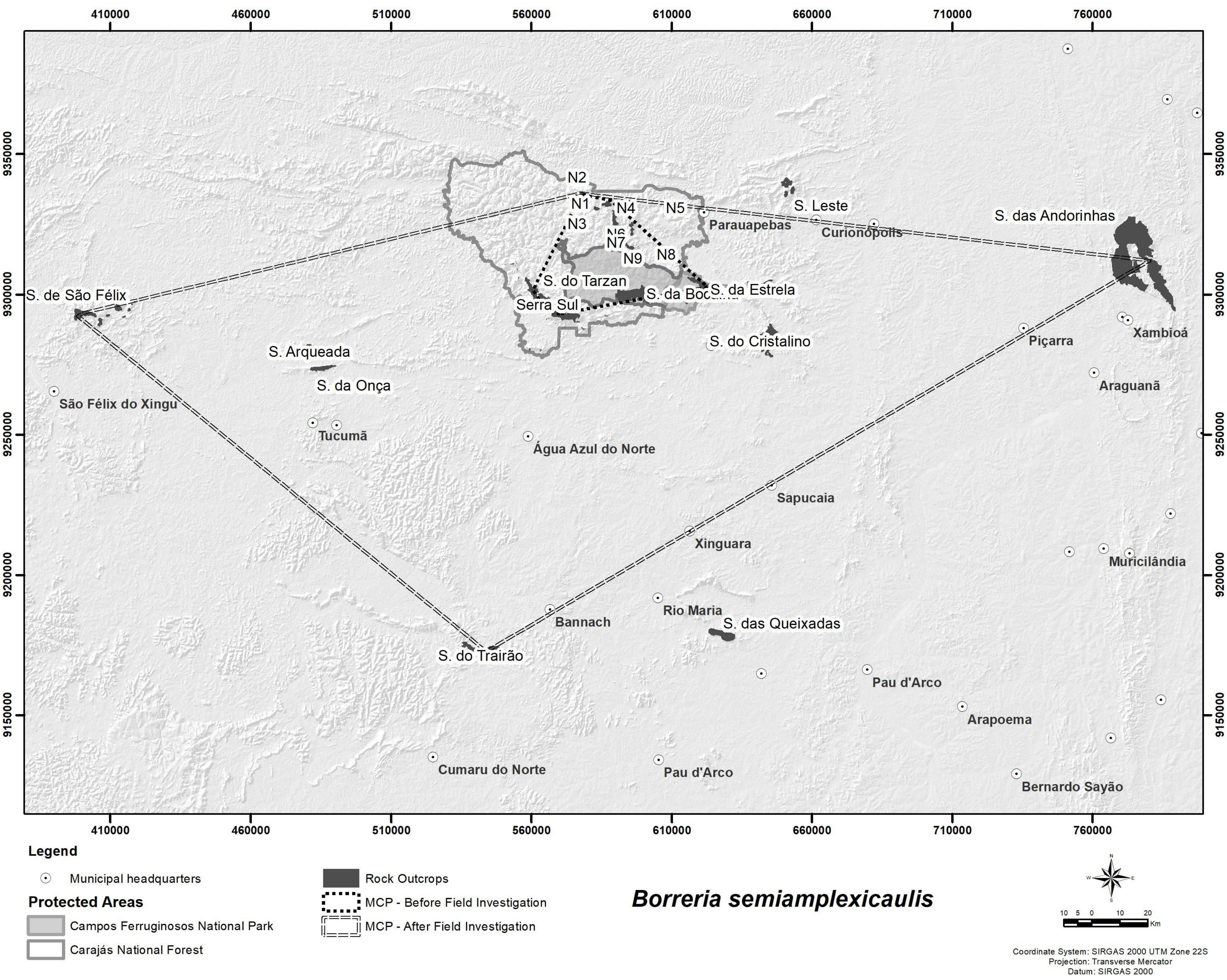
410000
460000
510000
560000
610000
660000
710000
760000
9350000
9300000
9250000
9200000
9150000
N2
N1
N4
N5
N3
N7
N9
N8
S. Leste
Parauapebas
Curionópolis
S. das Andorinhas
S. de São Félix
S. do Tarzan
Serra Sul
S. da Estrela
S. do Cristalino
Xambioá
Piçarra
S. Arqueada
S. da Onça
São Félix do Xingu
Araguanã
Tucumã
Água Azul do Norte
Sapucaia
Xinguara
Muricilândia
Rio Maria
Bannach
S. das Queixadas
S. do Trairão
Pau d'Arco
Arapoema
Cumaru do Norte
Pau d'Arco
Bernardo Sayão
Legend
Municipal headquarters
Protected Areas
Campos Ferruginosos National Park
Carajás National Forest
Rock Outcrops
MCP - Before Field Investigation
MCP - After Field Investigation
Borreria semiamplexicaulis
10 5 0 10 20
Km
Coordinate System: SIRGAS 2000 UTM Zone 22S
Projection: Transverse Mercator
Datum: SIRGAS 2000

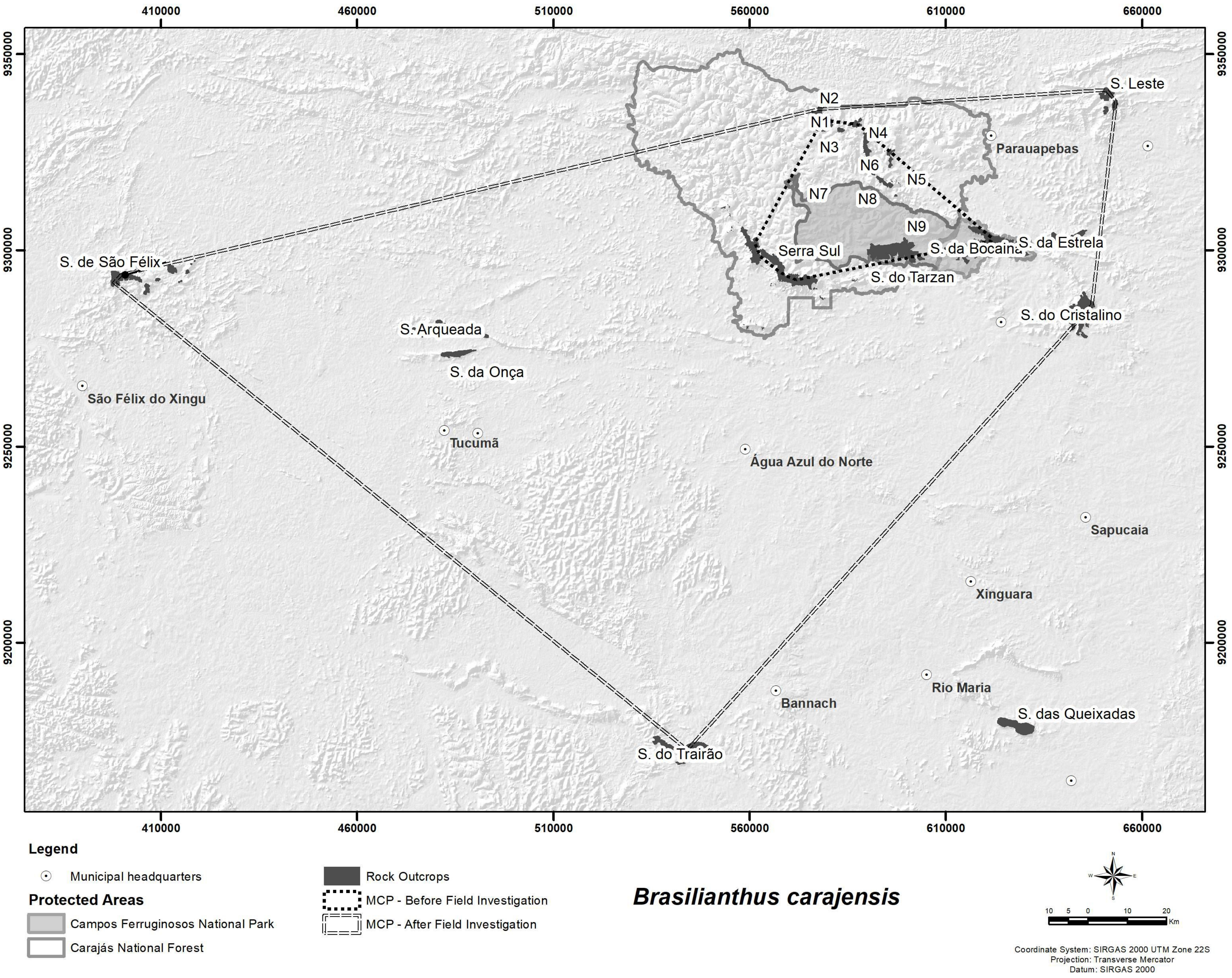
410000
460000
510000
560000
610000
660000
9350000
9300000
9250000
9200000
N2
N1
N3
N4
N6
N5
N7
N8
N9
S. Leste
Parauapebas
Serra Sul
S. da Bocaina
S. da Estrela
S. do Tarzan
S. de São Félix
S. do Cristalino
S. Arqueada
S. da Onça
São Félix do Xingu
Tucumã
Água Azul do Norte
Sapucaia
Xinguara
Rio Maria
Bannach
S. das Queixadas
S. do Trairão
Legend
Municipal headquarters
Protected Areas
Campos Ferruginosos National Park
Carajás National Forest
Rock Outcrops
MCP - Before Field Investigation
MCP - After Field Investigation
Brasilianthus carajensis
10 5 0 10 20
Km
Coordinate System: SIRGAS 2000 UTM Zone 22S
Projection: Transverse Mercator
Datum: SIRGAS 2000

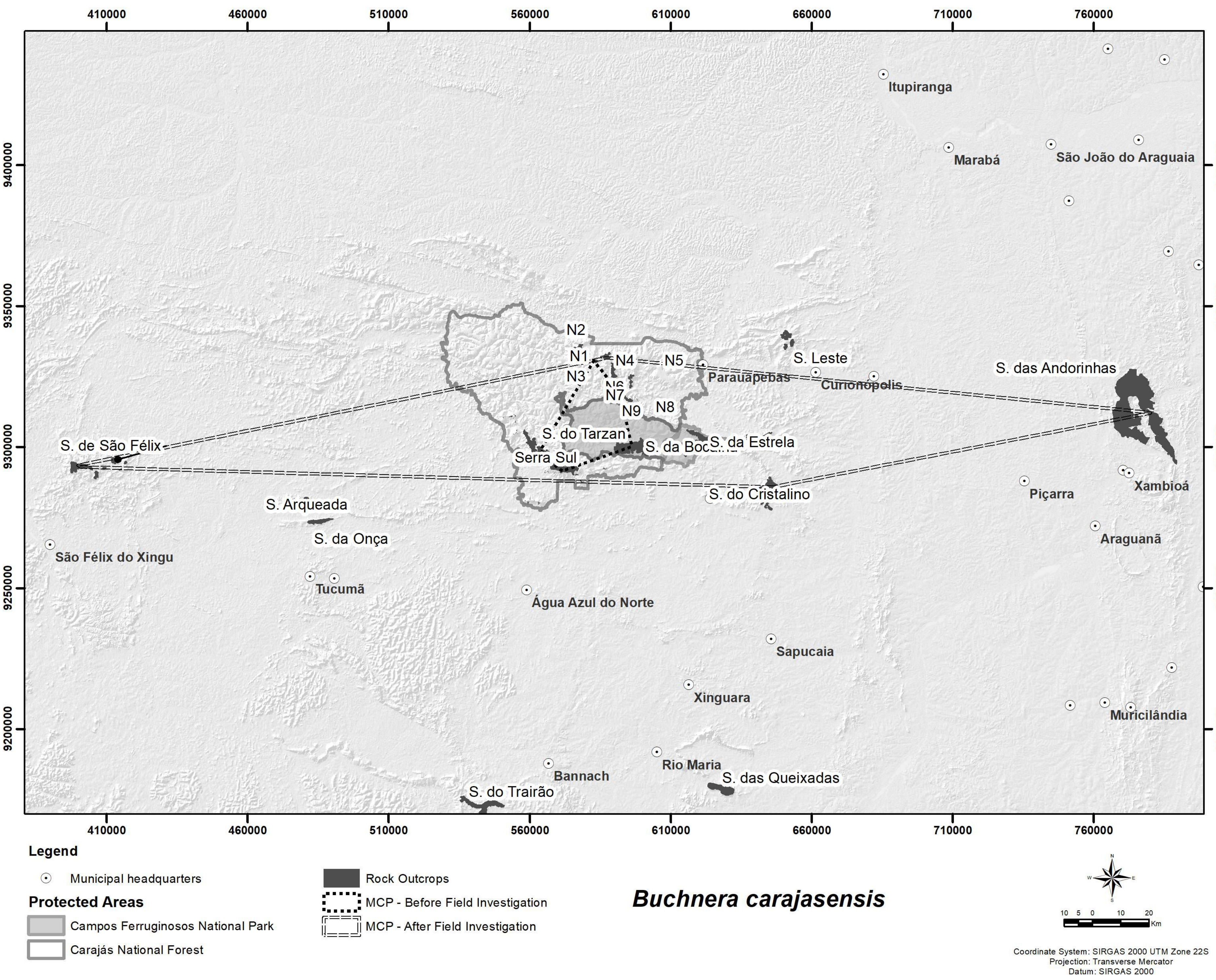

Itupiranga
Marabá
São João do Araguaia
N2
N1
N3
N4
N5
N7
N9
N8
S. Leste
Parauapebas
S. das Andorinhas
S. do Tarzan
S. da Estrela
Serra Sul
S. de São Félix
S. do Cristalino
Piçarra
Xambioá
S. Arqueada
S. da Onça
Araguanã
São Félix do Xingu
Tucumã
Água Azul do Norte
Sapucaia
Xinguara
Muricilândia
Bannach
Rio Maria
S. das Queixadas
S. do Trairão
Legend
Municipal headquarters
Protected Areas
Campos Ferruginosos National Park
Carajás National Forest
Rock Outcrops
MCP - Before Field Investigation
MCP - After Field Investigation
Buchnera carajasensis
Coordinate System: SIRGAS 2000 UTM Zone 22S
Projection: Transverse Mercator
Datum: SIRGAS 2000

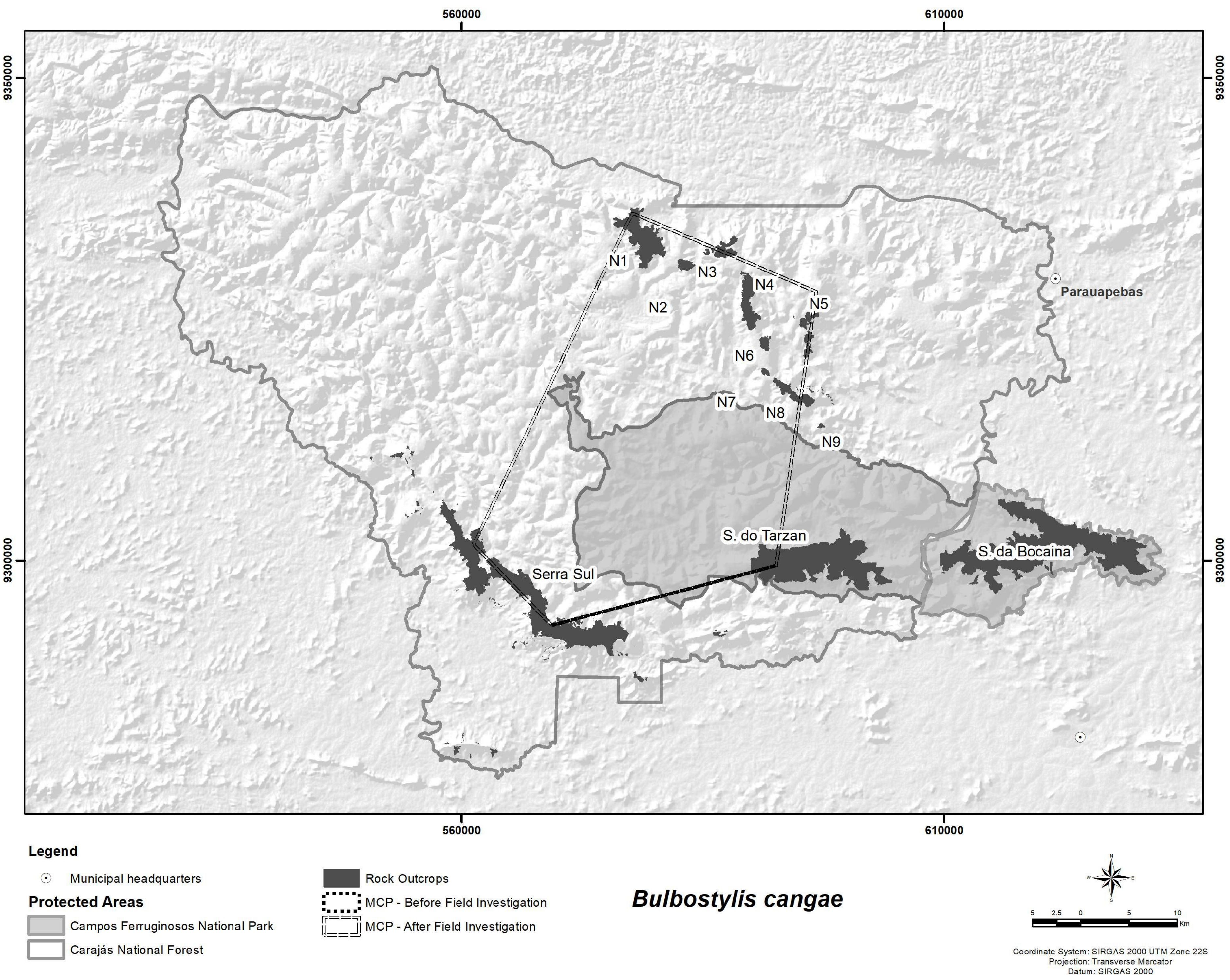
560000
610000
9350000
9300000
N1
N2
N3
N4
N5
N6
N7
N8
N9
Parauapebas
S. do Tarzan
S. da Bocaina
Serra Sul
Legend
Municipal headquarters
Protected Areas
Campos Ferruginosos National Park
Carajás National Forest
Rock Outcrops
MCP - Before Field Investigation
MCP - After Field Investigation
Bulbostylis cangae
5 2.5 0 5 10
Km
Coordinate System: SIRGAS 2000 UTM Zone 22S
Projection: Transverse Mercator
Datum: SIRGAS 2000

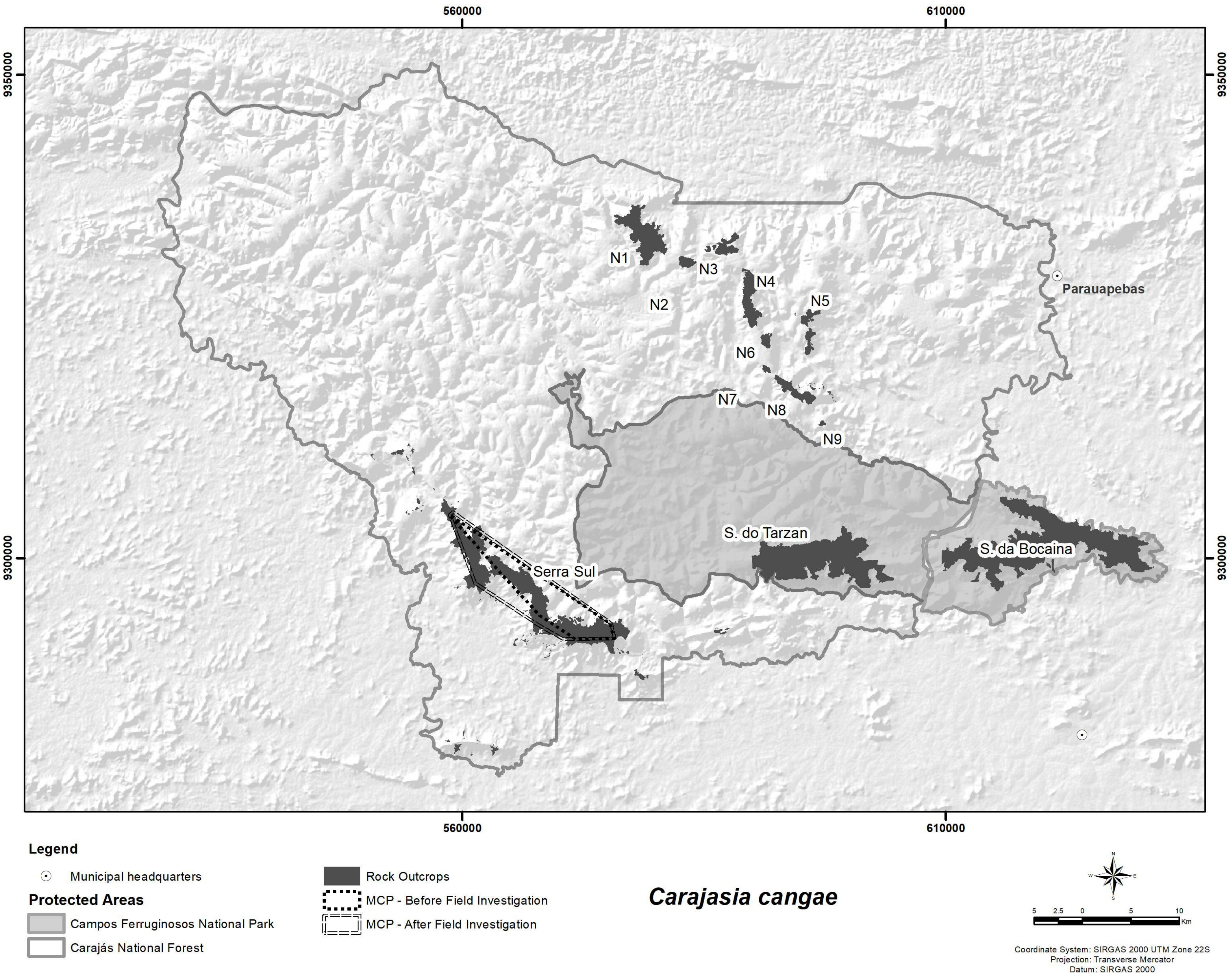
560000
610000
9350000
9300000
N1
N2
N3
N4
N5
N6
N7
N8
N9
Parauapebas
S. do Tarzan
S. da Bocaina
Serra Sul
Legend
Municipal headquarters
Protected Areas
Campos Ferruginosos National Park
Carajás National Forest
Rock Outcrops
MCP - Before Field Investigation
MCP - After Field Investigation
Carajasia cangae
5 2.5 0 5 10
Km
Coordinate System: SIRGAS 2000 UTM Zone 22S
Projection: Transverse Mercator
Datum: SIRGAS 2000

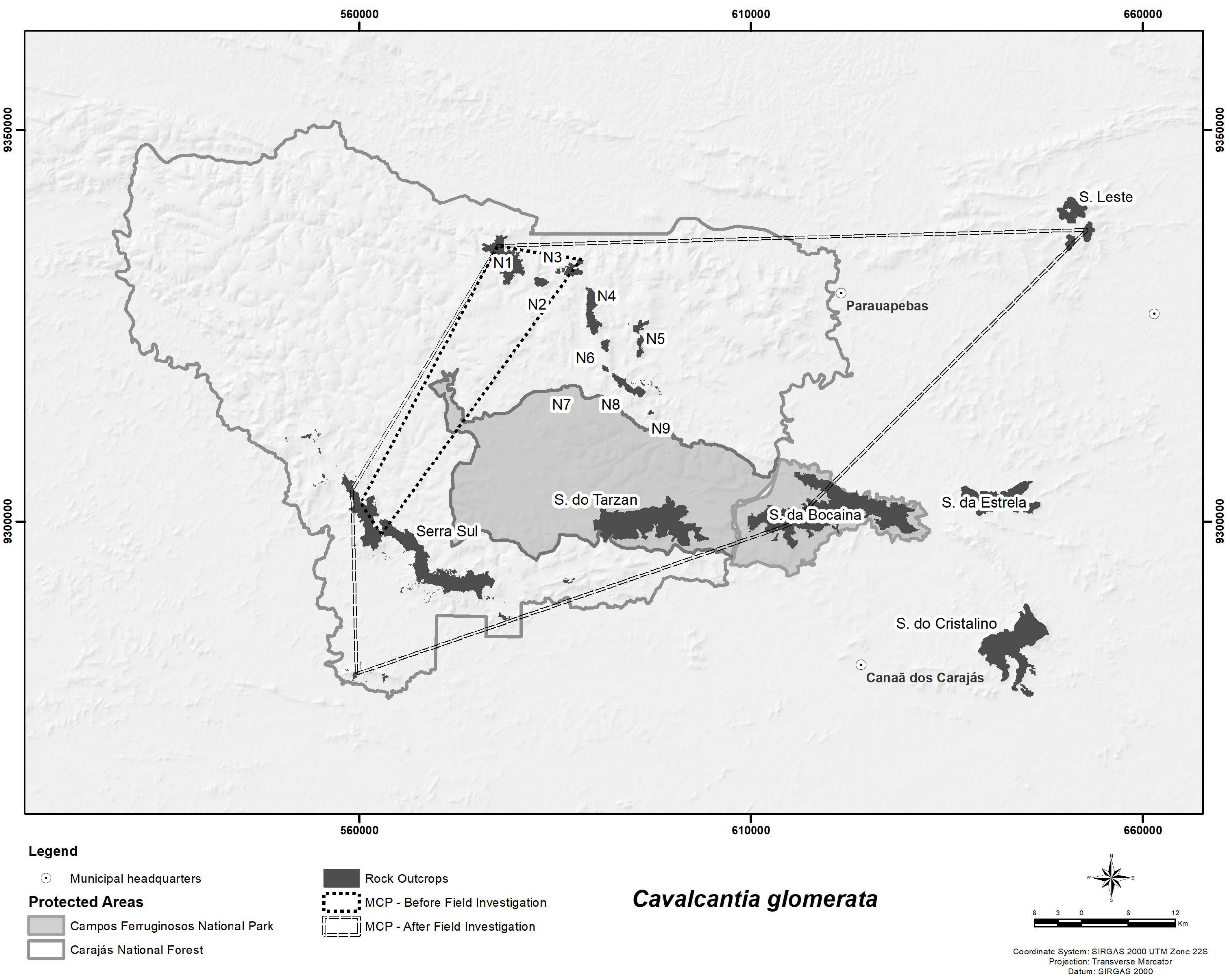

560000
610000
660000
9350000
9300000
S. Leste
N1
N3
N2
N4
N5
N6
N7
N8
N9
Parauapebas
S. do Tarzan
S. da Bocaina
S. da Estrela
Serra Sul
S. do Cristalino
Canaã dos Carajás
Legend
Municipal headquarters
Protected Areas
Campos Ferruginosos National Park
Carajás National Forest
Rock Outcrops
MCP - Before Field Investigation
MCP - After Field Investigation
Cavalcantia glomerata
6 3 0 6 12
Km
Coordinate System: SIRGAS 2000 UTM Zone 22S
Projection: Transverse Mercator
Datum: SIRGAS 2000

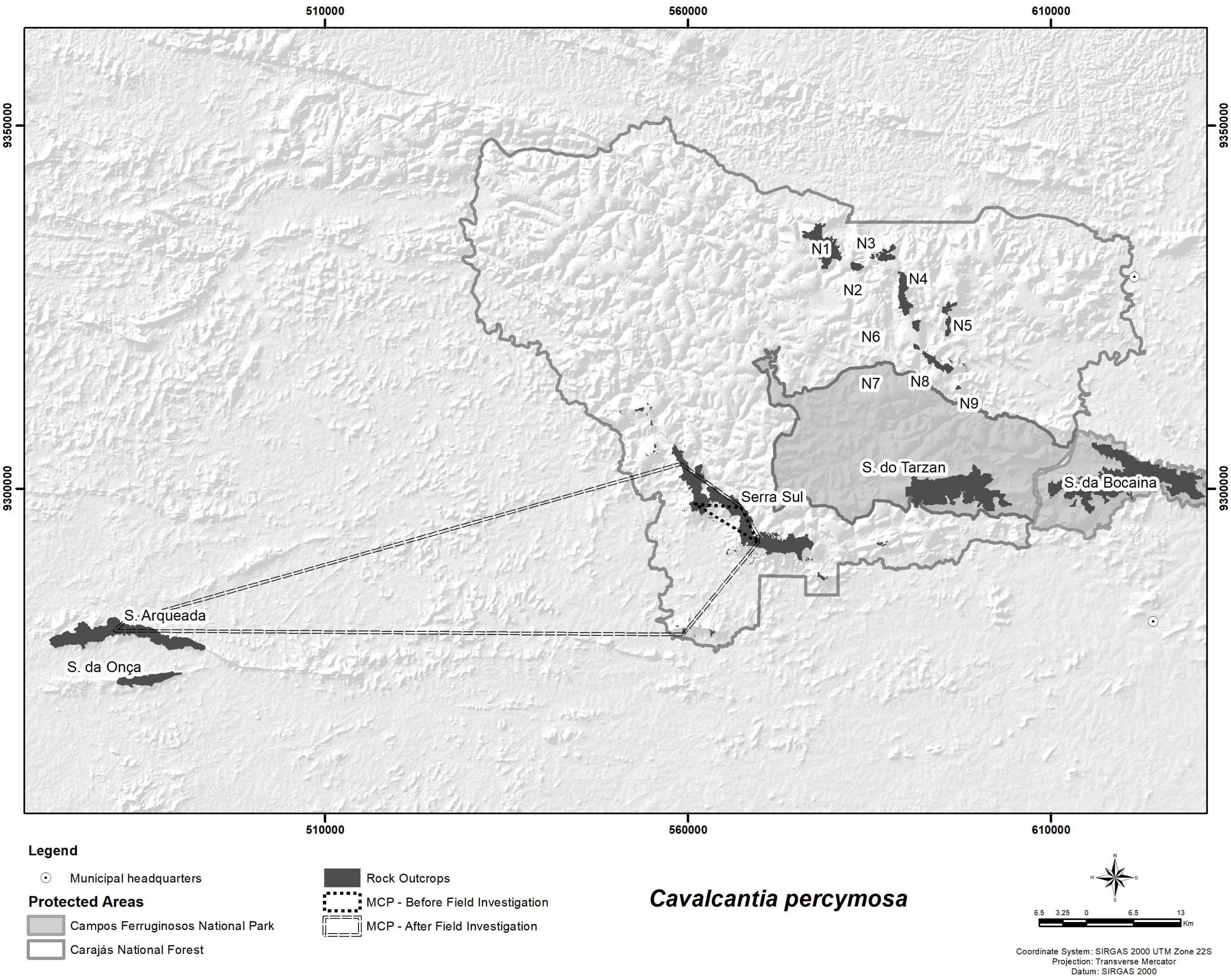
510000
560000
610000
9350000
9300000
N1
N2
N3
N4
N5
N6
N7
N8
N9
S. do Tarzan
S. da Bocaina
Serra Sul
S. Arqueada
S. da Onça
Legend
Municipal headquarters
Protected Areas
Campos Ferruginosos National Park
Carajás National Forest
Rock Outcrops
MCP - Before Field Investigation
MCP - After Field Investigation
Cavalcantia percymosa
6.5 3.25 0 6.5 13
Km
Coordinate System: SIRGAS 2000 UTM Zone 22S
Projection: Transverse Mercator
Datum: SIRGAS 2000

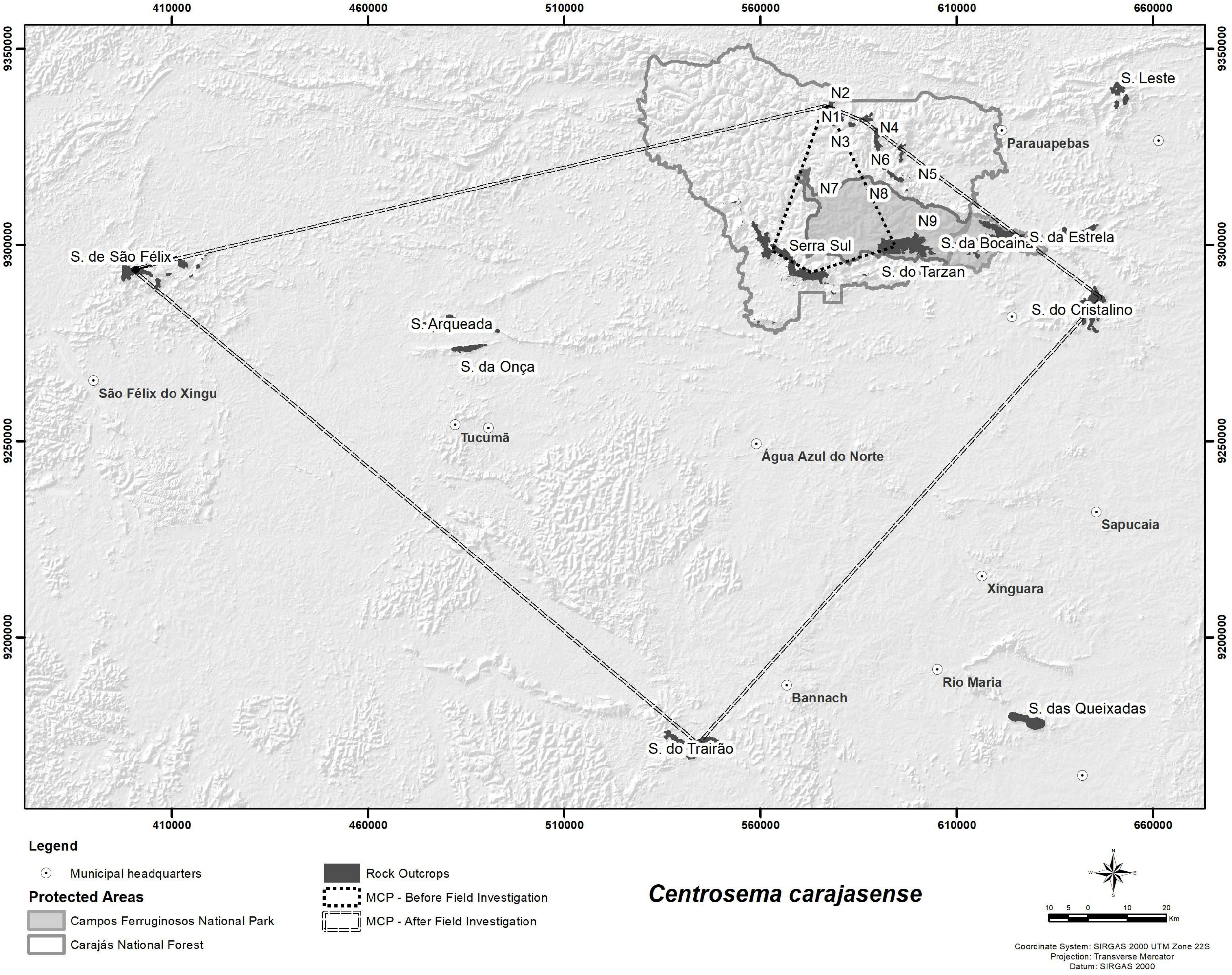

410000
460000
510000
560000
610000
660000
9350000
9300000
9250000
9200000
S. Leste
N2
N1
N4
N3
N6
N5
N7
N8
N9
Parauapebas
S. de São Félix
Serra Sul
S. da Bocaina
S. da Estrela
S. do Tarzan
S. do Cristalino
S. Arqueada
S. da Onça
São Félix do Xingu
Tucumã
Água Azul do Norte
Sapucaia
Xinguara
Rio Maria
Bannach
S. das Queixadas
S. do Trairão
Legend
Municipal headquarters
Protected Areas
Campos Ferruginosos National Park
Carajás National Forest
Rock Outcrops
MCP - Before Field Investigation
MCP - After Field Investigation
Centrosema carajasense
N
W
E
S
10 5 0 10 20
Km
Coordinate System: SIRGAS 2000 UTM Zone 22S
Projection: Transverse Mercator
Datum: SIRGAS 2000

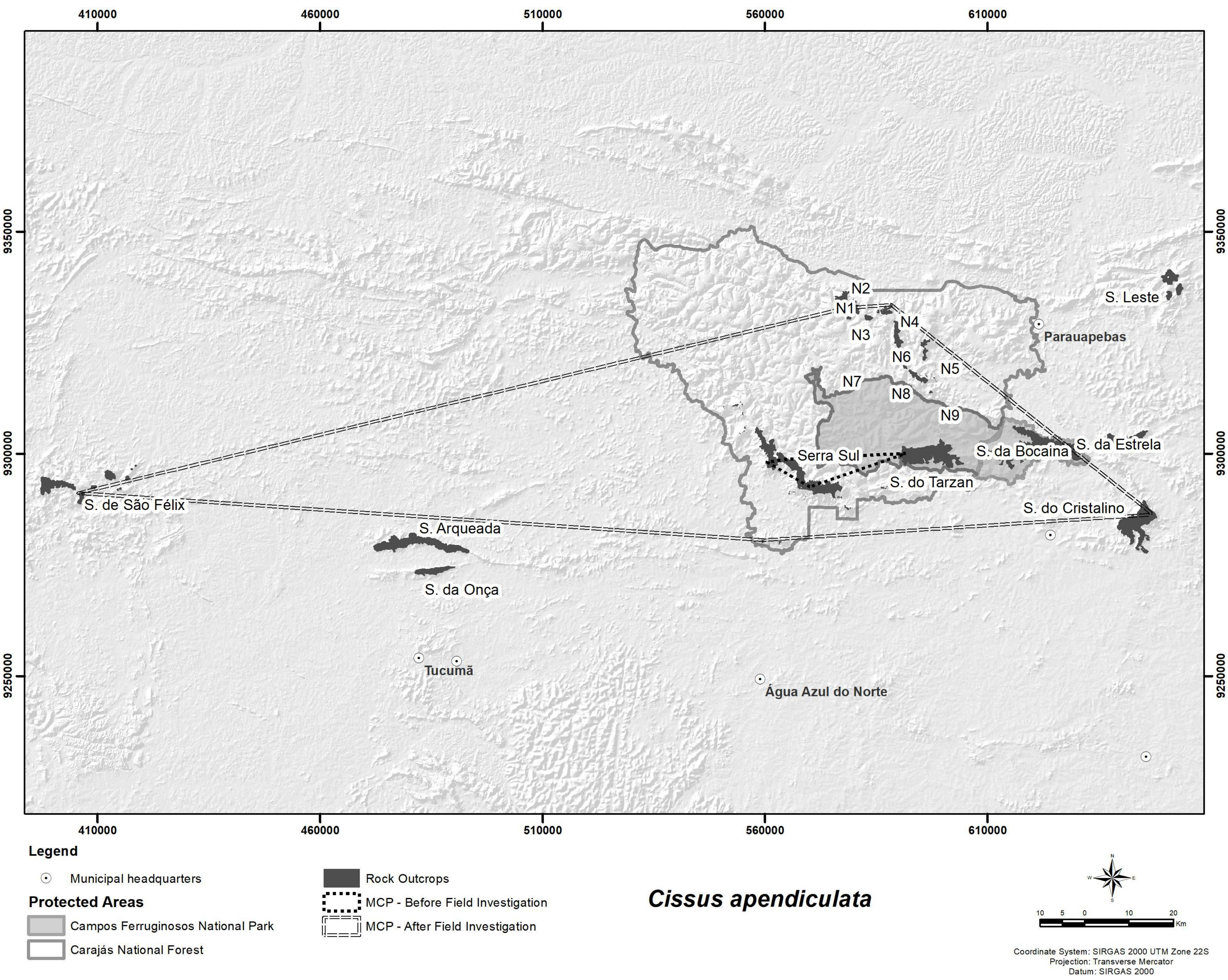

410000
460000
510000
560000
610000
9350000
9300000
9250000
N1
N2
N3
N4
N5
N6
N7
N8
N9
S. Leste
Parauapebas
Serra Sul
S. da Bocaina
S. da Estrela
S. do Tarzan
S. do Cristalino
S. de São Félix
S. Arqueada
S. da Onça
Tucumã
Água Azul do Norte
Legend
Municipal headquarters
Protected Areas
Campos Ferruginosos National Park
Carajás National Forest
Rock Outcrops
MCP - Before Field Investigation
MCP - After Field Investigation
Cissus apendiculata
10 5 0 10 20
Km
Coordinate System: SIRGAS 2000 UTM Zone 22S
Projection: Transverse Mercator
Datum: SIRGAS 2000

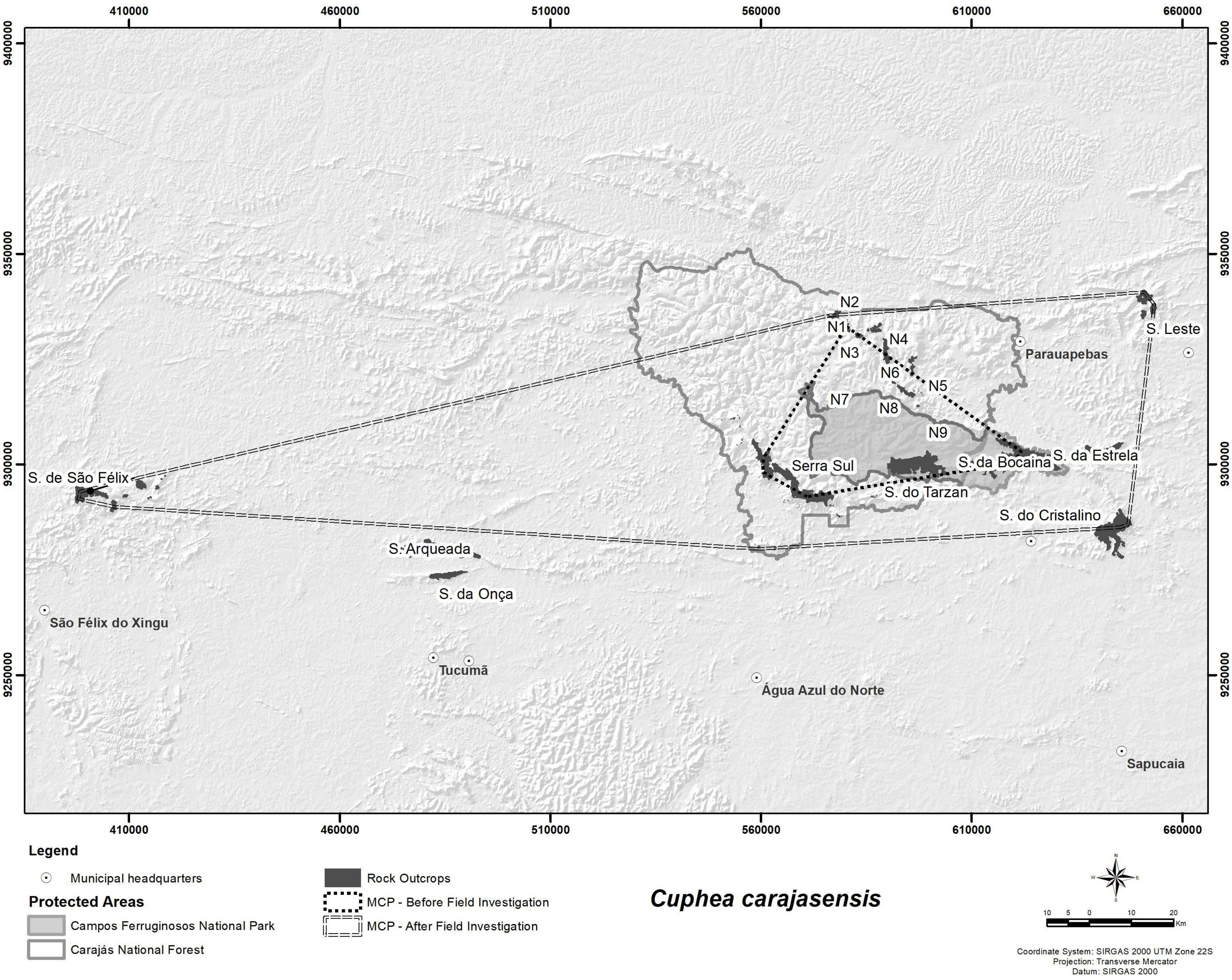
410000
460000
510000
560000
610000
660000
9400000
9350000
9300000
9250000
N2
N1
N3
N4
N6
N5
N7
N8
N9
S. Leste
Parauapebas
S. de São Félix
Serra Sul
S. da Bocaina
S. da Estrela
S. do Tarzan
S. do Cristalino
S. Arqueada
S. da Onça
São Félix do Xingu
Tucumã
Água Azul do Norte
Sapucaia
Legend
Municipal headquarters
Protected Areas
Campos Ferruginosos National Park
Carajás National Forest
Rock Outcrops
MCP - Before Field Investigation
MCP - After Field Investigation
Cuphea carajasensis
10 5 0 10 20
Km
Coordinate System: SIRGAS 2000 UTM Zone 22S
Projection: Transverse Mercator
Datum: SIRGAS 2000

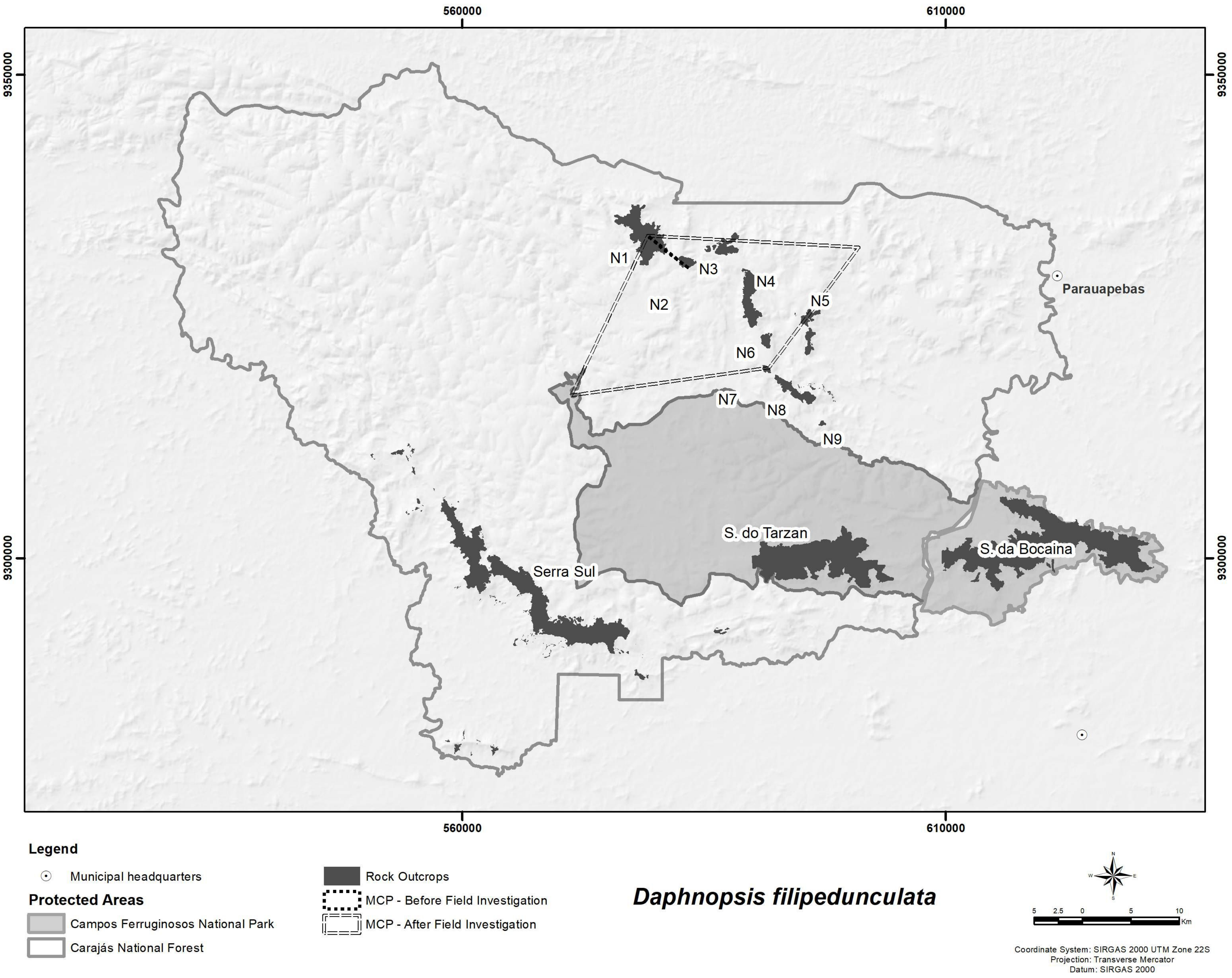
560000
610000
9350000
9300000
N1
N2
N3
N4
N5
N6
N7
N8
N9
Parauapebas
S. do Tarzan
S. da Bocaina
Serra Sul
Legend
Municipal headquarters
Protected Areas
Campos Ferruginosos National Park
Carajás National Forest
Rock Outcrops
MCP - Before Field Investigation
MCP - After Field Investigation
Daphnopsis filipedunculata
N
W
E
S
5 2.5 0 5 10
Km
Coordinate System: SIRGAS 2000 UTM Zone 22S
Projection: Transverse Mercator
Datum: SIRGAS 2000

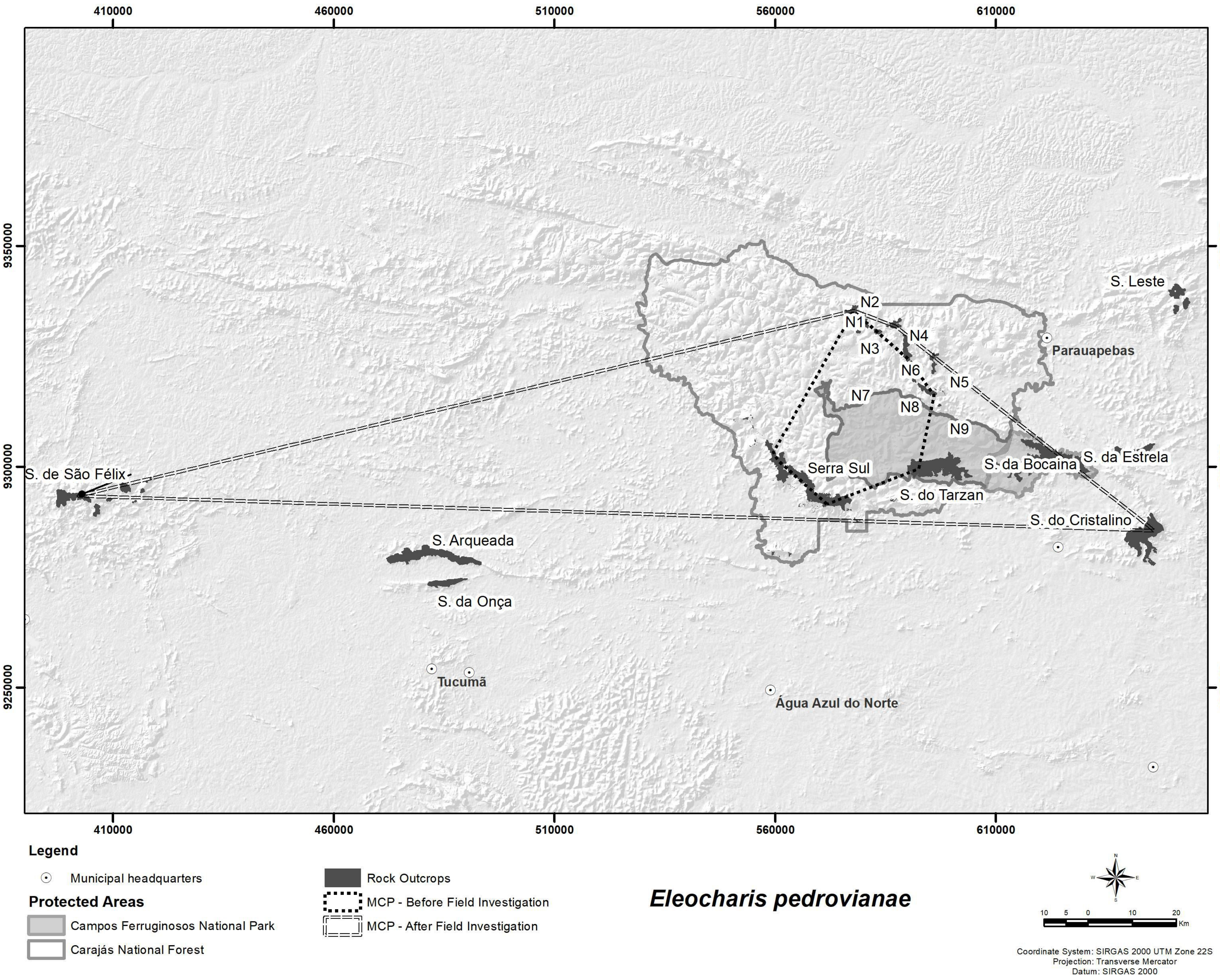
410000
460000
510000
560000
610000
9350000
9300000
9250000
S. Leste
N2
N1
N4
N3
Parauapebas
N6
N5
N7
N8
N9
Serra Sul
S. da Bocaina
S. da Estrela
S. de São Félix
S. do Tarzan
S. do Cristalino
S. Arqueada
S. da Onça
Tucumã
Água Azul do Norte
Legend
Municipal headquarters
Protected Areas
Campos Ferruginosos National Park
Carajás National Forest
Rock Outcrops
MCP - Before Field Investigation
MCP - After Field Investigation
Eleocharis pedrovianae
N
W
E
S
10 5 0 10 20
Km
Coordinate System: SIRGAS 2000 UTM Zone 22S
Projection: Transverse Mercator
Datum: SIRGAS 2000

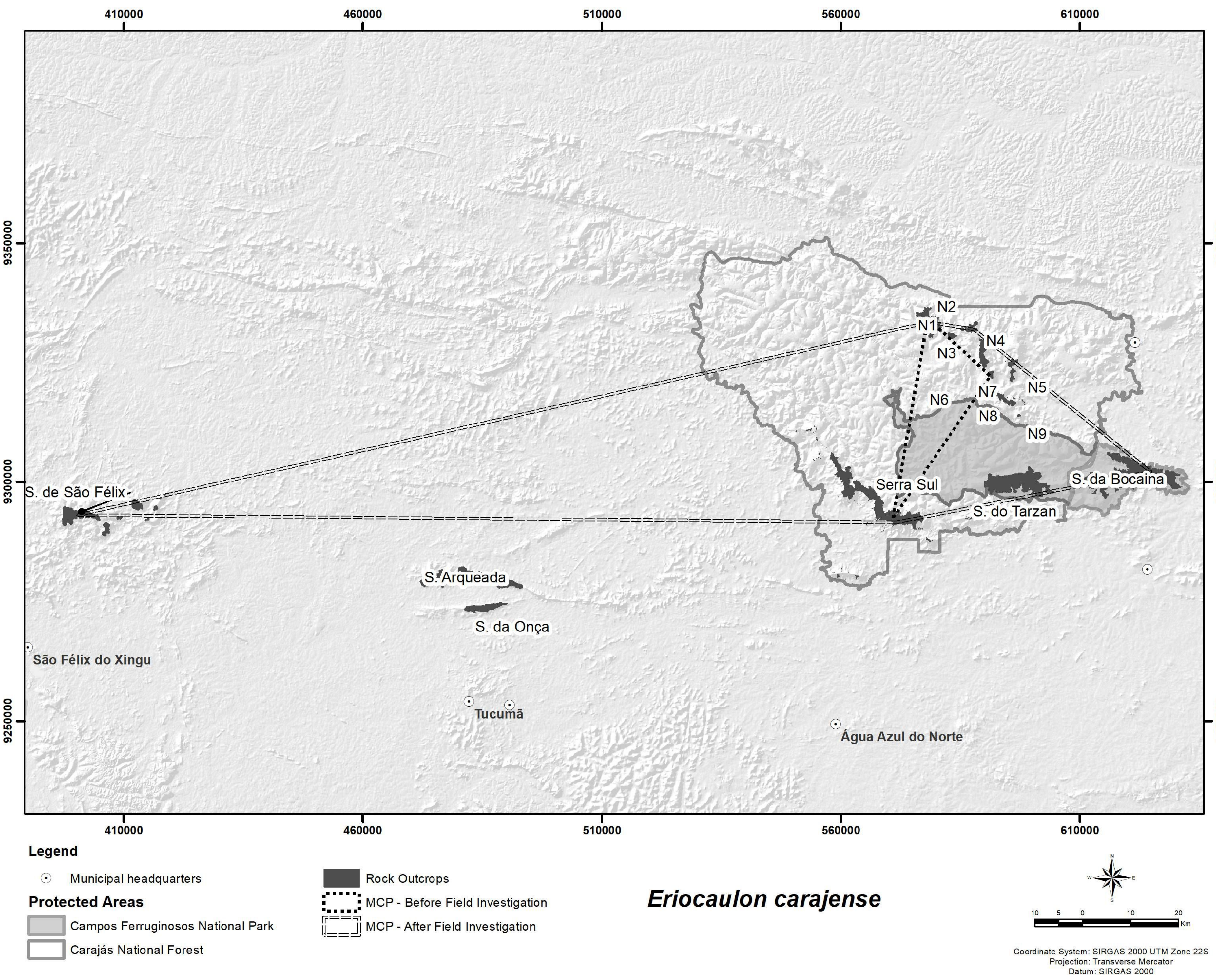
410000
460000
510000
560000
610000
9350000
9300000
9250000
S. de São Félix
N2
N1
N4
N3
N5
N7
N6
N8
N9
Serra Sul
S. da Bocaina
S. do Tarzan
S. Arqueada
S. da Onça
São Félix do Xingu
Tucumã
Água Azul do Norte
Legend
Municipal headquarters
Protected Areas
Campos Ferruginosos National Park
Carajás National Forest
Rock Outcrops
MCP - Before Field Investigation
MCP - After Field Investigation
Eriocaulon carajense
10 5 0 10 20
Km
Coordinate System: SIRGAS 2000 UTM Zone 22S
Projection: Transverse Mercator
Datum: SIRGAS 2000

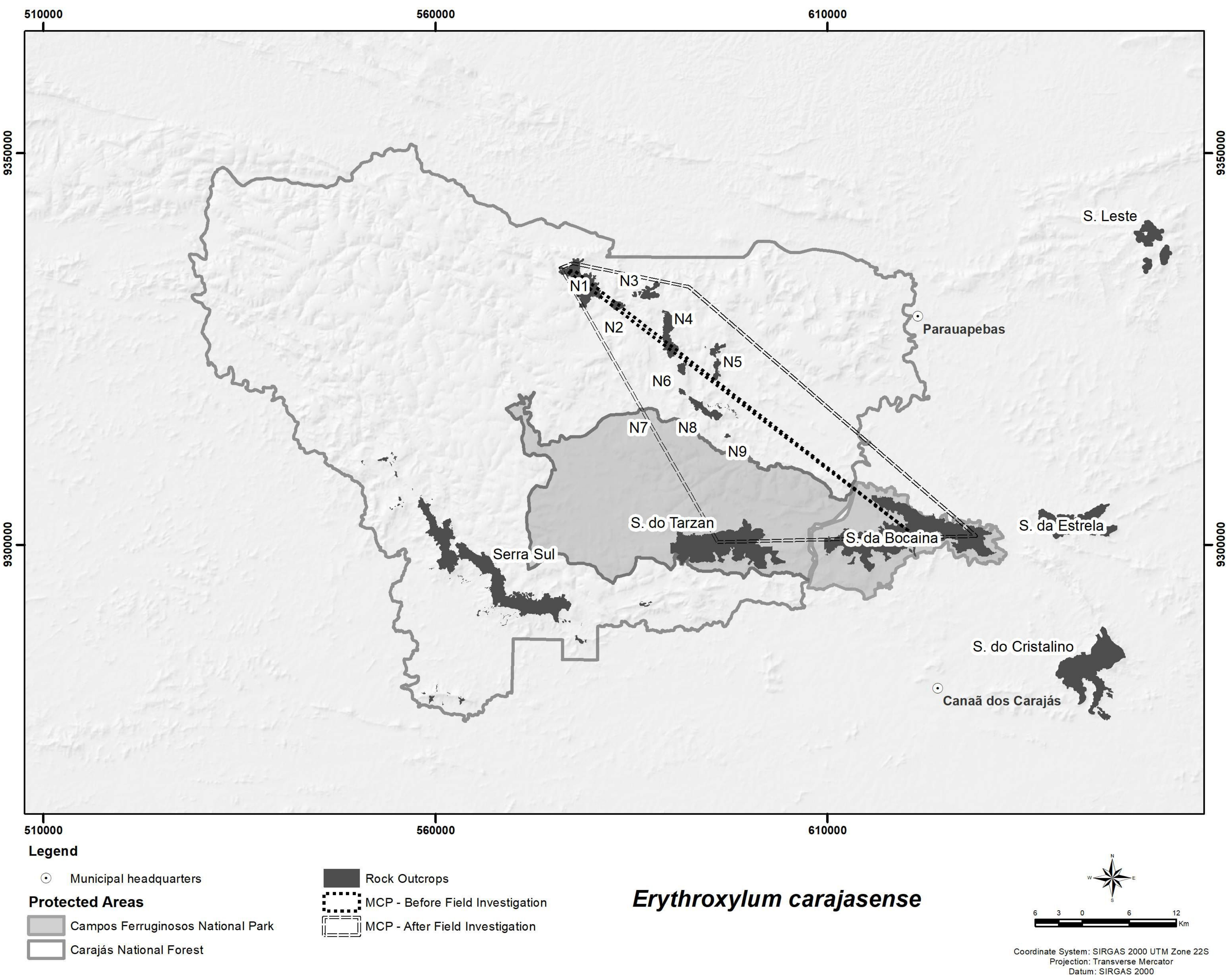
510000
560000
610000
9350000
9300000
S. Leste
N1
N2
N3
N4
N5
N6
N7
N8
N9
Parauapebas
S. do Tarzan
S. da Bocaina
S. da Estrela
Serra Sul
S. do Cristalino
Canaã dos Carajás
Legend
Municipal headquarters
Protected Areas
Campos Ferruginosos National Park
Carajás National Forest
Rock Outcrops
MCP - Before Field Investigation
MCP - After Field Investigation
Erythroxylum carajasense
6 3 0 6 12
Km
Coordinate System: SIRGAS 2000 UTM Zone 22S
Projection: Transverse Mercator
Datum: SIRGAS 2000

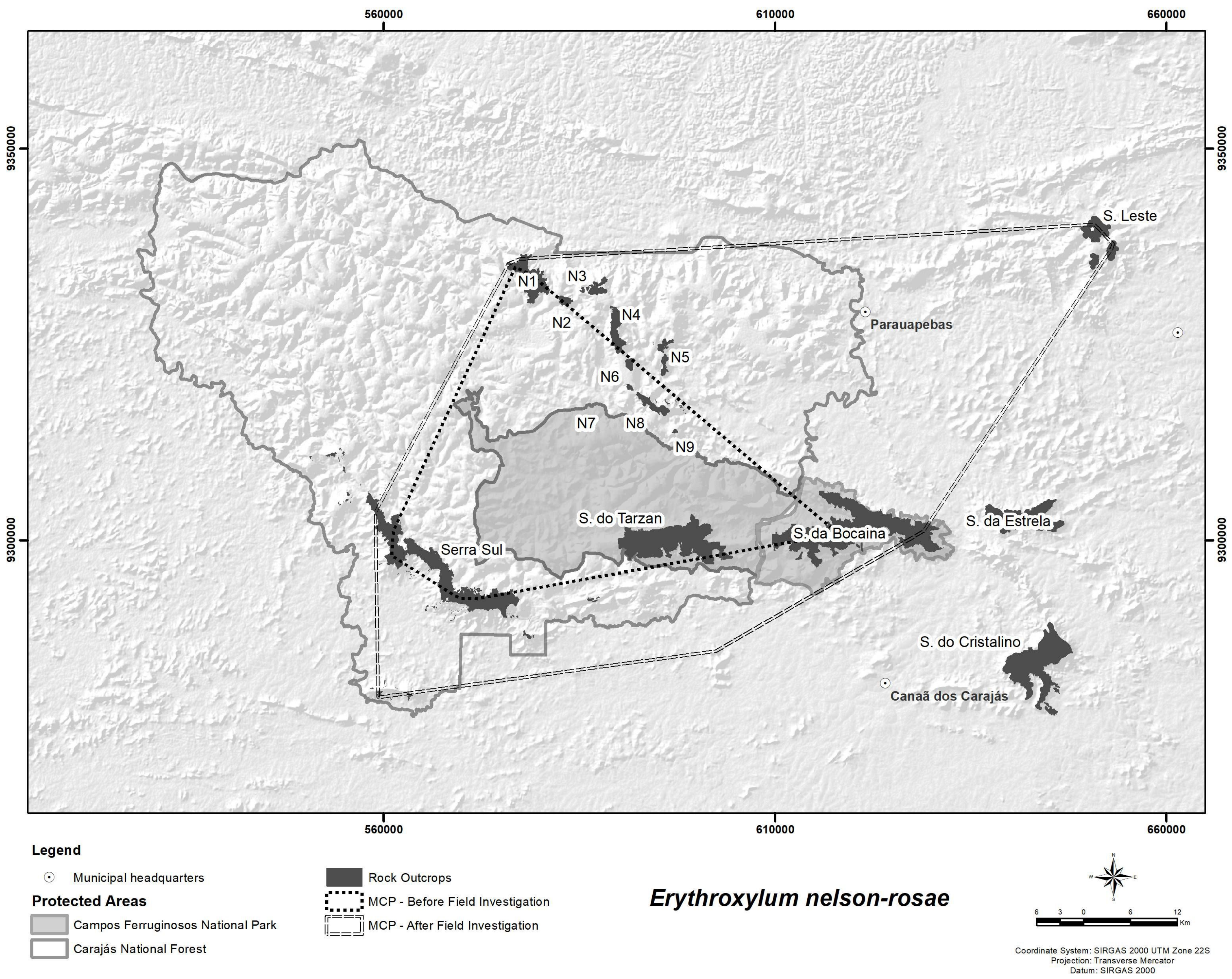
560000
610000
660000
9350000
9300000
S. Leste
N1
N2
N3
N4
N5
N6
N7
N8
N9
Parauapebas
S. do Tarzan
S. da Bocaina
S. da Estrela
Serra Sul
S. do Cristalino
Canaã dos Carajás
Legend
Municipal headquarters
Protected Areas
Campos Ferruginosos National Park
Carajás National Forest
Rock Outcrops
MCP - Before Field Investigation
MCP - After Field Investigation
Erythroxylum nelson-rosae
6 3 0 6 12
Km
Coordinate System: SIRGAS 2000 UTM Zone 22S
Projection: Transverse Mercator
Datum: SIRGAS 2000

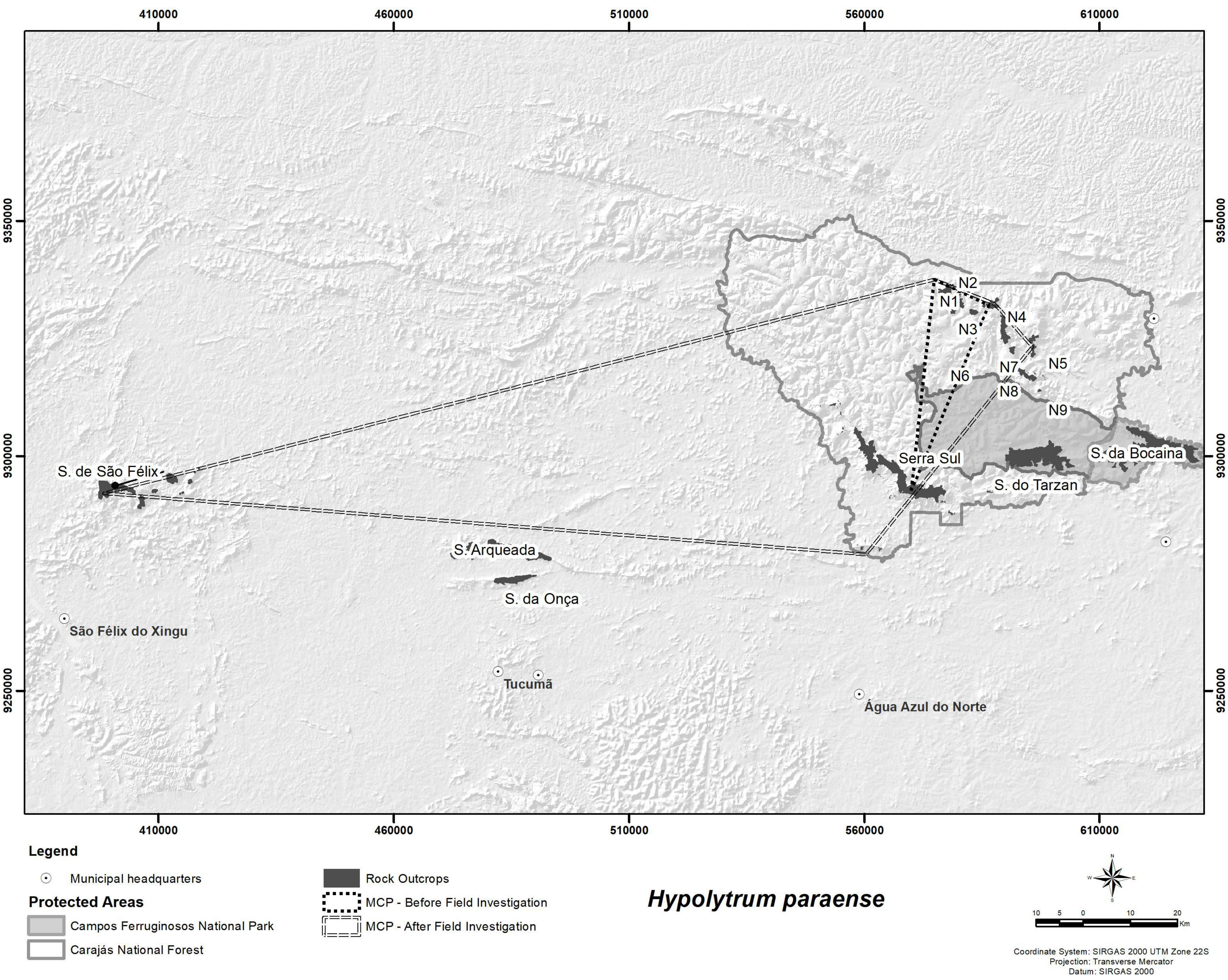
410000
460000
510000
560000
610000
9350000
9300000
9250000
N2
N1
N3
N4
N5
N6
N7
N8
N9
Serra Sul
S. da Bocaina
S. do Tarzan
S. de São Félix
S. Arqueada
S. da Onça
São Félix do Xingu
Tucumã
Água Azul do Norte
Legend
Municipal headquarters
Protected Areas
Campos Ferruginosos National Park
Carajás National Forest
Rock Outcrops
MCP - Before Field Investigation
MCP - After Field Investigation
Hypolytrum paraense
10 5 0 10 20
Km
Coordinate System: SIRGAS 2000 UTM Zone 22S
Projection: Transverse Mercator
Datum: SIRGAS 2000

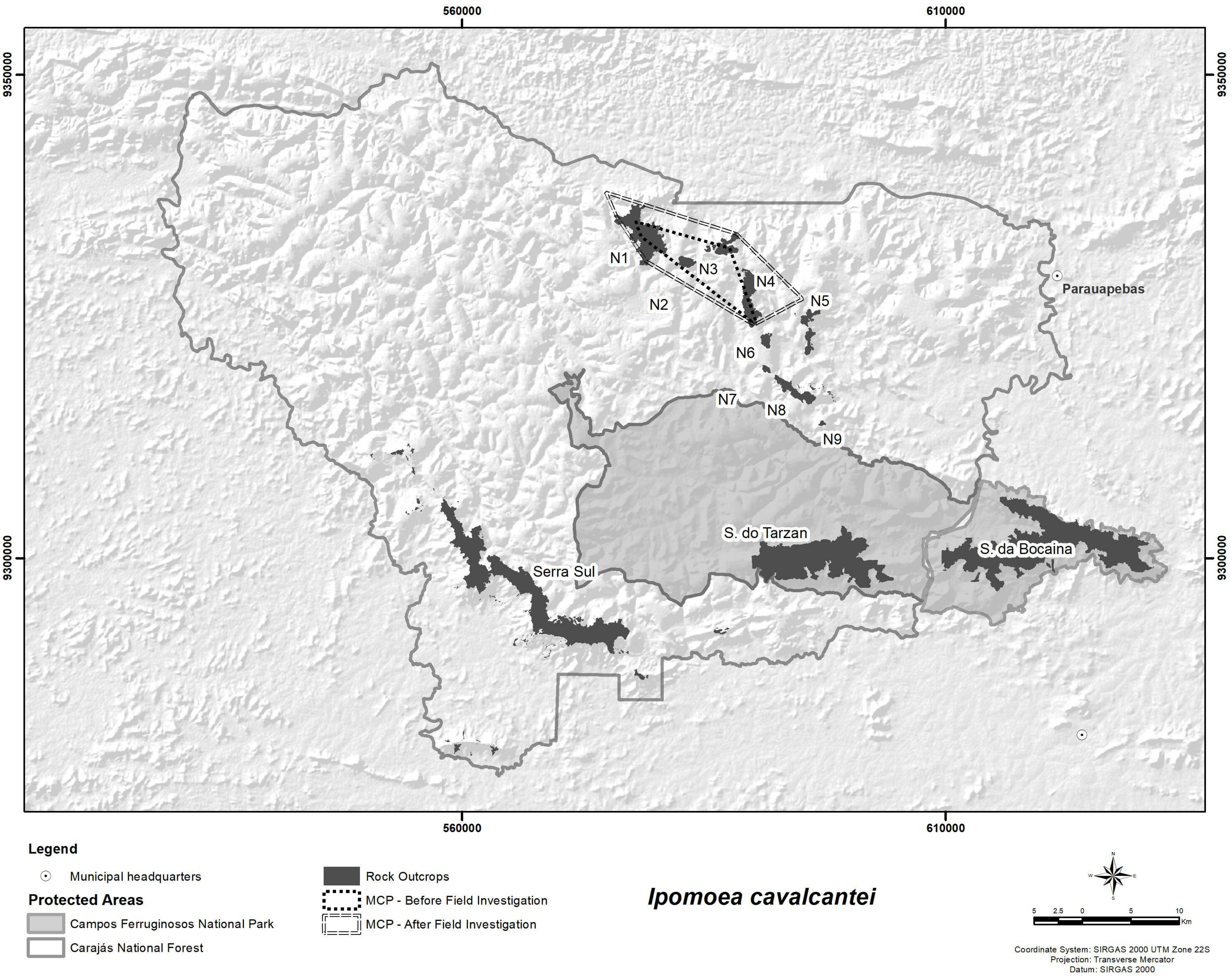
560000
610000
9350000
9300000
N1
N2
N3
N4
N5
N6
N7
N8
N9
Parauapebas
S. do Tarzan
S. da Bocaina
Serra Sul
Legend
Municipal headquarters
Protected Areas
Campos Ferruginosos National Park
Carajás National Forest
Rock Outcrops
MCP - Before Field Investigation
MCP - After Field Investigation
Ipomoea cavalcantei
N
W
E
S
5 2.5 0 5 10
Km
Coordinate System: SIRGAS 2000 UTM Zone 22S
Projection: Transverse Mercator
Datum: SIRGAS 2000

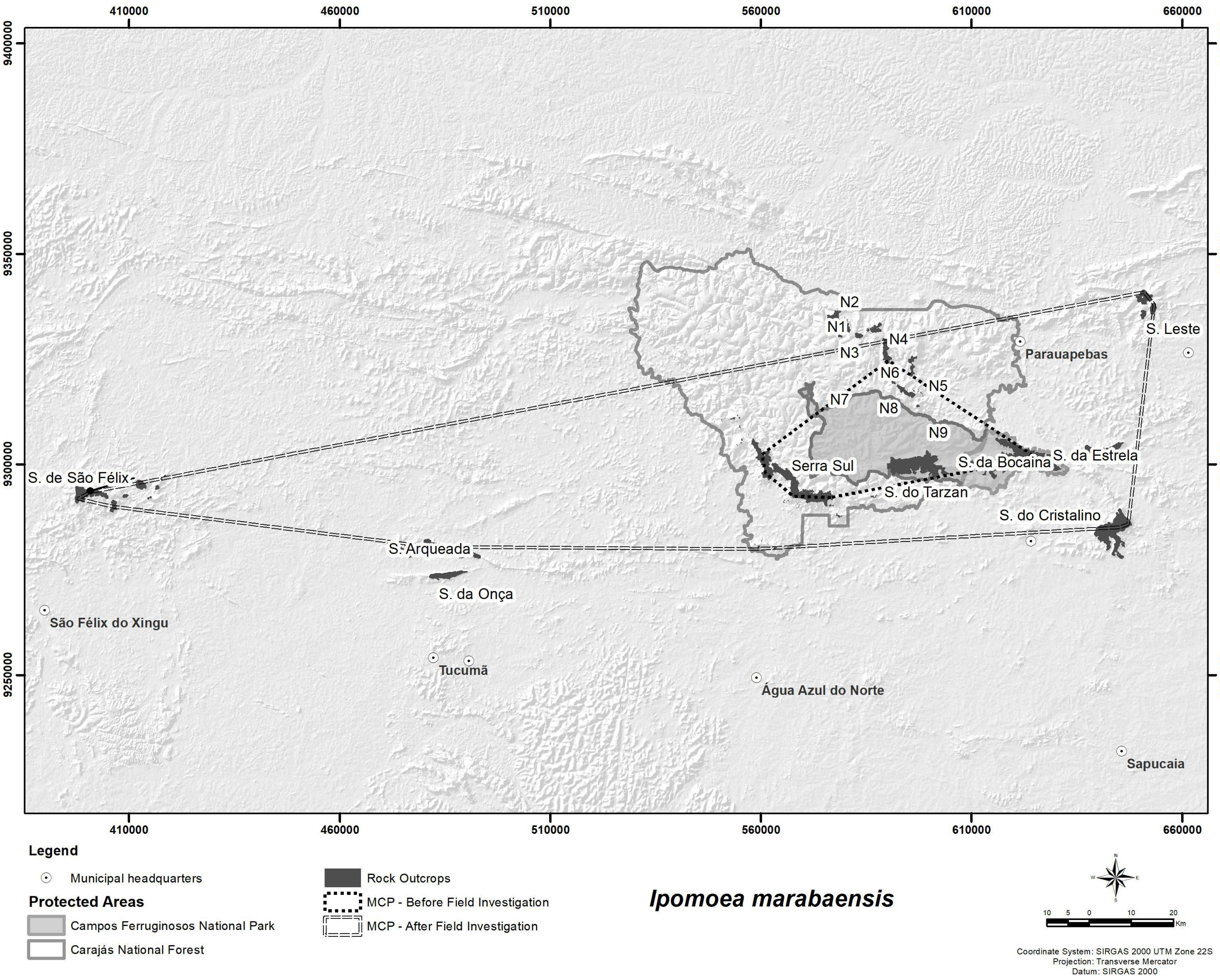
410000
460000
510000
560000
610000
660000
9400000
9350000
9300000
9250000
N2
N1
N4
N3
N6
N5
N7
N8
N9
S. Leste
Parauapebas
S. da Estrela
Serra Sul
S. da Bocaina
S. do Tarzan
S. de São Félix
S. do Cristalino
S. Arqueada
S. da Onça
São Félix do Xingu
Tucumã
Água Azul do Norte
Sapucaia
Legend
Municipal headquarters
Protected Areas
Campos Ferruginosos National Park
Carajás National Forest
Rock Outcrops
MCP - Before Field Investigation
MCP - After Field Investigation
Ipomoea marabaensis
10 5 0 10 20
Km
Coordinate System: SIRGAS 2000 UTM Zone 22S
Projection: Transverse Mercator
Datum: SIRGAS 2000

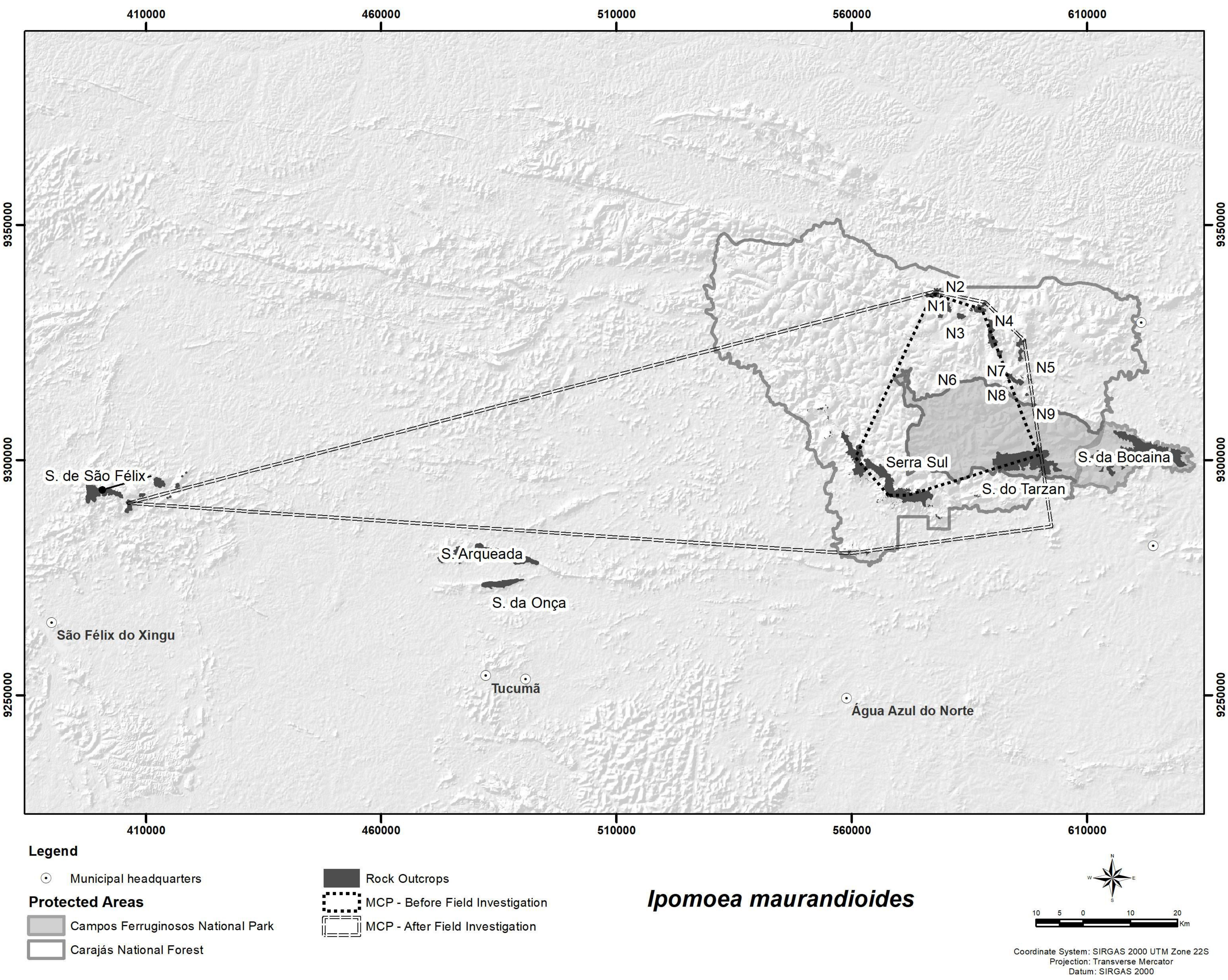
410000
460000
510000
560000
610000
9350000
9300000
9250000
N2
N1
N3
N4
N5
N6
N7
N8
N9
Serra Sul
S. do Tarzan
S. da Bocaina
S. de São Félix
S. Arqueada
S. da Onça
São Félix do Xingu
Tucumã
Água Azul do Norte
Legend
Municipal headquarters
Protected Areas
Campos Ferruginosos National Park
Carajás National Forest
Rock Outcrops
MCP - Before Field Investigation
MCP - After Field Investigation
Ipomoea maurandioides
10 5 0 10 20
Km
Coordinate System: SIRGAS 2000 UTM Zone 22S
Projection: Transverse Mercator
Datum: SIRGAS 2000

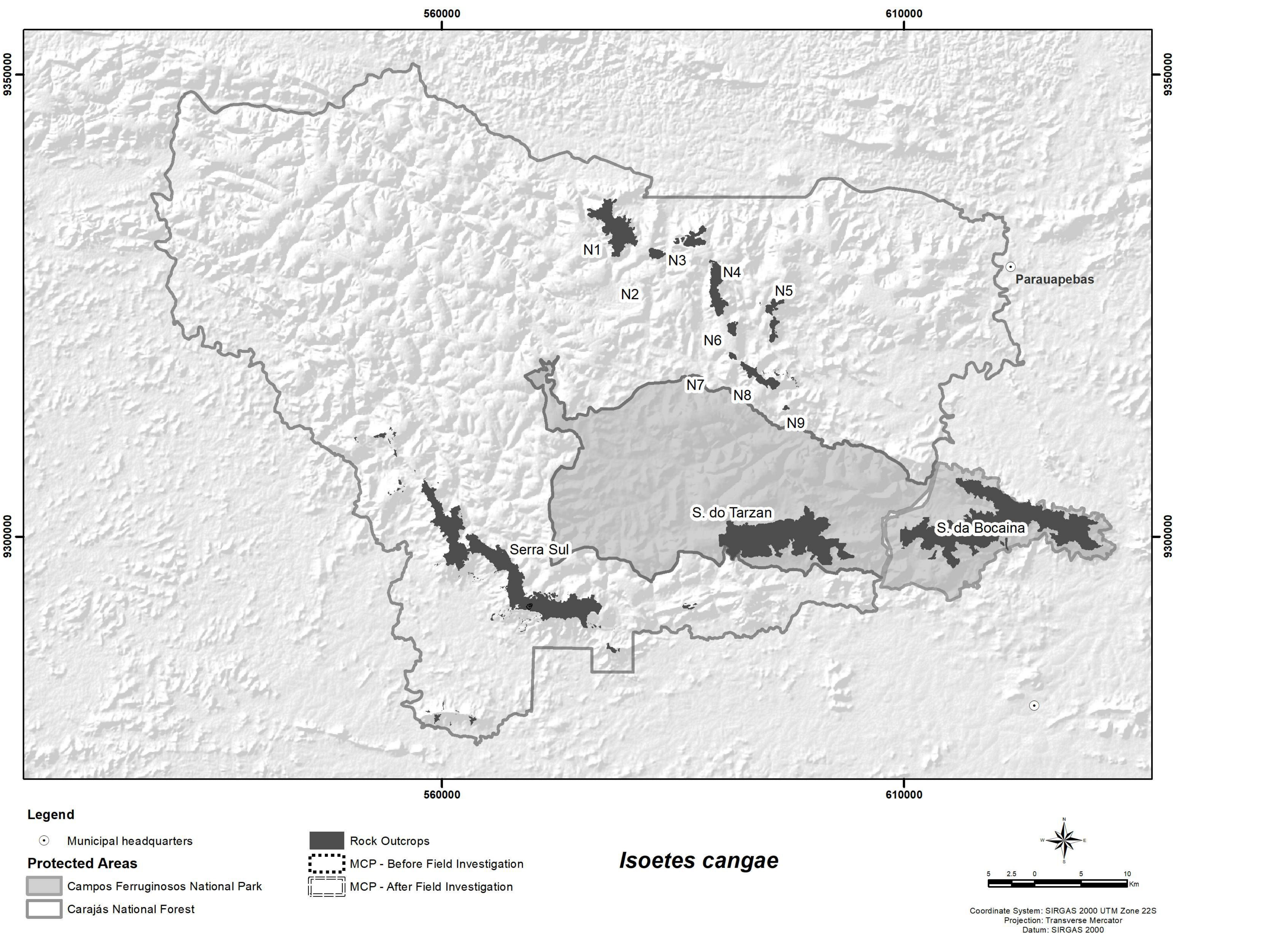
560000
610000
9350000
9300000
N1
N2
N3
N4
N5
N6
N7
N8
N9
Parauapebas
S. do Tarzan
S. da Bocaina
Serra Sul
Legend
Municipal headquarters
Protected Areas
Campos Ferruginosos National Park
Carajás National Forest
Rock Outcrops
MCP - Before Field Investigation
MCP - After Field Investigation
Isoetes cangae
5 2.5 0 5 10
Km
Coordinate System: SIRGAS 2000 UTM Zone 22S
Projection: Transverse Mercator
Datum: SIRGAS 2000

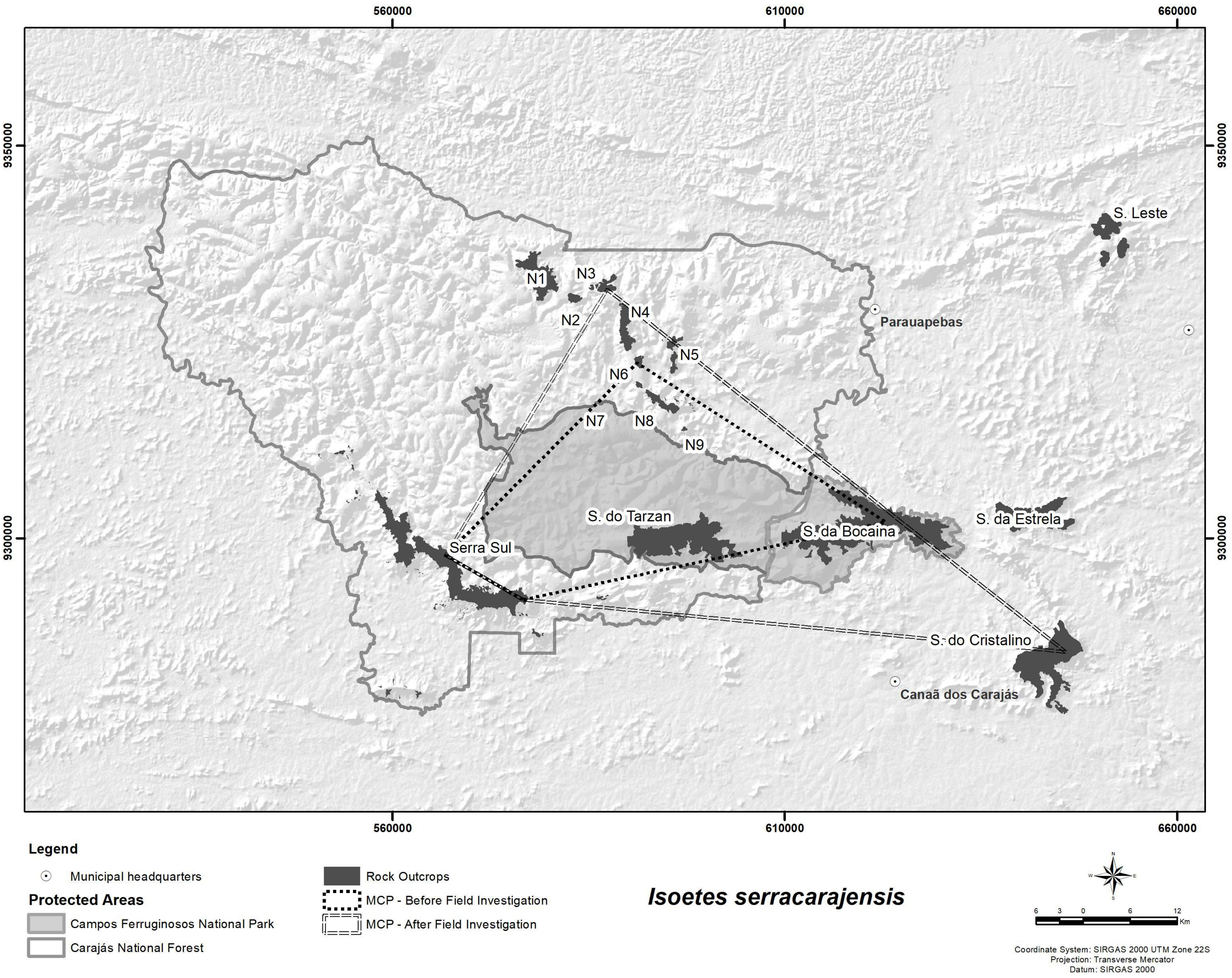
560000
610000
660000
9350000
9300000
S. Leste
N1
N3
N2
N4
N5
N6
N7
N8
N9
Parauapebas
S. do Tarzan
S. da Bocaina
S. da Estrela
Serra Sul
S. do Cristalino
Canaã dos Carajás
Legend
Municipal headquarters
Protected Areas
Campos Ferruginosos National Park
Carajás National Forest
Rock Outcrops
MCP - Before Field Investigation
MCP - After Field Investigation
Isoetes serracarajensis
N
W
E
S
6 3 0 6 12
Km
Coordinate System: SIRGAS 2000 UTM Zone 22S
Projection: Transverse Mercator
Datum: SIRGAS 2000

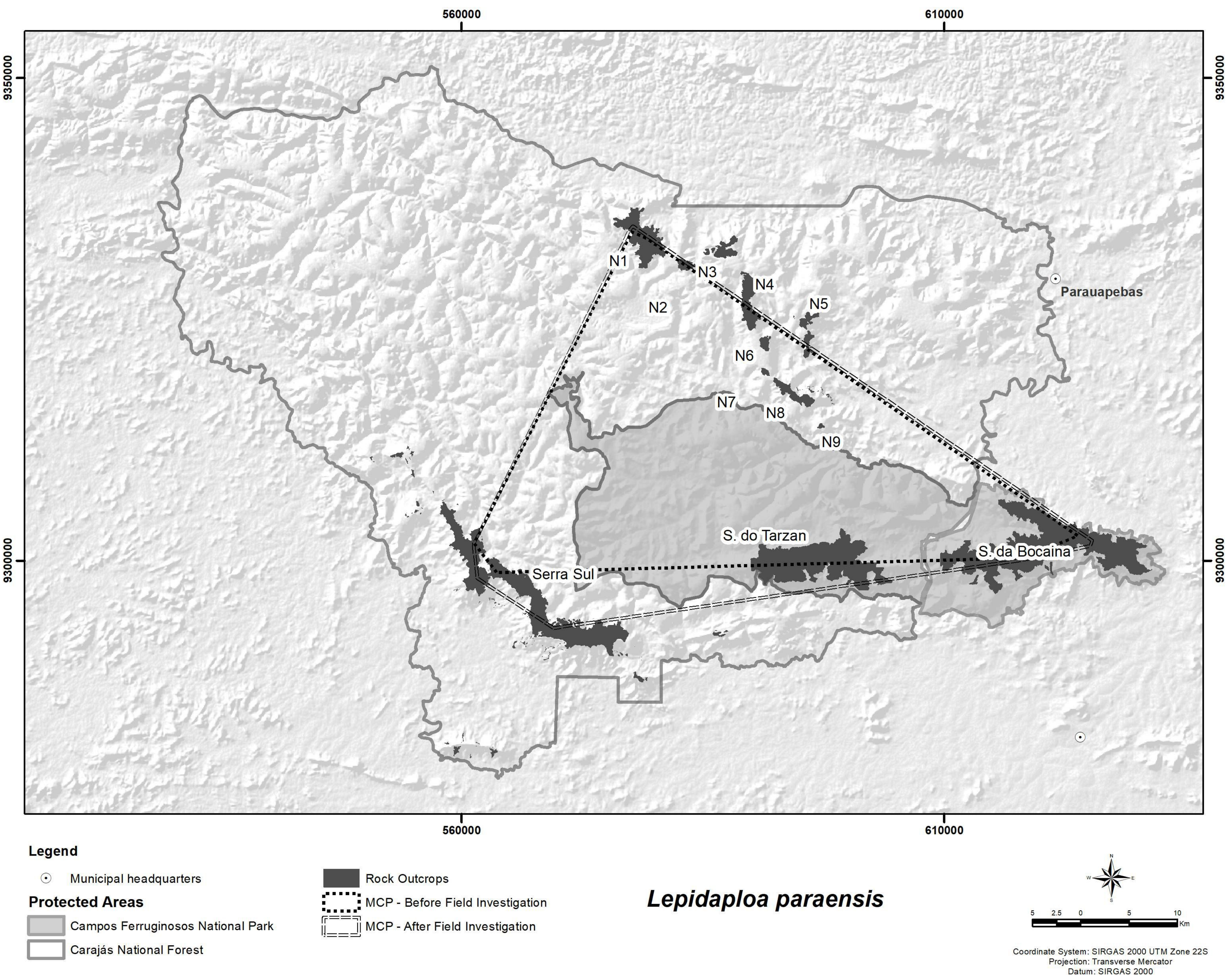

560000
610000
9350000
9300000
N1
N2
N3
N4
N5
N6
N7
N8
N9
Parauapebas
S. do Tarzan
S. da Bocaina
Serra Sul
Legend
Municipal headquarters
Protected Areas
Campos Ferruginosos National Park
Carajás National Forest
Rock Outcrops
MCP - Before Field Investigation
MCP - After Field Investigation
Lepidaploa paraensis
N
W
E
S
5 2.5 0 5 10
Km
Coordinate System: SIRGAS 2000 UTM Zone 22S
Projection: Transverse Mercator
Datum: SIRGAS 2000

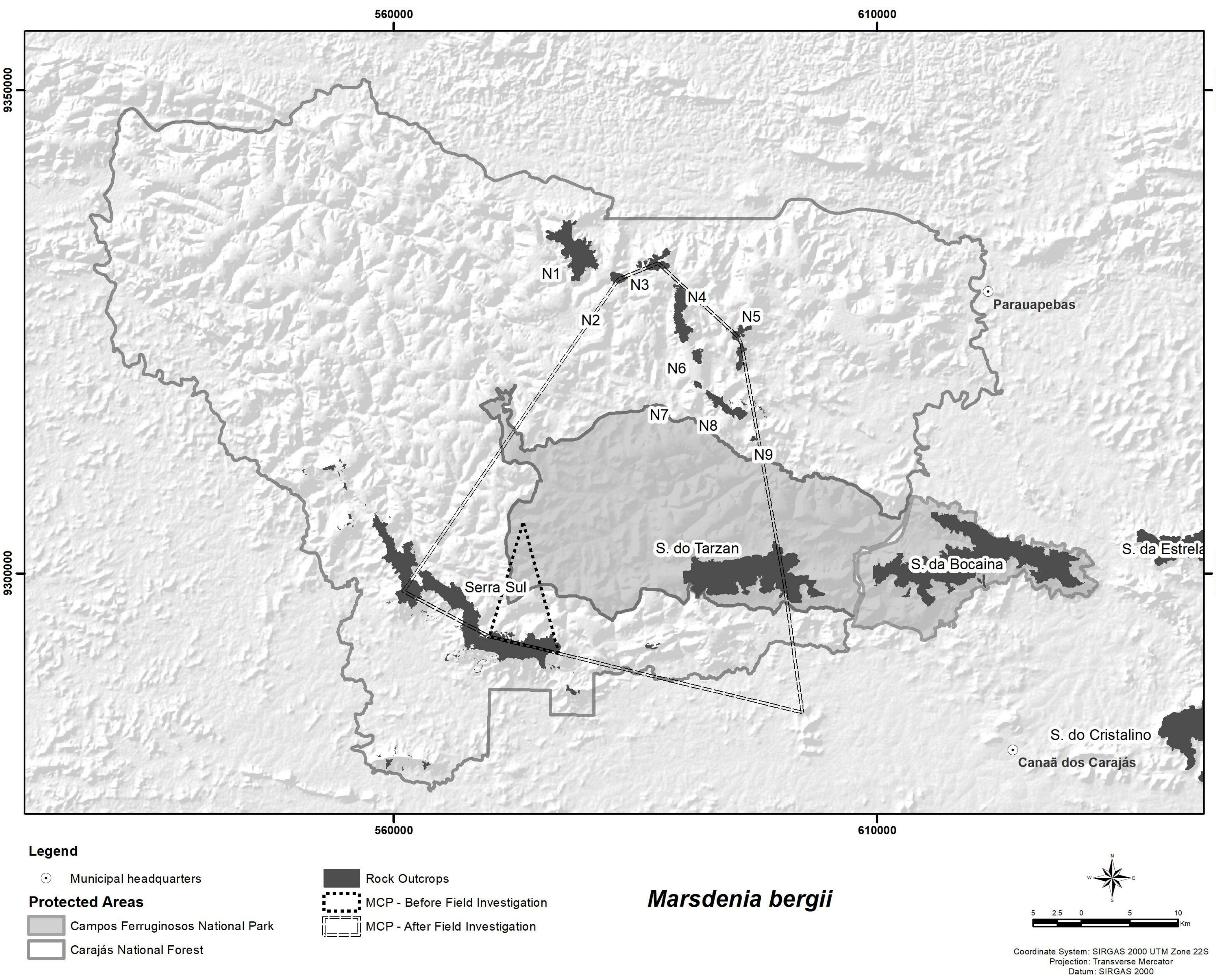
560000
610000
9350000
9300000
N1
N2
N3
N4
N5
N6
N7
N8
N9
Parauapebas
Serra Sul
S. do Tarzan
S. da Bocaina
S. da Estrela
S. do Cristalino
Canaã dos Carajás
Legend
Municipal headquarters
Protected Areas
Campos Ferruginosos National Park
Carajás National Forest
Rock Outcrops
MCP - Before Field Investigation
MCP - After Field Investigation
Marsdenia bergii
5 2.5 0 5 10
Km
Coordinate System: SIRGAS 2000 UTM Zone 22S
Projection: Transverse Mercator
Datum: SIRGAS 2000

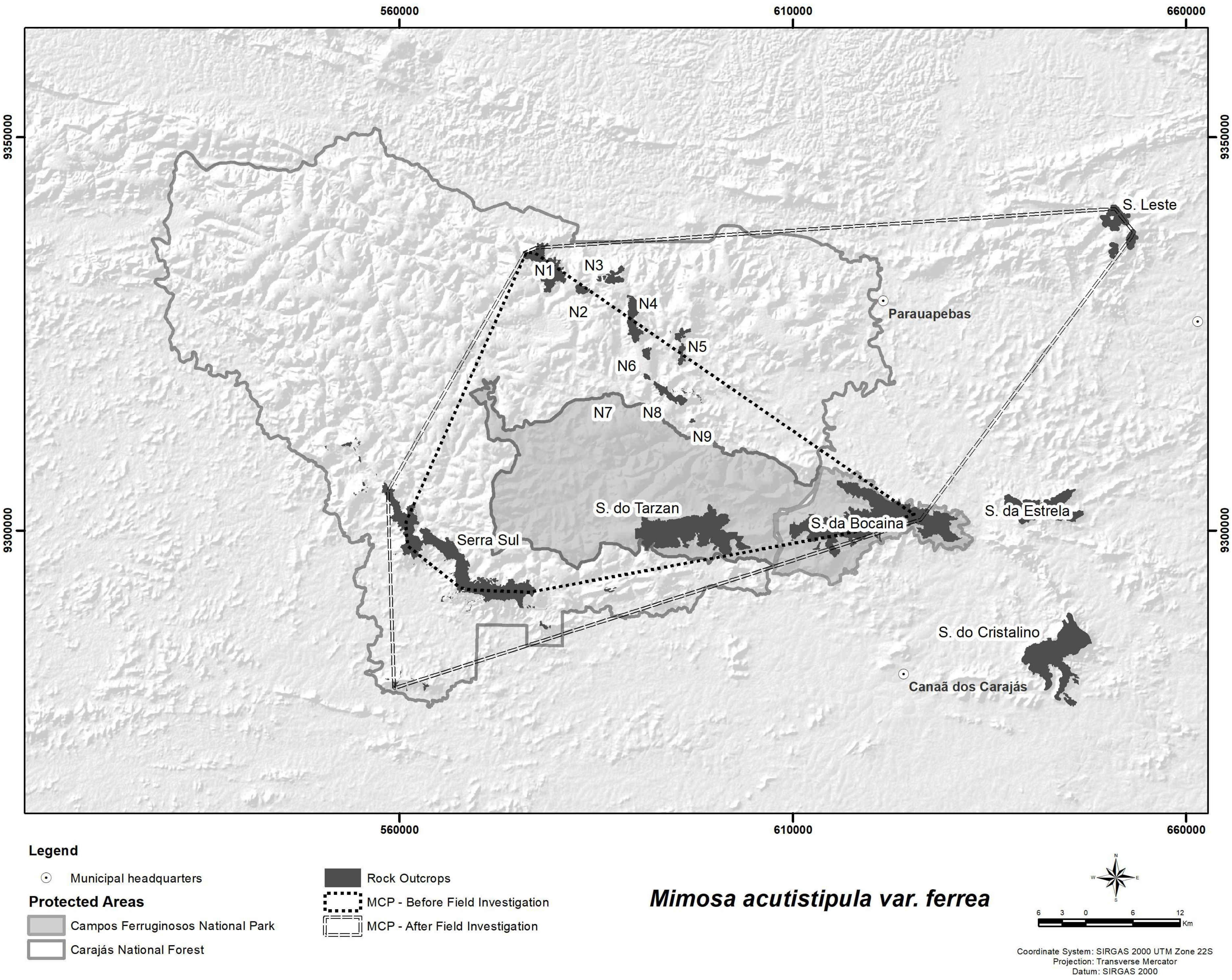
560000
610000
660000
9350000
9300000
S. Leste
N1
N2
N3
N4
N5
N6
N7
N8
N9
Parauapebas
S. do Tarzan
S. da Bocaina
S. da Estrela
Serra Sul
S. do Cristalino
Canaã dos Carajás
Legend
Municipal headquarters
Protected Areas
Campos Ferruginosos National Park
Carajás National Forest
Rock Outcrops
MCP - Before Field Investigation
MCP - After Field Investigation
Mimosa acutistipula var. ferrea
6 3 0 6 12 Km
Coordinate System: SIRGAS 2000 UTM Zone 22S
Projection: Transverse Mercator
Datum: SIRGAS 2000

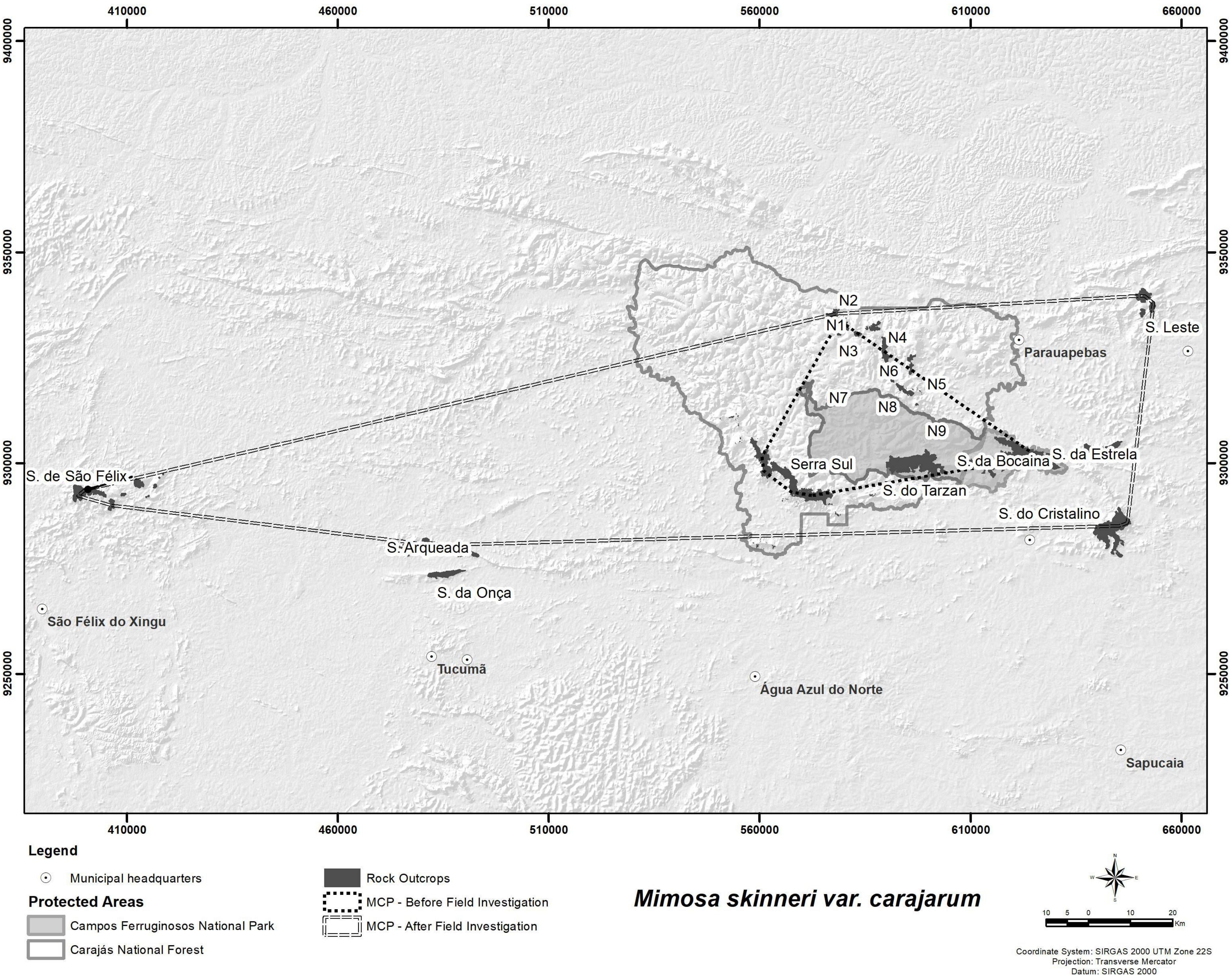
410000
460000
510000
560000
610000
660000
9400000
9350000
9300000
9250000
N2
N1
N4
N3
N6
N5
N7
N8
N9
S. Leste
Parauapebas
S. de São Félix
Serra Sul
S. da Bocaina
S. da Estrela
S. do Tarzan
S. do Cristalino
S. Arqueada
S. da Onça
São Félix do Xingu
Tucumã
Água Azul do Norte
Sapucaia
Legend
Municipal headquarters
Protected Areas
Campos Ferruginosos National Park
Carajás National Forest
Rock Outcrops
MCP - Before Field Investigation
MCP - After Field Investigation
Mimosa skinneri var. carajarum
10 5 0 10 20
Km
Coordinate System: SIRGAS 2000 UTM Zone 22S
Projection: Transverse Mercator
Datum: SIRGAS 2000

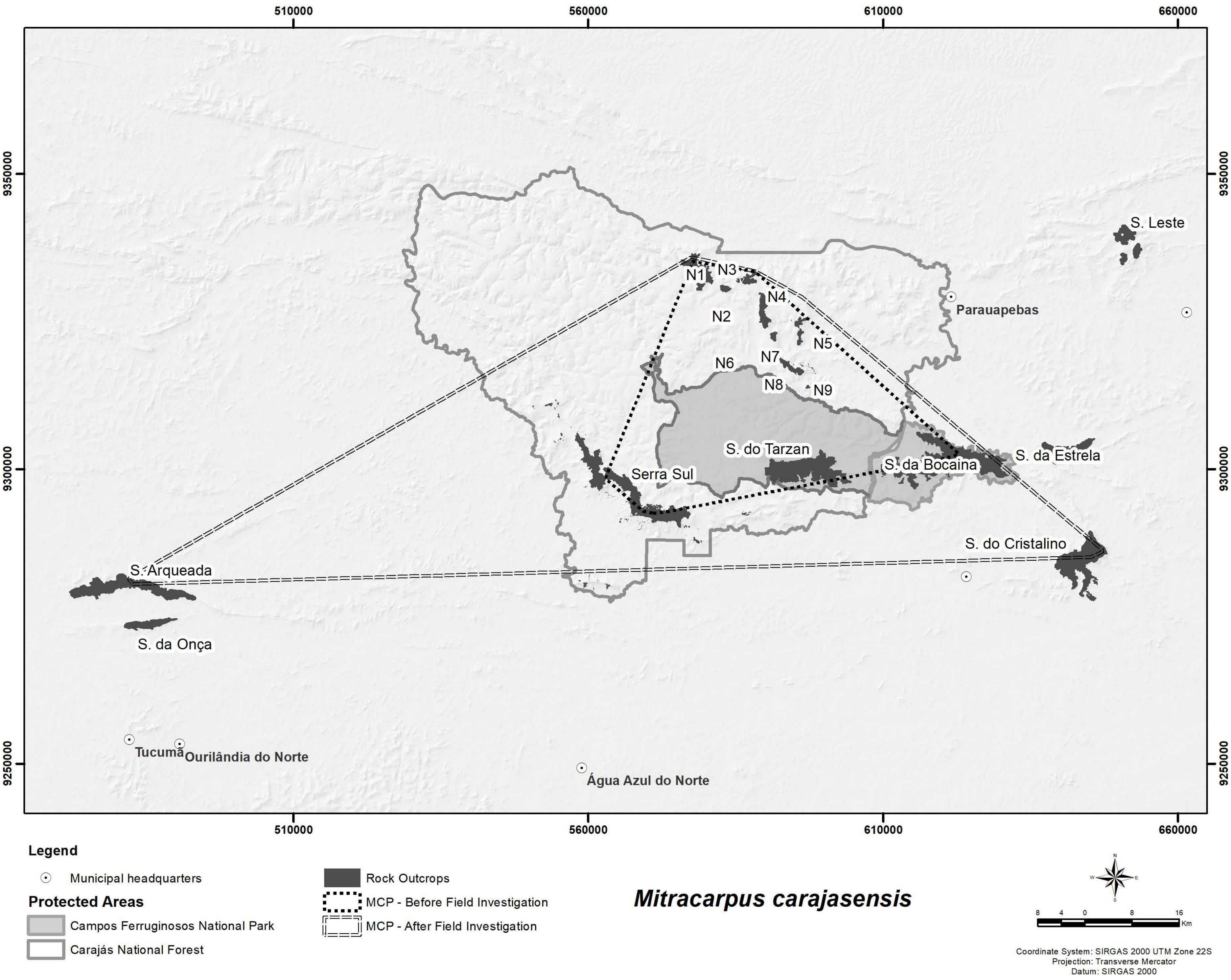
510000
560000
610000
660000
9350000
9300000
9250000
S. Leste
N1
N3
N4
N2
N5
N6
N7
N8
N9
Parauapebas
S. do Tarzan
S. da Bocaina
S. da Estrela
Serra Sul
S. do Cristalino
S. Arqueada
S. da Onça
Tucumã
Ourilândia do Norte
Água Azul do Norte
Legend
Municipal headquarters
Protected Areas
Campos Ferruginosos National Park
Carajás National Forest
Rock Outcrops
MCP - Before Field Investigation
MCP - After Field Investigation
Mitracarpus carajasensis
N
W
E
S
8 4 0 8 16
Km
Coordinate System: SIRGAS 2000 UTM Zone 22S
Projection: Transverse Mercator
Datum: SIRGAS 2000

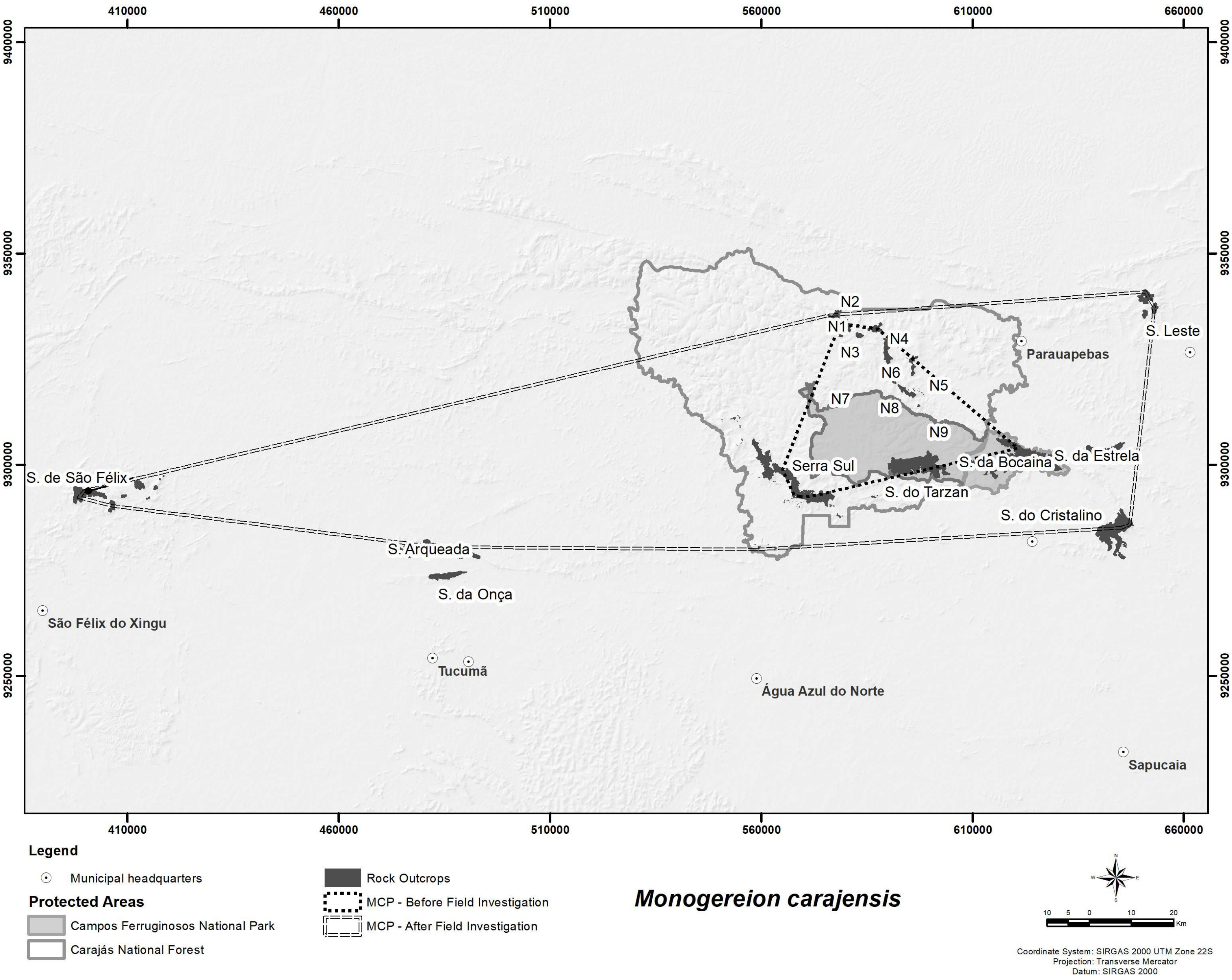

Monogereion carajensis

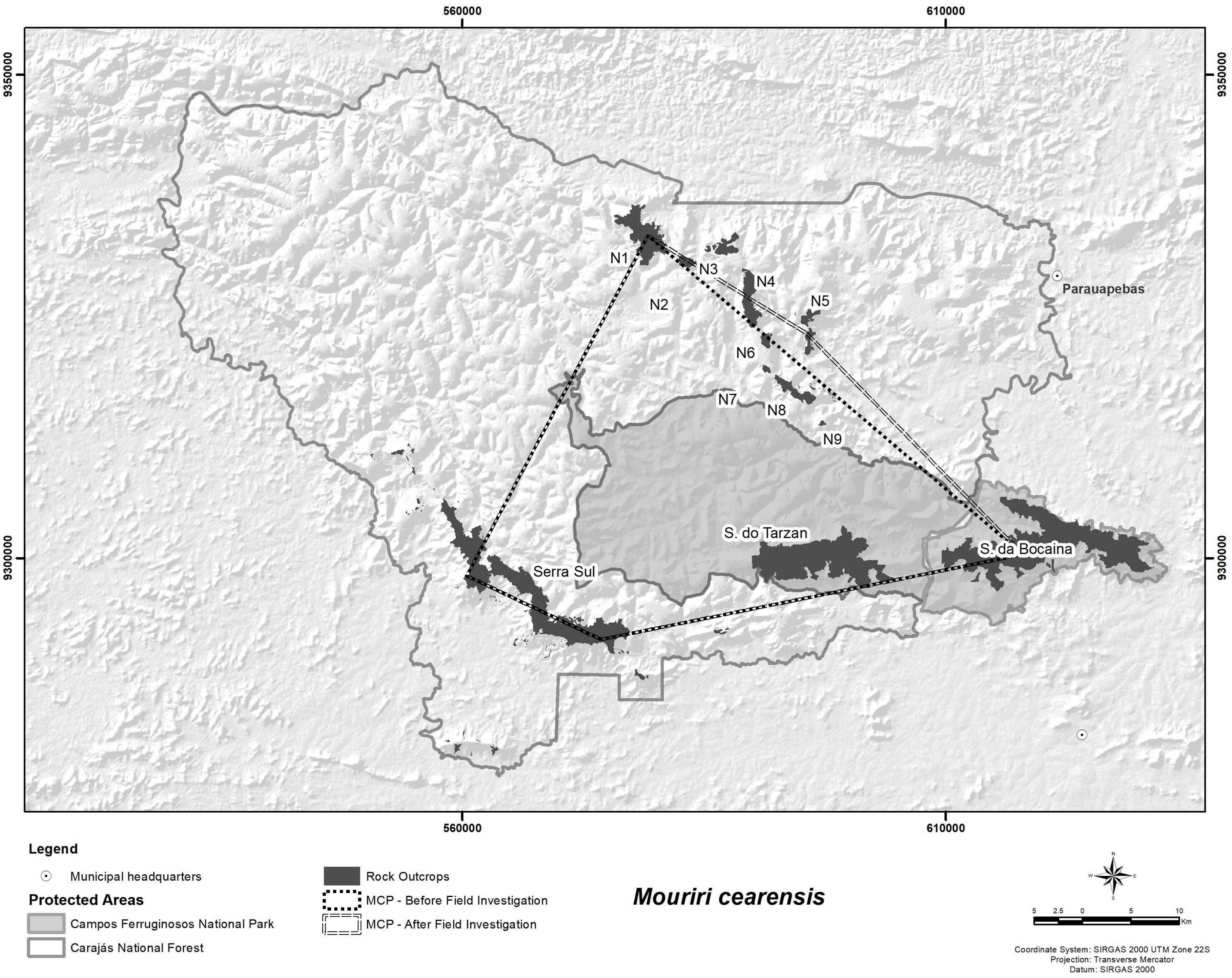
560000
610000
9350000
9300000
N1
N2
N3
N4
N5
N6
N7
N8
N9
Parauapebas
S. do Tarzan
S. da Bocaina
Serra Sul
Legend
Municipal headquarters
Protected Areas
Campos Ferruginosos National Park
Carajás National Forest
Rock Outcrops
MCP - Before Field Investigation
MCP - After Field Investigation
Mouriri cearensis
N
W
E
S
5 2.5 0 5 10
Km
Coordinate System: SIRGAS 2000 UTM Zone 22S
Projection: Transverse Mercator
Datum: SIRGAS 2000

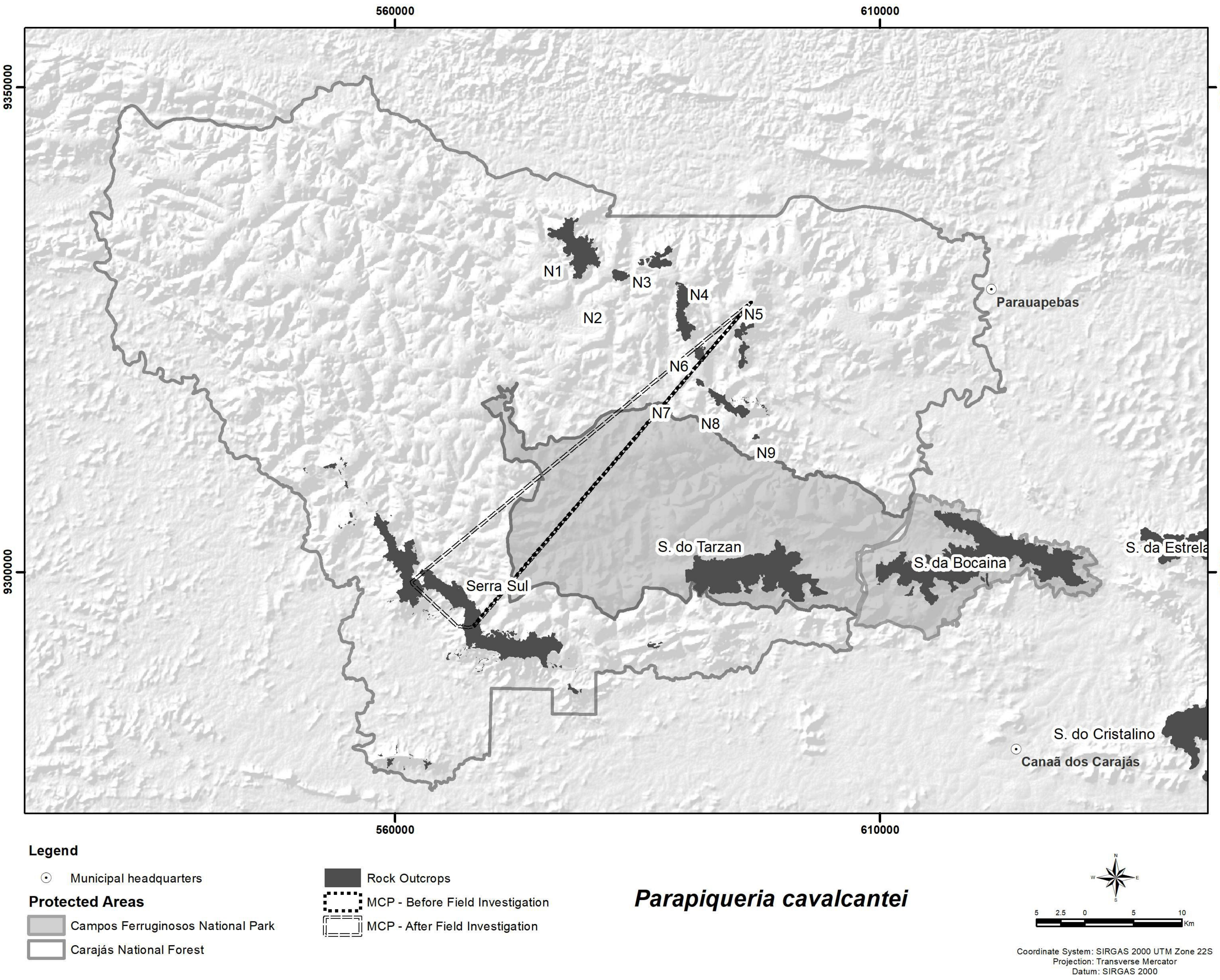
560000
610000
9350000
9300000
N1
N2
N3
N4
N5
N6
N7
N8
N9
Parauapebas
S. do Tarzan
S. da Bocaina
S. da Estrela
Serra Sul
S. do Cristalino
Canaã dos Carajás
Legend
Municipal headquarters
Protected Areas
Campos Ferruginosos National Park
Carajás National Forest
Rock Outcrops
MCP - Before Field Investigation
MCP - After Field Investigation
Parapiqueria cavalcantei
5 2.5 0 5 10 Km
Coordinate System: SIRGAS 2000 UTM Zone 22S
Projection: Transverse Mercator
Datum: SIRGAS 2000

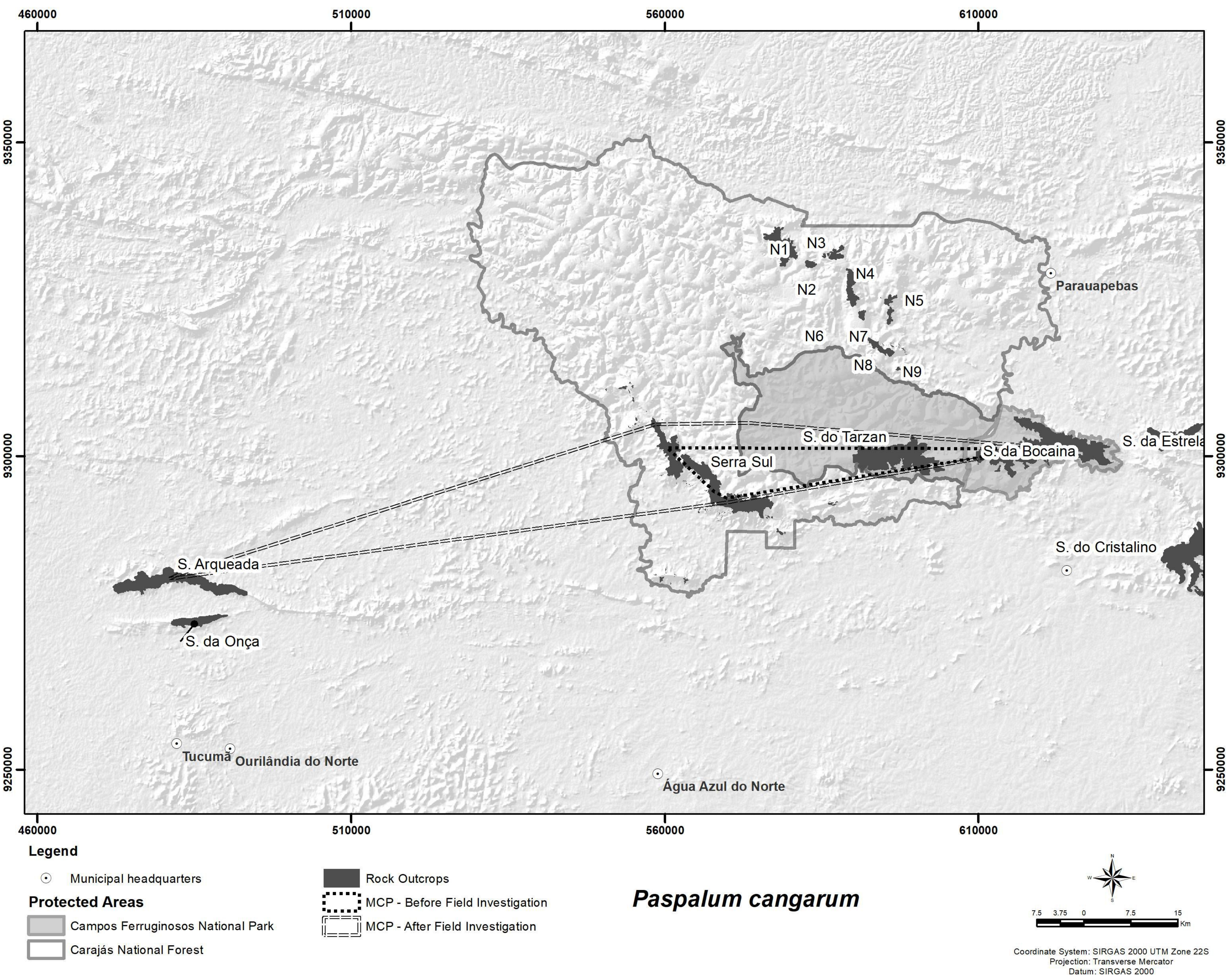

Paspalum cangarum

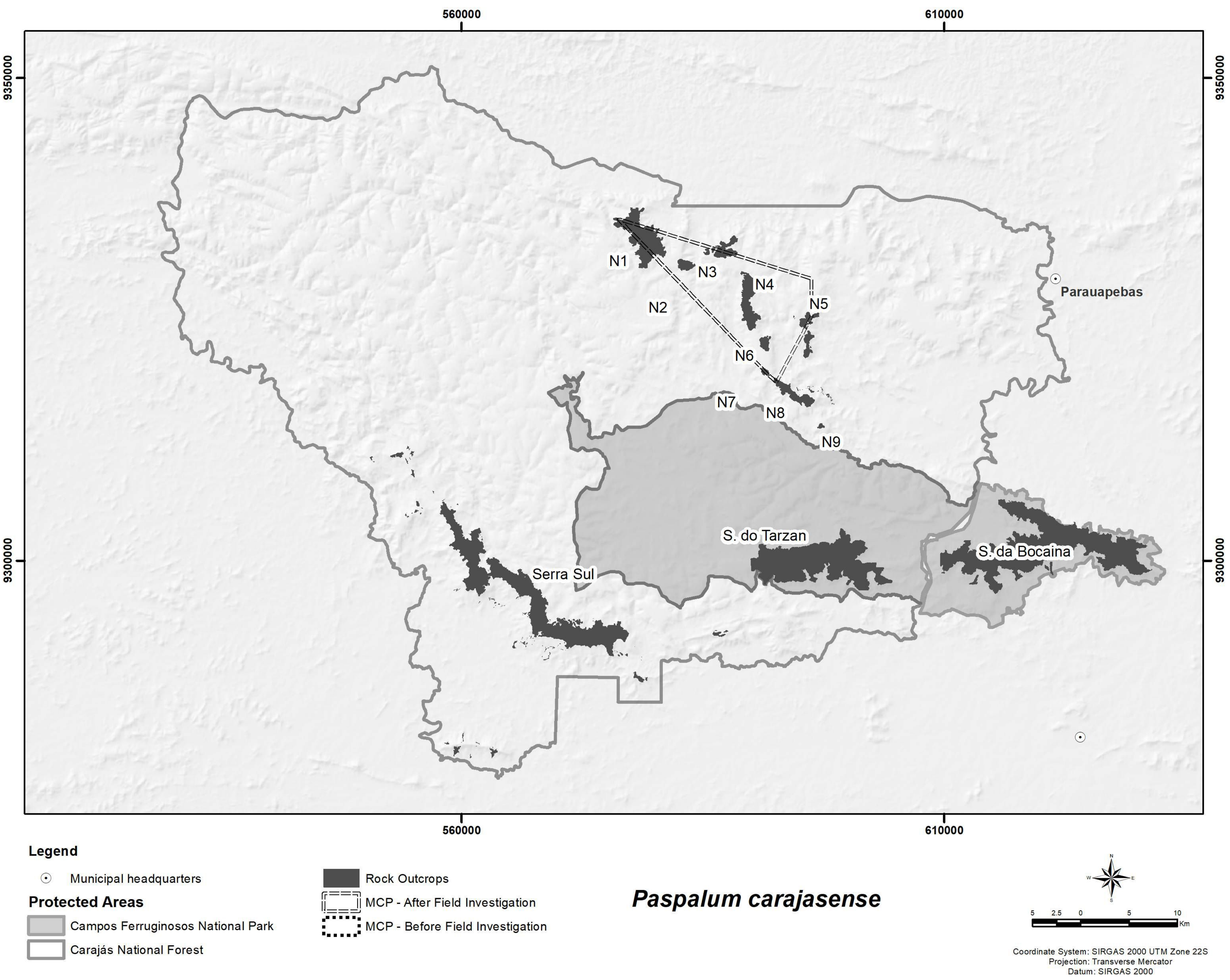

560000
610000
9350000
9300000
N1
N2
N3
N4
N5
N6
N7
N8
N9
Parauapebas
S. do Tarzan
S. da Bocaina
Serra Sul
Legend
Municipal headquarters
Protected Areas
Campos Ferruginosos National Park
Carajás National Forest
Rock Outcrops
MCP - After Field Investigation
MCP - Before Field Investigation
Paspalum carajasense
5 2.5 0 5 10
Km
Coordinate System: SIRGAS 2000 UTM Zone 22S
Projection: Transverse Mercator
Datum: SIRGAS 2000

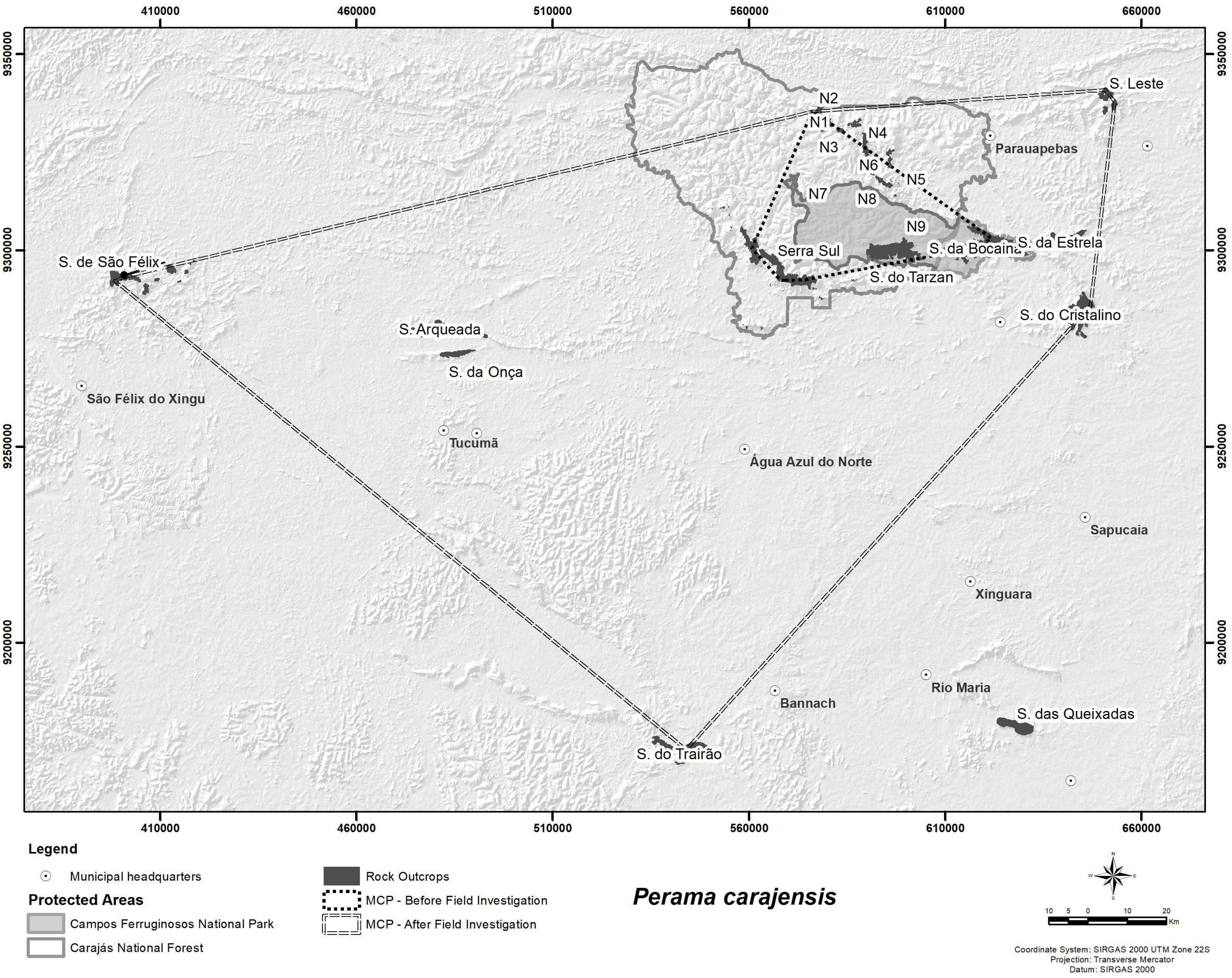

410000
460000
510000
560000
610000
660000
9350000
9300000
9250000
9200000
S. Leste
N2
N1
N4
N3
N6
N5
Parauapebas
N7
N8
N9
Serra Sul
S. da Bocaina
S. da Estrela
S. do Tarzan
S. de São Félix
S. do Cristalino
S. Arqueada
S. da Onça
São Félix do Xingu
Tucumã
Água Azul do Norte
Sapucaia
Xinguara
Rio Maria
Bannach
S. das Queixadas
S. do Trairão
Legend
Municipal headquarters
Protected Areas
Campos Ferruginosos National Park
Carajás National Forest
Rock Outcrops
MCP - Before Field Investigation
MCP - After Field Investigation
Perama carajensis
Km
Coordinate System: SIRGAS 2000 UTM Zone 22S
Projection: Transverse Mercator
Datum: SIRGAS 2000

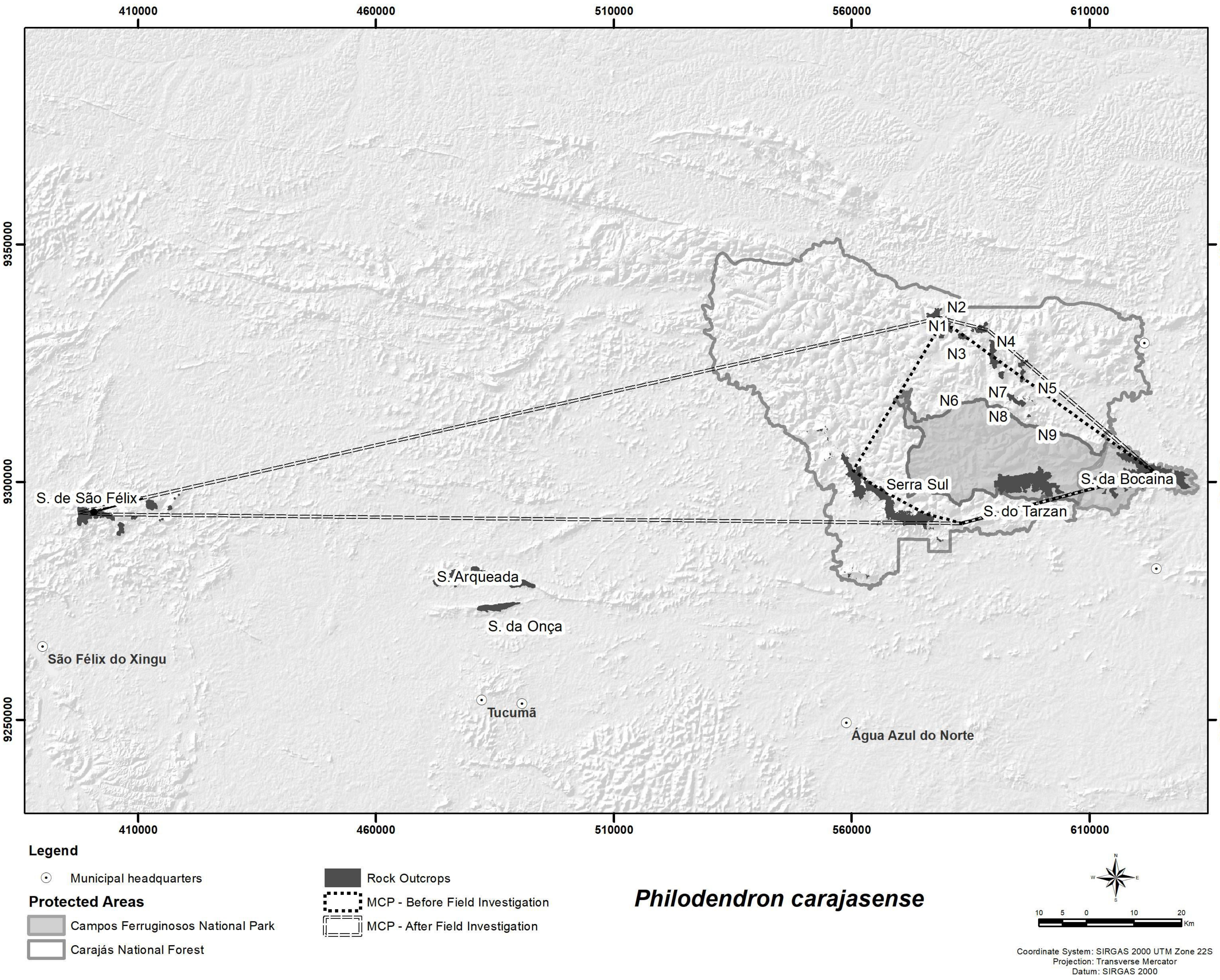

410000
460000
510000
560000
610000
9350000
9300000
9250000
N2
N1
N4
N3
N5
N6
N7
N8
N9
S. de São Félix
Serra Sul
S. da Bocaina
S. do Tarzan
S. Arqueada
S. da Onça
São Félix do Xingu
Tucumã
Água Azul do Norte
Legend
Municipal headquarters
Protected Areas
Campos Ferruginosos National Park
Carajás National Forest
Rock Outcrops
MCP - Before Field Investigation
MCP - After Field Investigation
Philodendron carajasense
10 5 0 10 20
Km
Coordinate System: SIRGAS 2000 UTM Zone 22S
Projection: Transverse Mercator
Datum: SIRGAS 2000

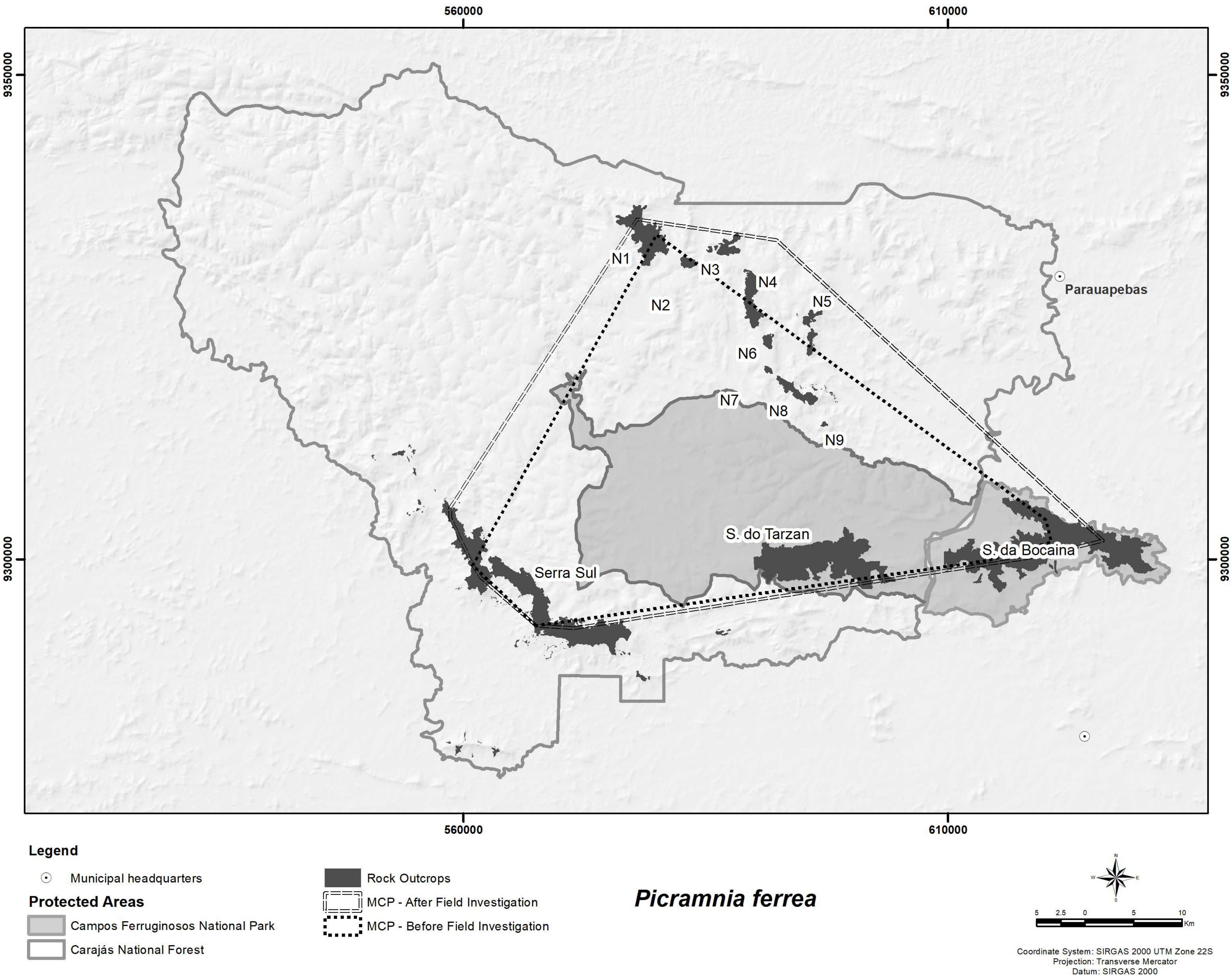
560000
610000
9350000
9300000
N1
N2
N3
N4
N5
N6
N7
N8
N9
Parauapebas
S. do Tarzan
Serra Sul
S. da Bocaina
Legend
Municipal headquarters
Protected Areas
Campos Ferruginosos National Park
Carajás National Forest
Rock Outcrops
MCP - After Field Investigation
MCP - Before Field Investigation
Picramnia ferrea
N
W
E
S
5 2.5 0 5 10
Km
Coordinate System: SIRGAS 2000 UTM Zone 22S
Projection: Transverse Mercator
Datum: SIRGAS 2000

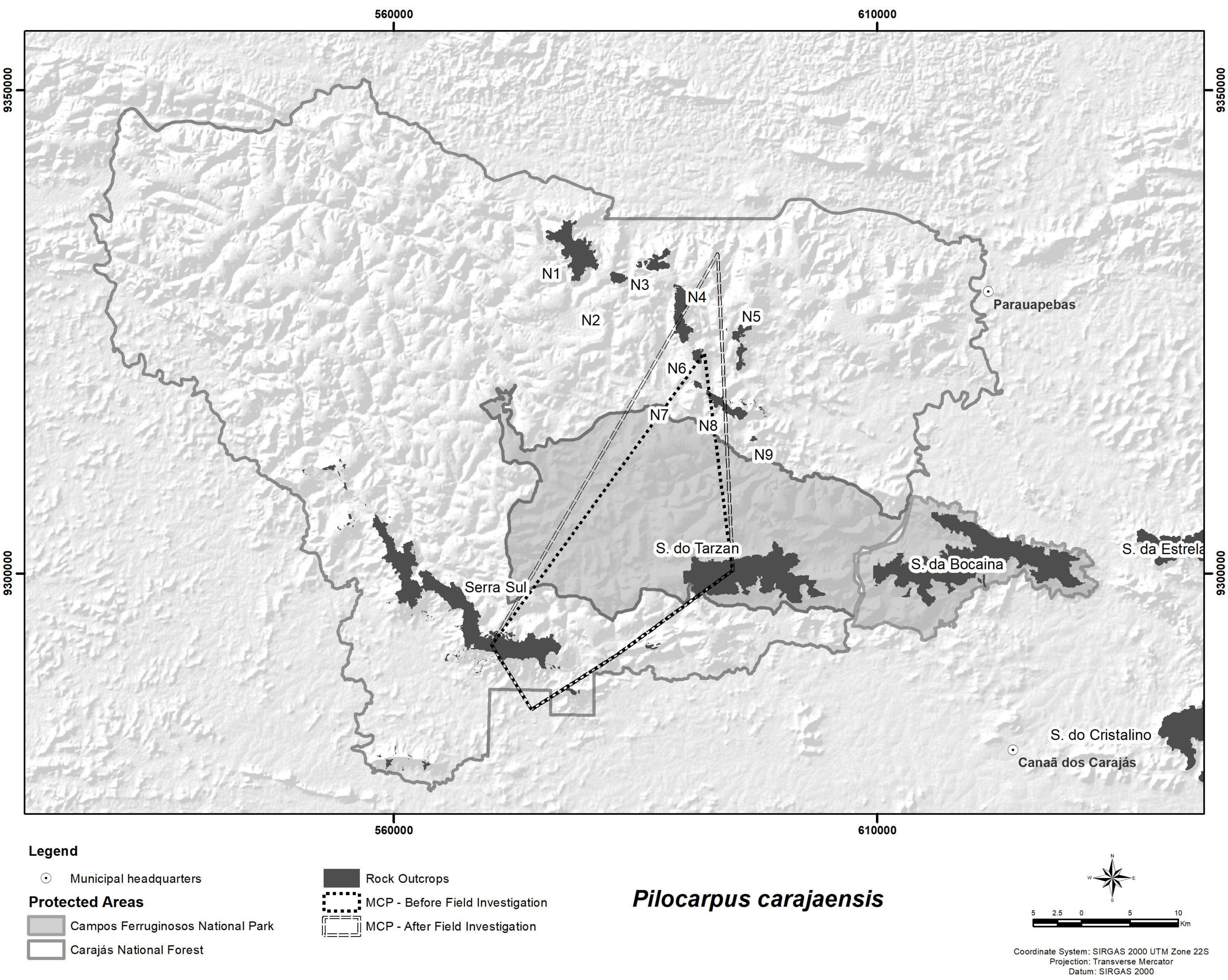
560000
610000
9350000
9300000
N1
N2
N3
N4
N5
N6
N7
N8
N9
Parauapebas
S. do Tarzan
S. da Bocaina
S. da Estrela
Serra Sul
S. do Cristalino
Canaã dos Carajás
Legend
Municipal headquarters
Protected Areas
Campos Ferruginosos National Park
Carajás National Forest
Rock Outcrops
MCP - Before Field Investigation
MCP - After Field Investigation
Pilocarpus carajaensis
5 2.5 0 5 10
Km
Coordinate System: SIRGAS 2000 UTM Zone 22S
Projection: Transverse Mercator
Datum: SIRGAS 2000

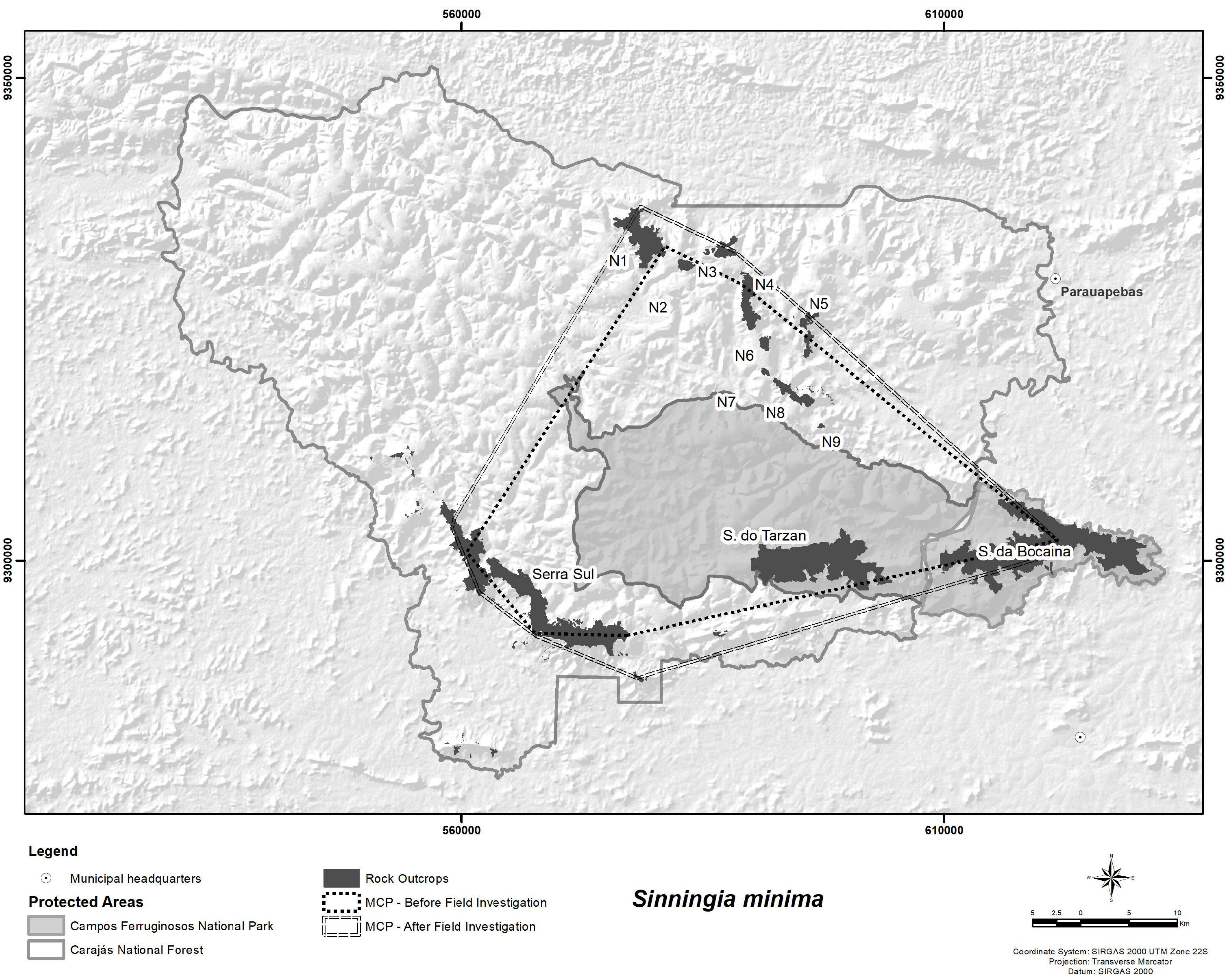
560000
610000
9350000
9300000
N1
N2
N3
N4
N5
N6
N7
N8
N9
Parauapebas
S. do Tarzan
S. da Bocaina
Serra Sul
Legend
Municipal headquarters
Protected Areas
Campos Ferruginosos National Park
Carajás National Forest
Rock Outcrops
MCP - Before Field Investigation
MCP - After Field Investigation
Sinningia minima
5 2.5 0 5 10
Km
Coordinate System: SIRGAS 2000 UTM Zone 22S
Projection: Transverse Mercator
Datum: SIRGAS 2000

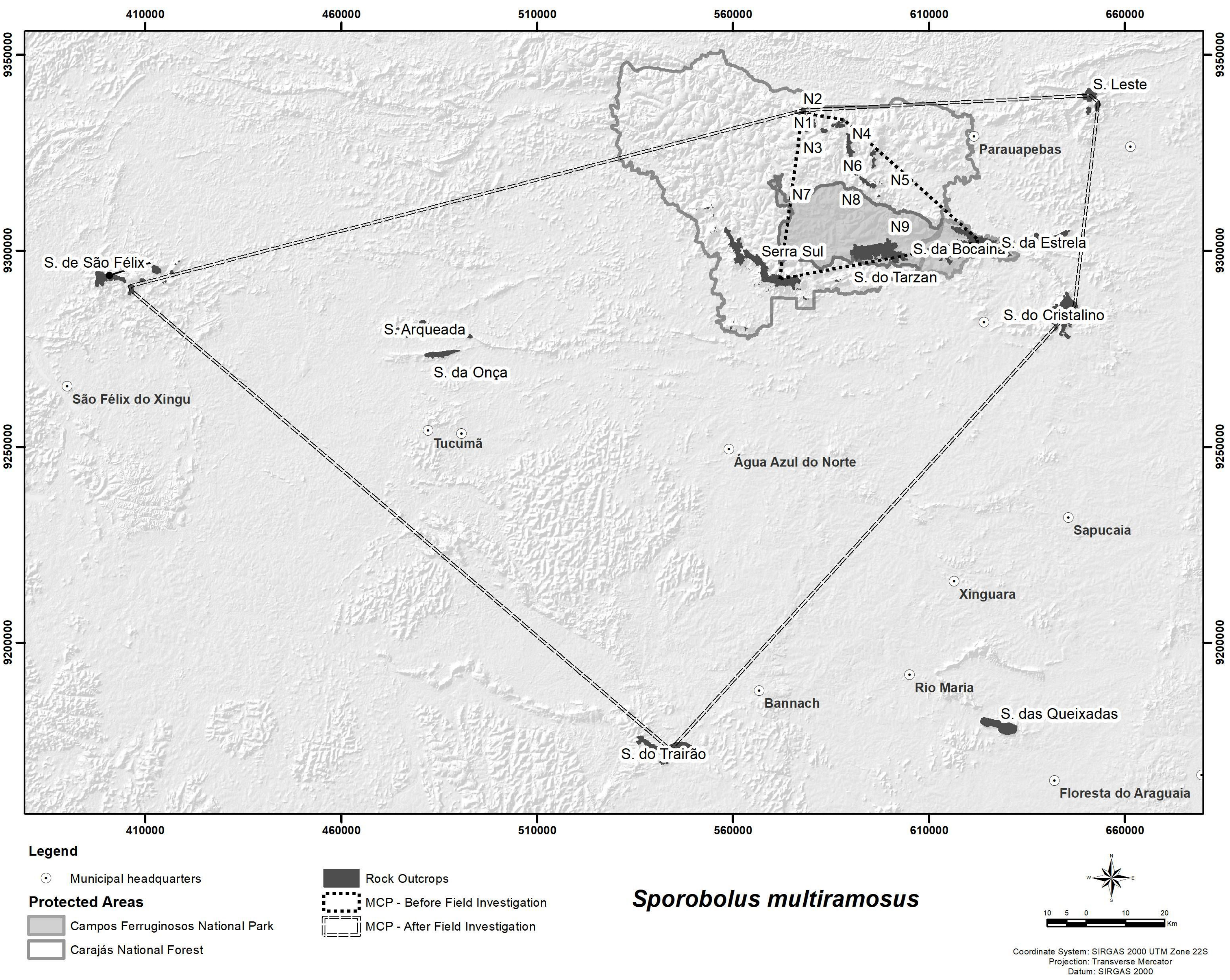

410000
460000
510000
560000
610000
660000
9350000
9300000
9250000
9200000
S. Leste
N2
N1
N4
N3
Parauapebas
N6
N5
N7
N8
N9
S. da Estrela
S. da Bocaina
Serra Sul
S. do Tarzan
S. de São Félix
S. do Cristalino
S. Arqueada
S. da Onça
São Félix do Xingu
Tucumã
Água Azul do Norte
Sapucaia
Xinguara
Rio Maria
Bannach
S. das Queixadas
S. do Trairão
Floresta do Araguaia
Legend
Municipal headquarters
Protected Areas
Campos Ferruginosos National Park
Carajás National Forest
Rock Outcrops
MCP - Before Field Investigation
MCP - After Field Investigation
Sporobolus multiramosus
10 5 0 10 20
Km
Coordinate System: SIRGAS 2000 UTM Zone 22S
Projection: Transverse Mercator
Datum: SIRGAS 2000

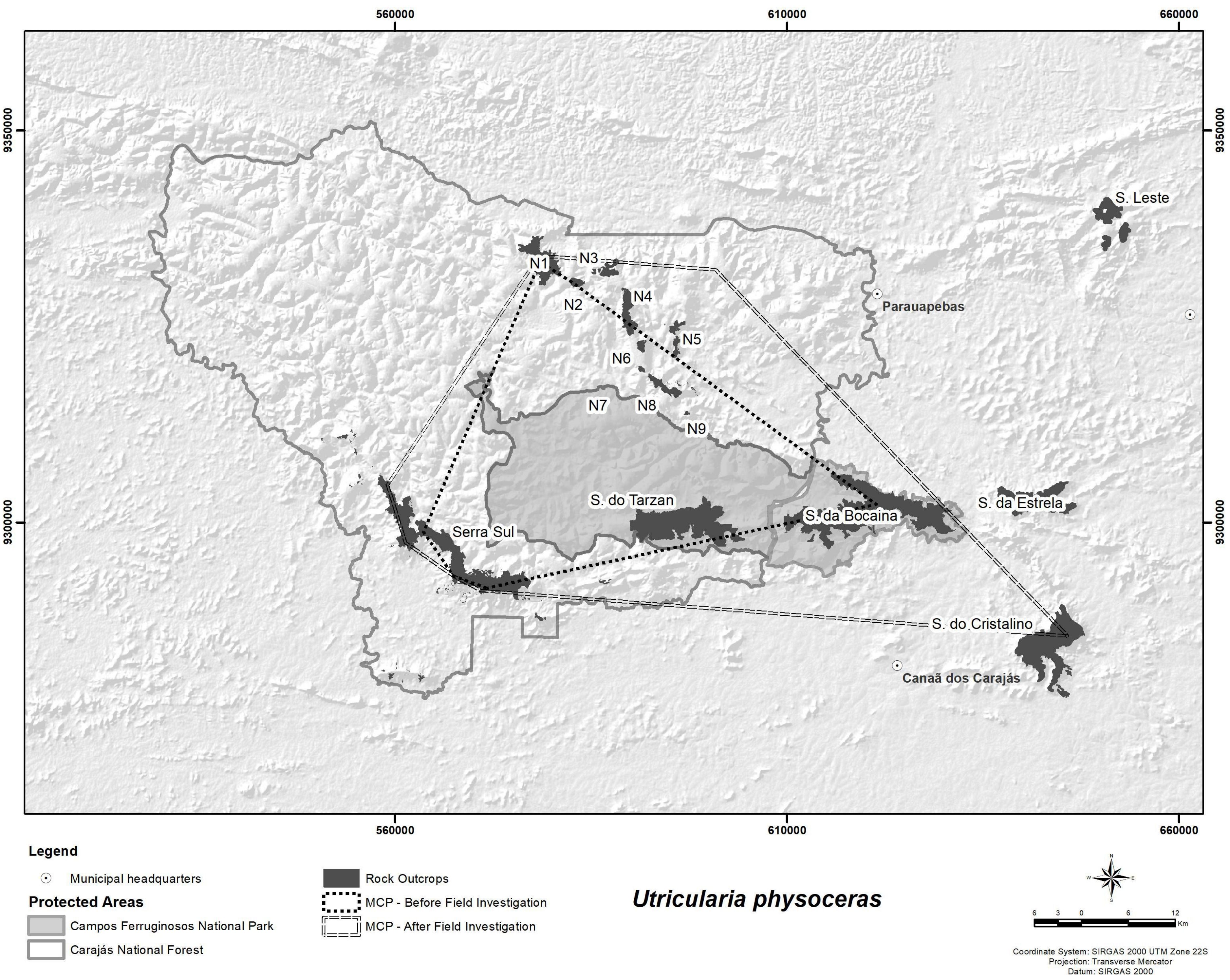
560000
610000
660000
9350000
9300000
S. Leste
N1
N3
N2
N4
N5
N6
N7
N8
N9
Parauapebas
S. do Tarzan
S. da Bocaina
S. da Estrela
Serra Sul
S. do Cristalino
Canaã dos Carajás
Legend
Municipal headquarters
Protected Areas
Campos Ferruginosos National Park
Carajás National Forest
Rock Outcrops
MCP - Before Field Investigation
MCP - After Field Investigation
Utricularia physoceras
N
W
E
S
6 3 0 6 12
Km
Coordinate System: SIRGAS 2000 UTM Zone 22S
Projection: Transverse Mercator
Datum: SIRGAS 2000

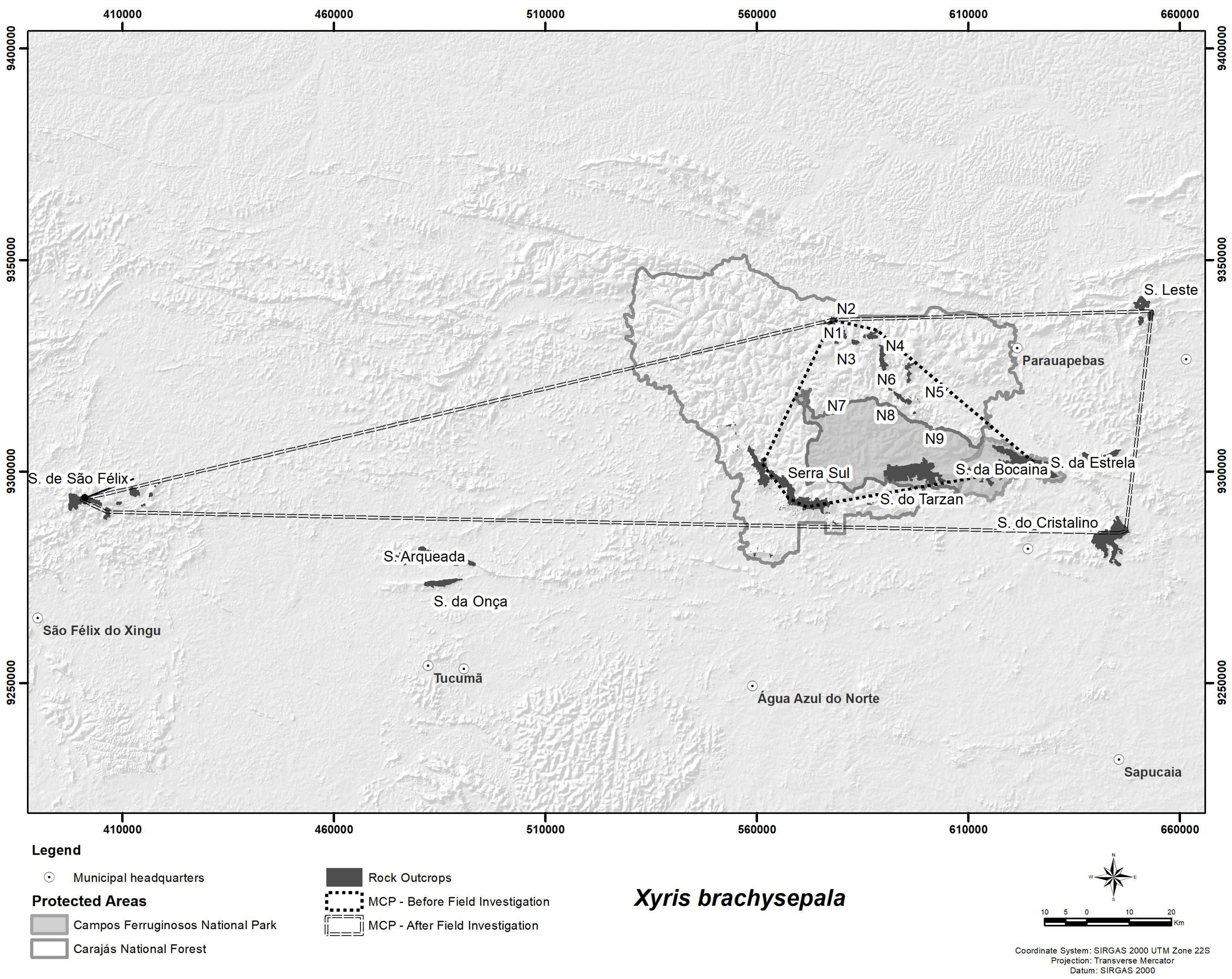
410000
460000
510000
560000
610000
660000
9400000
9350000
9300000
9250000
S. Leste
N2
N1
N4
N3
N6
N5
N7
N8
N9
Parauapebas
S. de São Félix
Serra Sul
S. da Bocaina
S. da Estrela
S. do Tarzan
S. do Cristalino
S. Arqueada
S. da Onça
São Félix do Xingu
Tucumã
Água Azul do Norte
Sapucaia
Legend
Municipal headquarters
Protected Areas
Campos Ferruginosos National Park
Carajás National Forest
Rock Outcrops
MCP - Before Field Investigation
MCP - After Field Investigation
Xyris brachysepala
10 5 0 10 20
Km
Coordinate System: SIRGAS 2000 UTM Zone 22S
Projection: Transverse Mercator
Datum: SIRGAS 2000
